# Accelerated evolutionary rates in tropical and oceanic parmelioid lichens (Ascomycota)

**DOI:** 10.1186/1471-2148-8-257

**Published:** 2008-09-22

**Authors:** H Thorsten Lumbsch, Andrew L Hipp, Pradeep K Divakar, Oscar Blanco, Ana Crespo

**Affiliations:** 1 Department of Botany, The Field Museum, 1400 S. Lake Shore Drive, Chicago, IL 60605, USA; 2 The Morton Arboretum, 4100 Illinois Route 53, Lisle, IL 60532, USA; 3 Departamento de Biología Vegetal II, Facultad de Farmacia, Universidad Complutense de Madrid, Madrid 28040, Spain

## Abstract

**Background:**

The rate of nucleotide substitutions is not constant across the Tree of Life, and departures from a molecular clock have been commonly reported. Within parmelioid lichens, the largest group of macrolichens, large discrepancies in branch lengths between clades were found in previous studies. Using an extended taxon sampling, we test for presence of significant rate discrepancies within and between these clades and test our a priori hypothesis that such rate discrepancies may be explained by shifts in moisture regime or other environmental conditions.

**Results:**

In this paper, the first statistical evidence for accelerated evolutionary rate in lichenized ascomycetes is presented. Our results give clear evidence for a faster rate of evolution in two *Hypotrachyna *clades that includes species occurring in tropical and oceanic habitats in comparison with clades consisting of species occurring in semi-arid and temperate habitats. Further we explore potential links between evolutionary rates and shifts in habitat by comparing alternative Ornstein-Uhlenbeck models.

**Conclusion:**

Although there was only weak support for a shift at the base of a second tropical clade, where the observed nucleotide substitution rate is high, overall support for a shift in environmental conditions at cladogenesis is very strong. This suggests that speciation in some lichen clades has proceeded by dispersal into a novel environment, followed by radiation within that environment. We found moderate support for a shift in moisture regime at the base of one tropical clade and a clade occurring in semi-arid regions and a shift in minimum temperature at the base of a boreal-temperate clade.

## Background

Differences in nucleotide substitution rates among taxa are a common phenomenon in molecular studies [1-7], and the presence of an exact molecular clock [8,9] appears to be a rare exception in molecular evolution, if present at all. A number of different causes are invoked to explain differences in evolutionary rates among taxa. Recently, Kay et al. [10] demonstrated correlations of substitution rates and life histories. In angiosperms, herbaceous plants have substitution rates almost twice as high as woody plants [10], while no phylogenetic constraint on rates was found in their study among lineages. Previously, higher substitution rates had been found in annual compared to perennial plants [5,11-14]. These studies in plants agree with previous studies suggesting that shorter generation times are associated with accelerated evolutionary rates [15,16].

Significant departure from constant rates of a molecular clock have so far only been demonstrated for the kingdom Fungi in Basidiomycota [17,18] and in four endosymbiotic pyrenomycetes [6]. In these cases accelerated evolutionary rates were significantly associated with mutualism. In one of the largest classes of Ascomycota, Lecanoromycetes [19,20], significant differences in nucleotide substitution rates have not yet been shown. However, in previous studies [21,22] we have found remarkable differences in branch lengths between clades in parmelioid lichens (Parmeliaceae, Lecanoromycetes). Taxa of one clade (the *Hypotrachyna *clade) had consistently longer branches in phylogenetic trees than taxa in other clades, which suggested an accelerated evolutionary rate in that group.

Parmelioid lichens are the largest group within Parmeliaceae [23], which itself is one of the largest families of lichen-forming fungi and has a worldwide distribution. The Parmelioid group comprises common and well-known species, including taxa such as *Parmelia sulcata*, *Flavoparmelia caperata*, and *Punctelia borreri*, which are frequently used in biomonitoring atmospheric pollution [24,25]. The group has approximately 1500 taxa [26,27]. It includes species which are mainly foliose, mostly rhizinate lichens with laminal apothecia and simple, hyaline ascospores [28]. Recent phylogenetic studies have shown that a core group of parmelioid lichens is monophyletic [21-23]. Within this group, seven well-supported clades were found [21], which show distinct ecological preferences. For example, taxa belonging to the *Hypotrachyna *clade (c. 300 species) are mostly tropical extending into temperate regions in moist, oceanic habitats [29-31]. In contrast, species of the *Xanthoparmelia *clade (c. 700 species) [32,33] have their center of distribution in semi-arid regions.

Lichens are poikilohydric organisms and are able to survive long periods of dry conditions in a dormant stage [34-40]. They lack stomata and any water storage system. Water vapor is lost readily from across the whole surface of lichens. In some lichens, alteration between desiccated and hydrated stages is an almost daily occurrence [41-44]. This has an important ecological consequence, since the duration of physiological activity of lichens is directly dependent on the ability of water in a habitat. Lichens in dry habitats may be physiologically active only for a few hours a day and dormant most of the day [45-49], while lichens growing in moist habitats usually have sufficient water content all day long for physiological activity [50-52].

To further investigate the remarkable differences in branch lengths between species of the *Hypotrachyna *clade (including tropical species extending into temperate regions in moist, oceanic habitats) and other clades of parmelioid lichens [21] we obtained sequence data from additional species and/or genes to perform a study focusing on the differences in evolutionary rates within parmelioid lichens and potential causes for rate shifts. Sequences of two nuclear (ITS, nu LSU) and single mitochondrial ribosomal (mtSSU) DNA loci are used to infer phylogenetic relationships. These genes have been successfully used to infer phylogenies in parmelioid lichens [21,29]. Here we use a phylogenetically based maximum likelihood procedure to identify shifts in rates of nucleotide substitutions. Further we explore the potential links between rates of molecular evolution and ecological parameters by comparing alternative Ornstein-Uhlenbeck models [53]. These models allow us to test the hypothesis that cladogenesis in parmelioid lichens is associated with shifts in habitat that might in turn explain changes in nucleotide substitution rate.

## Results

### Phylogenetic Analysis

**Figure 1:**
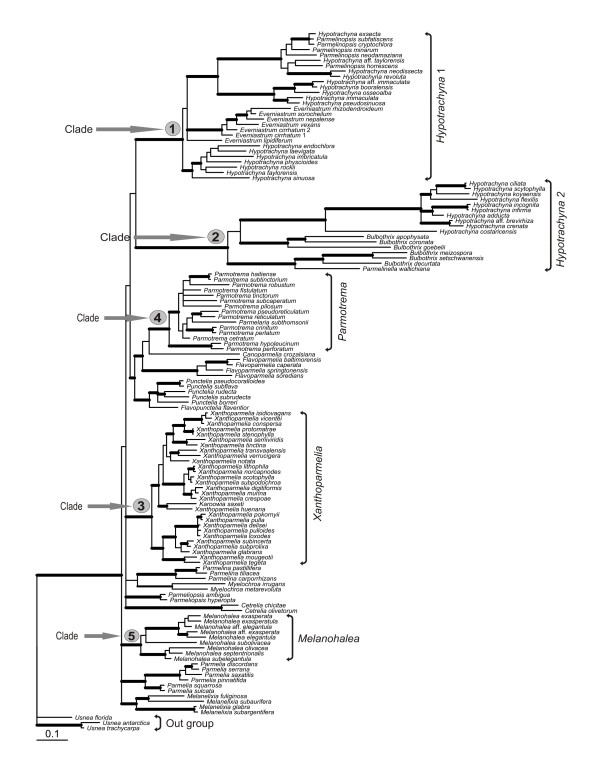
**Phylogeny of parmelioid lichens**. Phylogenetic relationships of parmelioid lichens inferred from a combined analysis of nuclear ITS, LSU, and mitochondrial SSU rDNA, sequences. 50% majority-rule consensus tree of 56,000 trees sampled using a Bayesian MC/MCMC analysis. Branches with posterior probabilities above 0.94 and also bootstrap support under parsimony equal or above 75% are indicated in bold.

Phylogenetic analyses of the three gene partitions combined included 2022 unambiguously aligned nucleotide, of which 734 were variable and 563 were parsimony informative. The topologies of the Bayesian analysis (Figure 1) and MP results were generally similar and all nodes strongly supported by MP bootstrapping (≥75%) were well supported by Bayesian pp (MP tree not shown). All strongly supported clades are also supported in the relaxed-clock analyses conducted in BEAST (data not shown). Our results were largely consistent with previous phylogenies [21-23,29,54], but with the addition of several new taxa and/or an additional gene partition. The species were placed within groups expected by their morphological and chemical characters [21,28,30,31]. However, the placement of some taxa differed slightly from previous analysis. This includes *Parmelinella wallichiana*, which had an unresolved sister-group relationship to *Bulbothrix decurtata *and a clade including *B. meizospora *and *B. setschwanensis*; *Everniastrum*, which was resolved as monophyletic in previous analyses; and a few relationships of terminal sister taxa [29]. None of the differences to previous studies involve well supported conflicts. Further, the close relationship of *Karoowia saxeti *with *Xanthoparmelia *found in previous studies [21,32] is confirmed and its placement within that genus is strongly supported for the first time.

### Significant Rate Differences

Between major clades of parmelioid lichens considerable branch length differences were evident, but also within clades branch lengths differed (Figure 1). Likelihood ratio tests comparing null models of rate constancy versus alternative models revealed statistically significant departures from rate constancy across the entire tree (Additional file 1). Since some of the well supported clades included only a few OTUs, we restricted our analysis to five clades with sufficient number of taxa (more than six OTUs). Within these five clades selected for the rate difference test, analyses of three of 15 single-gene data sets did not return significant results, meaning that a molecular clock cannot be rejected. This includes the ITS data sets of the *Melanohalea- *and *Xanthoparmelia *clades, and the mtSSU data set of the *Parmotrema *clade.

To determine rate differences between clades and to identify particular clades with deviating rates, two-rate and three-rate models were compared for three selected clades in each analysis using the likelihood ratio test statistics. Significant rate differences were found between the *Hypotrachyna *clades 1 and 2 and other clades examined (Additional file 1). Rate comparisons of the *Melanohalea *clade with the *Parmotrema *and *Xanthoparmelia *clades, respectively, and comparisons of the latter two clades revealed insignificant results.

### Habitat Shifts

The species of the five selected clades live under different ecological conditions (Table 1). On average, the species in the two *Hypotrachyna *clades occur at higher altitudes. Moreover, these and the species of the *Parmotrema *clade occupy habitats with higher precipitation and consequently have the highest Emberger Index values. In contrast, species in the *Xanthoparmelia *and *Melanohalea *clades occur in habitats with low precipitation and low Emberger's index.

**Table 1:** Ecological characters of the five examined clades of parmelioid lichens (average per year followed by standard deviation in brackets).

	Altitude	Maximal average temperature	Minimal average temperature	Precipitation (in mm)	Emberger Index
*Hypotrachyna *clade 1	1549.08 (± 1323.53)	17.56 (± 3.57)	8.38 (± 3.53)	1071.32 (± 345.32)	209.19 (± 69.07)
*Hypotrachyna *clade 2	1918 (± 992.86)	23.6 (± 2.25)	11.5 (± 7.78)	1383.35 (± 775.43)	151.98 (± 20.87)
*Melanohalea *clade	1024.22 (± 537.52)	18.51 (± 3.07)	-2.56 (± 5.06)	711 (± 522.39)	109.46 (± 77.83)
*Parmotrema *clade	349.21 (± 341.44)	20.51 (± 2.67)	10.06 (± 2.95)	1014 (± 184.93)	118.52 (± 41.66)
*Xanthoparmelia *clade	766.5 (± 497.23)	20.2 (± 2.43)	5.04 (± 2.74)	631.93 (± 209.98)	81.14 (± 33.41)

The Emberger's index is calculated as follows: Q = 100 × P/Tm_max_^2 ^- Tm_min_^2^, where P is average annual precipitation, Tm_max _is average maximum temperature of warmest month, Tm_min _is average minimum temperature of coldest month.

There is moderate support for a shift in moisture regime (represented by precipitation and Emberger's Index) at the base of *Hypotrachyna *clade 2 and the *Xanthoparmelia *clade, and strong support for a shift in minimum temperature at the base of the *Melanohalea *clade. In the local test of alternative Ornstein-Uhlenbeck models (O-U models; the models and our 'local' and 'global' tests are described in the methods section), a shift in precipitation at the base of Clade 2 is supported with Bayes information criterion (BIC) weight = 0.876, and a shift in Emberger Index is supported at BIC weight = 0.842 (Table 2). A shift in Emberger's Index at the base of the *Xanthoparmelia *clade is similarly supported (BIC weight = 0.895). A shift in minimum temperature is very strongly supported at the base of the *Melanohalea *clade under both the local and global tests and whether measurement error is accounted for or not (BIC weight > 0.98; Tables 2 &3). These findings argue against a gradual shift in moisture regime in which the covariance among taxa is predicted by time since divergence from their most recent common ancestor. Rather, they support a scenario in which transitions in moisture regime are associated with cladogenetic events. In both the global and local tests of maximum temperature and altitude, the Brownian motion or single-equilibrium O-U model cannot be rejected (Tables 2 &3).

**Table 2:** Local test of support for Ornstein-Uhlenbeck (O-U) models of shifts in environmental conditions

Clade	sqrt (Altitude)	ln (Precipitation)	ln (Emberger Index)	minimum Temperature	maximum Temperature
*error = 0*					

1	0.337/0.662/<0.001	0.097/0.857/0.046	0.149/0.845/0.006	0.062/0.376/0.562	0.165/0.835/<0.001
2	0.107/0.891/0.001	0.876/0.117/0.006	0.842/0.157/0.001	0.141/0.344/0.514	0.161/0.839/<0.001
3	0.157/0.842/0.001	0.565/0.413/0.022	0.895/0.104/<0.001	0.041/0.385/0.574	0.105/0.895/<0.001
4	0.519/0.481/<0.001	0.090/0.864/0.047	0.099/0.894/0.006	0.040/0.385/0.575	0.308/0.692/<0.001
5	0.093/0.906/0.001	0.394/0.575/0.031	0.440/0.556/0.004	0.984/0.006/0.009	0.145/0.855/<0.001

*error = estimated squared standard error*					

1	0.021/0.010/0.970	0.043/0.361/0.596	0.037/0.206/0.757	0.048/0.010/0.942	<0.001/0.006/0.994
2	0.022/0.010/0.968	0.567/0.163/0.270	0.276/0.155/0.570	0.006/0.010/0.984	0.001/0.006/0.993
3	0.011/0.010/0.980	0.179/0.309/0.511	0.180/0.175/0.645	0.002/0.010/0.988	<0.001/0.006/0.994
4	0.134/0.009/0.857	0.038/0.363/0.600	0.023/0.209/0.768	0.002/0.010/0.988	0.002/0.006/0.992
5	0.001/0.010/0.989	0.148/0.321/0.531	0.075/0.198/0.727	1.000/<0.001/<0.001	0.001/0.006/0.993

For each variable, 7 models were evaluated: five dual-equilibrium O-U models corresponding to a single change in environment at the base of each of the five indicated clades; the single-equilibrium O-U model, in which all clades are modelled as occurring in a single environment; and the Brownian motion (constant variance) model. Values in each cell are Bayes information criterion (BIC) weights for: the dual-equilibrium O-U model/the single-equilibrium O-U model/the Brownian motion model. The first value is interpreted as the posterior probability that the origins of the clade were associated with a shift in environmental parameters. Measurement error for the lower portion of the table was estimated as indicated in the text.

**Table 3:** Global test of support for Ornstein-Uhlenbeck (O-U) models of shifts in environmental conditions

Clade	sqrt (Altitude)	ln (Precipitation)	ln (Emberger Index)	Min Temp	Max Temp
*error = 0*					

1	0.29978	0.60730	0.75952	0.12975	0.15825
2	0.16626	0.90511	0.90077	0.17785	0.17675
3	0.26579	0.52435	0.44646	0.76296	0.11206
4	0.52548	0.56360	0.76298	0.14681	0.30656
5	0.11212	0.49581	0.42547	0.98678	0.14469

*error = estimated squared standard error*					

1	0.31380	0.63645	0.80282	0.12743	0.00213
2	0.33394	0.89657	0.89803	0.11836	0.00327
3	0.62014	0.48266	0.40712	0.97213	0.00208
4	0.69243	0.58770	0.80586	0.13598	0.00518
5	0.17833	0.46473	0.39064	0.99029	0.00262

For each variable, 33 models were evaluated: 32 O-U models comprising all 2^5 possible models allowing or disallowing shifts in equilibrium value at the base of the 5 focus clades; and the Brownian motion (constant variance) model. Values in each cell are Bayes information criterion (BIC) weights summed over all models allowing a change at the node indicated. This estimates the posterior probability that there was a shift in environmental conditions at the base of the clade estimated integrating over all models evaluated. Measurement error for the lower portion of the table was estimated as indicated in the text.

Incorporating estimated measurement error decreases the relative support for the two-equilibrium O-U models in the local test (e.g., BIC weight = 0.567 and 0.276 for precipitation and Emberger's Index respectively for *Hypotrachyna *clade 2 when measurement error is accounted for; Table 2). In the global test, incorporating measurement error into analysis has little effect on the evidence for a change in moisture regime at the base of clade 2, which is supported at BIC weight = 0.897 to 0.905 either with or without measurement error (Table 3). The model-averaged estimate of *α *for Emberger's Index is 16.447 when measurement error is incorporated, 19.015 when it is not (Additional files 2, 3). This has the effect of almost erasing the phylogenetic effects in the data. When measurement error is disregarded (treated as zero), there is little support for a Brownian motion model for any characters except minimum temperature (Table 2). In the global analyses of precipitation and Emberger's Index, the 99% confidence interval excludes both the Brownian motion model and the single-moisture-regime O-U model (Table 3, Additional files 2, 3). When measurement error is ignored, there is concordance between the local and global tests.

## Discussion

Our extended sampling revealed phylogenetic estimates congruent with previous analyses [21,29] including several well-supported clades within parmelioid lichens. However, as in these studies, we failed to resolve with confidence the phylogenetic relationships between these strongly supported main clades. Further, additional sampling of the *Hypotrachyna *clade supported that it includes two major clades. In contrast to Blanco et al. [21], but in agreement with Divakar et al. [29], the sister-group relationship between the two *Hypotrachyna *clades is not strongly supported, and in the Bayesian analysis using Beast, the two clades do not form a monophyletic group. The lack of confidence in the phylogenetic relationships between the clades is consistent with the scenario of an adaptive radiation at the base of parmelioid clade. However, we cannot exclude the possibility of a lack of power of resolution in the gene partitions used in the analyses.

This study provides clear evidence for a general acceleration in rates of molecular evolution in the two *Hypotrachyna *clades in comparison with other clades in parmelioid lichens. Although this study presents the first statistical evidence for a significantly accelerated substitution rate in the *Hypotrachyna *clades, a prior study showed its occurrence without discussing this further [21]. The increased rates of nucleotide substitution seen could result from different factors, such as positive selection on coding regions of the ribosomal DNA, or represent a general acceleration of nucleotide substitutions [18]. Our analyses point to the latter as the most plausible cause.

Positive selection is the process favouring the retention of mutations in a population that are beneficial to the reproductive success of individuals [55]. Positive selection leading to increased fixation of nonsynonymous substitutions has been demonstrated for some parasites [56,57]. To examine the possibility of this process on coding regions of the ribosomal DNA, we examined the rate increase in a conserved part of the ITS regions (199 bp long) and compared it to the rate increase in the 5.8 S rDNA and nu LSU rDNA. If faster substitution rates were caused by differential selection, there should be no association between faster evolutionary rates and affiliation to the *Hypotrachyna *clades. However, we found higher rates in the *Hypotrachyna *clades in all three- gene partitions studied (data not shown). There were no significant differences between the rate increases between the gene partitions, indicating that positive selection on coding regions is not present.

Another possible cause of rate acceleration is an increase in mutation rate [58]. Our results indicate a general acceleration of nucleotide substitutions in the *Hypotrachyna *clade (see branch lengths, Figure 1). Causes for such an acceleration could be numerous, including shorter generation time [59], metabolic rate [60,61], lack of sexuality [62], or demographic factors [15,62].

A population bottleneck may result from a significant reduction of population size due to extinction of many genetic lineages. Since mutations have a higher probability of fixation in populations experiencing genetic bottlenecks, this may result in a long branch leading to a clade [6,63]. In fact, branches leading to *Hypotrachyna *clade 1 and 2 are long. However, this interpretation fails to account for the general rate acceleration in the *Hypotrachyna *clades. Populations of species in these two clades are not smaller and species do not have more restricted distributions than in other clades (PKD, unpubl. observations).

The loss of sexual reproduction has been identified as a cause of accelerated evolution in endosymbiotic bacteria [62,64]. Since the ratio of asexual/sexual species in the *Hypotrachyna *clades does not differ from other clades (0.29, 0.55 in *Hypotrachyna *clades and 0.27, 0.5, 1.25 in other clades), this explanation is also not likely.

Two intrinsic biological variables, generation time and metabolic rate, have been interpreted as causes of rate variations in other organisms [60,61,65-68]. Since rates of DNA damage are proportional to specific metabolic rate, species with higher metabolic rates should have higher substitution rates given there is a relationship between DNA damage and mutation rate [63]. Unfortunately, there are no ecophysiological measurements available for the lichen clades studied. Hence, we have no data on metabolic rates in these fungi. However, as mentioned above lichenized fungi are poikilohydric organisms and from studies in other groups of lichens, we can assume that species occurring in arid and semi-arid regions are dormant for large parts of their life [45,49,69]. For example, *Teloschistes capensis *(Teloschistaceae) occurs in the same habitats along the Namibian and north-western coast of South Africa as some taxa of the *Xanthoparmelia *clade studied here. This species has been shown to be physiologically active for only very few hours in the mornings after dew fall [48]. In contrast, species in moist habitats, such as rainforests were shown to be physiologically active throughout the day [51,52,70]. Growth measurements in lichens from arid and semi-arid regions revealed that these lichens grow very slowly (0.37 mm/y in *Caloplaca aurantia*, Teloschistaceae) [46] and hence reach an age of several hundred years, while some lichens in moist habitats, such as coastal forests of California can increase their length over 35% per year [71] and have a much shorter generation time. The slow rates of mutations in *Xanthoparmelia *clade may be related with a strategy adapted to a low metabolic activity due to long dormancy periods in dry conditions. Again, we have no data from the parmelioid lichens studied here and point out that ecophysiological studies need to be extended to these lichens to confirm their general importance.

Given our knowledge of the differences in growth rate and generation time for these poikilohydric lichens in moist vs. arid regions, we hypothesized at the outset of this study that a shift in moisture regime might explain the increased nucleotide substitution rate in *Hypotrachyna *clades 1 and 2. This might be due either to decreased generation time or increased mutation rate in environments with generally higher precipitation than in the other three clades. The support for O-U models that specify shifts in moisture regime (Emberger's Index and precipitation) at the base of *Hypotrachyna *clade 2 and minimum temperature at the base of clade 5 (*Melanohalea*), combined with weak support for the Brownian motion model and single-environment Ornstein-Uhlenbeck model for these variables, suggests that these clades originated by colonization of a novel environment, followed by diversification within those environments. The extreme phylogeny-effacing effects of the high inferred rate of evolution toward equilibrium Emberger index values particularly suggests that shifts in clade-specific moisture regimes govern the evolution of habitat preferences in these lichens. There is support for a change in moisture regime at the base of *Hypotrachyna *clade 1 (BIC weight = 0.760 without measurement error, 0.803 with error; Table 3), which is comparable to the support for a change at the base of *Hypotrachyna *clade 2. These two clades have an accelerated substitution rate. However, the support for a change is also approximately the same for the *Parmotrema *clade (BIC weight = 0.763 without error, 0.806 with error), which does not exhibit elevated substitution rate.

The apparently weak link between moisture regime and substitution rate in this study may be due to a lack of causal relationship. However, it might also be due to an alternative adaptative strategy of the *Parmotrema *clade. Species in this clade are especially abundant in insular regions [72] with oceanic climate. Although no ecophysiological data on parmelioid lichens are available, other lichens occurring in similar moisture conditions, were shown to have metabolic (and growth) rates depending on a combination of light intensity and temperature [73]. Most species in the *Parmotrema *clade may have slow metabolic rates due to ecological conditions related with latitude. In fact, *Parmotrema *clade species are subtropical or temperate [28,74], living in regions where, comparing to tropics, light intensity is lower. Wright et al. [75] showed that evolutionary rates of tropical species are higher than those of subtropical taxa. However, low sample size (inadequate number of taxa to accurately estimate the posterior probability support for environmental shifts associated with shifts in substitution rate) or inadequate characterization of the moisture environment cannot be discarded. Additionally, in our observation (T.L. and A.C.), the moisture available to the lichens in this study is strongly dominated by fog, which is not directly accounted for by either precipitation or Emberger's Index (though it is likely to be correlated). In species occurring in areas with extensive fogs, lichens were shown to be able to use this as the main source of water taken up from water vapour [36,76]. Future work in the ecology of this group of organisms should focus on characterizing moisture regime more accurately.

## Conclusion

Our extended taxon sampling confirmed phylogenetic relationships revealed in previous studies. Here we show significant accelerated evolutionary rates in clades of parmelioid lichens that occur in tropical and oceanic habitats as opposed to those in arid habitats. Comparison of alternative Ornstein-Uhlenbeck models gave moderate support for a shift in moisture regime at the base of one tropical clade and a clade occurring in semi-arid regions and a shift in minimum temperature at the base of a boreal-temperate clade. This finding that cladogenesis may be associated with shifts in environment suggests adaptive radiation as a mechanism of speciation in Parmelioid lichens, a hypothesis that bears testing using finer-grained ecological data.

## Methods

### Taxon Sampling

Data matrices of 128 parmelioid lichens and three outgroup taxa were assembled using sequences of nuclear LSU, ITS, and mitochondrial SSU rDNA sequences. Specimens and sequences used for the molecular analyses are compiled in Additional file 4. The data set includes 11 sequences downloaded from GenBank, 343 from previous publications by us [21,23,29,32,74,77], and 39 sequences newly generated for this study. We used three species of the genus *Usnea *as the out-group since this genus has been shown to be closely related to parmelioid lichens in a previous study [23].

### Molecular methods

Small samples prepared from freshly collected and frozen specimens were ground with sterile plastic pestles. Total genomic DNA was extracted using the DNeasy Plant Mini Kit (Qiagen, Hilden) according to the manufacturer's instructions but with slight modifications as described elsewhere [22]. Fungal nu LSU rDNA was amplified using the primers nu-LSU-0155-5' [78], LR0R (Vilgalys unpublished, http://www.botany.duke.edu/fungi/mycolab), LR5, LR6 and LR7 [79] and mt SSU rDNA using the primers mrSSU1, mrSSU3R [80], MSU1 and MSU7 [81].

Amplifications were performed in 50 μl volumes containing 5–25 ng of DNA and a reaction mixture of 5 μl 10× DNA polymerase buffer (Biotools) (containing MgCl_2 _2 mM, 10 mM Tris-HCl, pH 8.0, 50 mM KCl, 1 mM EDTA, 0.1% Triton X-100), 1 μl of deoxinucleotide triphosphate (dNTPs), containing 10 mM of each base, 2.5 μl of each primer (10 μM) and 1.25 μl of DNA polymerase (1 U/μl). The amplifications were carried out in an automatic thermocycler (Techne Progene) and performed using the same programs as described in [82]. The PCR products were then cleaned using the Bioclean Columns kit (Biotools, Madrid) according to the manufacturer's instructions. The cleaned PCR products were sequenced using the same primers as used for PCR amplification. The ABI Prism™ Dye Terminator Cycle Sequencing Ready reaction kit (Applied Biosystems, Foster City) was used and the following settings were applied: denaturation for 3 min at 94°C, 25 cycles at 96°C for 10 sec, 50°C for 5 sec, and 60°C for 4 min. Sequencing reactions were electrophoresed on a 3730 DNA analyzer (Applied Biosystems, Foster City). Sequence fragments obtained were assembled with SeqMan 4.03 (DNAStar, Madison) and manually adjusted.

### Sequence alignment

The mitochondrial and the ITS data sets contain sequence portions that are highly variable. Standard multiple alignment programs, such as Clustal [83] become less reliable when sequences show a high degree of divergence. Therefore we employed an alignment procedure that uses a linear Hidden Markov Model (HMM) as implemented in the software SAM (Sequence Alignment and Modelling system) [84] for separate alignments of the three data sets. Regions that were not aligned with statistical confidence were excluded from the phylogenetic analysis.

### Phylogenetic analyses

The alignments were analysed by maximum parsimony (MP) and a Bayesian approach (B/MCMC) [85,86]. To test for potential conflict, parsimony bootstrap analyses were performed on each individual data set, and 75% bootstrap consensus trees were examined for conflict [20].

Maximum parsimony analyses were performed using the program PAUP* [87]. Heuristic searches with 1000 random taxon addition replicates were conducted with TBR branch swapping and MulTrees option in effect, equally weighted characters and gaps treated as missing data. Bootstrapping [88] was performed based on 2000 replicates with random sequence additions.

The B/MCMC analyses were conducted using the MrBayes 3.1.1 program [89]. The analyses were performed assuming the general time reversible model of nucleotide substitution [90], including estimation of invariant sites and assuming a discrete gamma distribution with six rate categories (GTR+I+G). The data set was portioned into the three parts (ITS, nu LSU, mt SSU) and each partition was allowed to have its own parameters [91]. No molecular clock was assumed. A run with 3,000,000 generations starting with a random tree and employing 12 simultaneous chains was executed. Every 100^th ^tree was saved into a file. The first 200,000 generations (i.e. the first 2000 trees) were deleted as the "burn in" of the chain. We plotted the log-likelihood scores of sample points against generation time using TRACER 1.0 http://evolve.zoo.ox.ac.uk/software.html?id=tracer to ensure that stationarity was achieved after the first 200,000 generations by checking whether the log-likelihood values of the sample points reached a stable equilibrium value [89]. Of the remaining 56,000 trees (28,000 from each of the parallel runs) a majority rule consensus tree with average branch lengths was calculated using the sumt option of MrBayes. Posterior probabilities were obtained for each clade. Clades with bootstrap support equal or above 75% under MP and posterior probabilities ≥ 0.95 were considered as strongly supported. Phylogenetic trees were visualized using the program Treeview [92].

### Sequence Significance Test for the Presence of Rate Differences

The program Baseml (part of the PAML 3.14 b package; [93] was used for significance tests for the presence of rate differences, assuming a GTR+G model. The resulting likelihoods were compared using likelihood ratio tests [94]. Two types of analyses were performed and the results of those are listed in Table 1. In a first set of analyses, two models were compared to determine whether observed branch length differences were the result of a significant departure from rate constancy. The null model assumed a molecular clock, while in the alternative model each branch was allowed its own unique rate of molecular evolution. These two models were contrasted for each gene partition separately (ITS, mtSSU, nuLSU) across the whole ingroup of parmelioid lichens, as well as across five selected, well-supported major clades within parmelioid lichens, which are *Hypotrachyna *1, *Hypotrachyna *2, *Melanohalea*, *Parmotrema *and *Xanthoparmelia *[21,29] to determine rate differences within these clades. In a second analysis, several tests were performed to determine whether significant departure from rate homogeneity were present between the five selected clades. For these analyses, a pruned tree comprising of the five selected clades of parmelioid lichens was used for comparisons of a null and alternative model. The five clades selected included the two clades (*Hypotrachyna *clade 1 and 2) with evidently long branches and the *Xanthoparmelia *clade that has comparably short branches. Comparisons were made between two-rate models in which two selected clades had the same rate but a third clade had a different rate versus a three-rate model in which all three included clades had different rates. These analyses were made on the concatenated data set, and in total 30 analyses were performed.

### Phylogenetic Comparative Analyses

Estimates of five environmental variables – Emberger's Index [95], altitude, precipitation, minimum temperature and maximum temperature – were obtained for one to three specimens of each of 97 species from three online databanks (http://www.ucm.es/info/cif/data/indexcsp.htm, http://climexp.knmi.nl/start.cgi?someone@somewhere, and http://www.worldclimate.com/) and are available in the additional file 4 and 5. The Emberger's index including a combination of simple climate parameters such as the mean of minimal and maximal annual temperature and precipitation was calculated by the formula Q = 100 × P/Tm_max_^2 ^- Tm_min_^2^, where P is average annual precipitation, Tm_max _is average maximum temperature of warmest month, Tm_min _is average minimum temperature of coldest month.

Environmental variables were analyzed in a phylogenetic generalized least squares framework under alternative Ornstein-Uhlenbeck (O-U) models [53,96]. The O-U process is essentially a Brownian motion model with a pull toward an equilibrium that may be environment-specific or clade specific. The O-U process has been referred to as a "rubber-band" process [97], because it models random, stochastic processes as well as deterministic evolutionary forces that pull character values toward a central optimum or equilibrium value. Under this model, the parameter *α *estimates the rate at which the variable being analyzed reaches an equilibrium, and the parameter *θ*_*i *_estimates the equilibrium value of that variable along branches in group *i*. Analyses were conducted allowing θ to change at the base of each of five strongly supported clades (Figure 1). This models the situation in which environmental conditions shift at the base of a clade, such that the radiation of that clade takes place within a potentially novel set of environmental conditions. Bayes information criterion (BIC) weights were used to assess the support for the hypothesis that each clade radiated under a novel set of conditions relative to each of the five environmental variables. BIC weights are interpreted as Bayesian posterior model probabilities assuming equal prior model probabilities [98]. Strong relative support for a single-equilibrium O-U model or a Brownian motion model (constant variance with respect to phylogeny) indicates that the data do not support a shift in environmental conditions attending cladogenetic events. Emberger's Index and precipitation were log-transformed and altitude was square-root transformed prior to analysis. Transformations had little effect on the conclusions of this paper but improved model fit in trial analyses by 400 to 600 log-likelihood units.

We evaluated the support for alternative hypotheses both locally and globally. In local tests, we compared the fit of three models for each dataset: a two-equilibrium O-U model that allows a change in environment at the base of a single clade; the single-equilibrium O-U model, in which the entire tree comprises a single environmental regime; and the Brownian motion model. In global tests, we compared the fit of the Brownian motion model plus all 5^2 = 32 possible O-U models, allowing changes at all permutations of the five focus clades, and summed BIC weights over these clades to estimate the global support for a change in environment attending the origins of each clade. This approach is analogous to model-averaging and has proven useful as a means of estimating the posterior probability of change occurring along a given phylogenetic branch [99]. Effects of the O-U *α *parameter on phylogenetic signal were visualized using the Geiger package [100] in R 2.6.0 (R Development Core Team 2007).

Analyses were conducted on an ultrametric tree estimated using the lognormal relaxed clock model [101] implemented in an MCMC framework in BEAST v1.4.5 [102], using the Bayesian consensus as the start tree and a GTR+I+G model of nucleotide substitution, with a total run of 20,000,000 generations. Measurement error was incorporated into analyses by adding an estimate of the squared standard error for each environmental variable to the diagonal of the variance-covariance matrix utilized in generalized least squares computations [100]. Because the small sample sizes (*N *= 1 to 3 samples per species) increase uncertainty in the estimates of measurement error, the squared standard error was estimated for each environmental variable as the mean variance over all species represented by *N *= 3 samples, divided by the sample size for each species separately. With such small samples, measurement error in this study represented a substantial component of the total variance in the tree, and analyses incorporating measurement error were consequently compared with analyses in which measurement error was set at zero.

## Authors' contributions

HTL carried out the phylogenetic and rate analyses and drafted the manuscript. ALH carried out the phylogenetic comparative analyses and helped draft the manuscript. OB and PKD contributed DNA sequences and helped draft the manuscript. AC initiated and coordinated the work, helped draft the manuscript, and submitted the manuscript.

## Supplementary Material

Additional file 1 Table S1: Likelihood ratio test comparisons performed within and between clades. Click here for file

Additional file 2 Table S2: Models evaluated in the global test of Emberger's Index, not incorporating measurement error. Click here for file

Additional file 3 Table S3 – Models evaluated in the global test of Emberger's Index, incorporating the estimated squared standard error of the mean as measurement error. Click here for file

Additional file 4 Table S4 – Ecological parameters of specimens. Click here for file

Additional file 5 S5 – Species and specimens used in the current study with Genbank accession numbers. includes data of sequences used in the analyses and their specimens. Click here for file
