# Supplementary material for: Accelerated evolutionary rates in tropical and oceanic parmelioid lichens (Ascomycota)
Source: BMC Evol Biol. 2008 Sep 22;8:257. doi: 10.1186/1471-2148-8-257 (PMC2564941; doi:10.1186/1471-2148-8-257)
Supplement: Additional file 1 — Table S1: Likelihood ratio test comparisons performed within and between clades. [file 1471-2148-8-257-S1.doc]

| **Comparison of models using BASEML** | **Data set** | **Taxa** | **df** | **ln L (null)** | **ln L (alt.)** | **LRT** | ***P*** | **Bonferroni-corrected *P*** |
| --- | --- | --- | --- | --- | --- | --- | --- | --- |
| **1. Rate differences within clades** |  |  |  |  |  |  |  | **(*N* = 18 tests)** |
| Global molecular clock vs. no molecular clock | ITS | Entire ingroup | 128 | -8812.374 | -8663.349 | 298.05 | < 0.0001 | < 0.0001 |
| Global molecular clock vs. no molecular clock | ITS | Hypotrachyna clade 1 | 28 | -2355.028 | -2292.644 | 124.768 | < 0.0001 | < 0.0001 |
| Global molecular clock vs. no molecular clock | ITS | Hypotrachyna clade 2 | 17 | -2123.201 | -2075.554 | 95.294 | < 0.0001 | < 0.0001 |
| Global molecular clock vs. no molecular clock | ITS | Melanohalea clade | 9 | -1008.532 | -998.407 | 20.25 | 0.0164 | 0.2958 |
| Global molecular clock vs. no molecular clock | ITS | Parmotrema clade | 15 | -1304.398 | -1288.708 | 31.22 | 0.0082 | 0.1477 |
| Global molecular clock vs. no molecular clock | ITS | Xanthoparmelia clade | 29 | -1666.617 | -1651.409 | 30.416 | 0.3935 | 1.0000 |
| Global molecular clock vs. no molecular clock | mt SSU | Entire ingroup | 128 | -8033.163 | -7775.533 | 515.26 | < 0.0001 | < 0.0001 |
| Global molecular clock vs. no molecular clock | mt SSU | Hypotrachyna clade 2 | 17 | -2396.65 | -2307.077 | 179.146 | < 0.0001 | < 0.0001 |
| Global molecular clock vs. no molecular clock | mt SSU | Melanohalea clade | 9 | -1447.302 | -1420.203 | 54.198 | < 0.0001 | < 0.0001 |
| Global molecular clock vs. no molecular clock | mtSSU | Parmotrema clade | 15 | -1664.023 | -1649.042 | 29.962 | 0.0121 | 0.2171 |
| Global molecular clock vs. no molecular clock | mtSSU | Xanthoparmelia clade | 29 | -1635.515 | -1586.964 | 97.102 | < 0.0001 | < 0.0001 |
| Global molecular clock vs. no molecular clock | mtSSU | Hypotrachyna clade 1 | 28 | -2522.266 | -2452.137 | 140.258 | < 0.0001 | < 0.0001 |
| Global molecular clock vs. no molecular clock | nu LSU | Entire ingroup | 128 | -7303.28 | -7135.955 | 334.65 | < 0.0001 | < 0.0001 |
| Global molecular clock vs. no molecular clock | nu LSU | Hypotrachyna clade 1 | 28 | -2354.872 | -2296.62 | 116.504 | < 0.0001 | < 0.0001 |
| Global molecular clock vs. no molecular clock | nu LSU | Hypotrachyna clade 2 | 17 | -2425.871 | -2372.862 | 106.018 | < 0.0001 | < 0.0001 |
| Global molecular clock vs. no molecular clock | nu LSU | Melanohalea clade | 9 | -1627.352 | -1600.058 | 54.588 | < 0.0001 | < 0.0001 |
| Global molecular clock vs. no molecular clock | nu LSU | Parmotrema clade | 15 | -1728.093 | -1710.286 | 35.614 | 0.0020 | 0.0362 |
| Global molecular clock vs. no molecular clock | nu LSU | Xanthoparmelia clade | 29 | -1635.545 | -1586.964 | 97.102 | < 0.0001 | < 0.0001 |
| **1. Rate differences between clades** |  |  |  |  |  |  |  | **(*N* = 30 tests)** |
| Two rate model (Hypotrachyna clade 1 = Hypotrachyna clade 2 # Melanohalea clade) vs. three-rate model | 3-gene data set | Five clades | 1 | -19778.61 | -19765.643 | 25.934 | < 0.0001 | < 0.0001 |
| Two rate model (Hypotrachyna clade 1 = Melanohalea clade # Hypotrachyna clade 2) vs. three-rate model | 3-gene data set | Five clades | 1 | -19780.06 | -19765.643 | 28.834 | < 0.0001 | < 0.0001 |
| Two rate model (Hypotrachyna clade 2 = Melanohalea clade # Hypotrachyna clade 1) vs. three-rate model | 3-gene data set | Five clades | 1 | -19828.03 | -19765.643 | 124.774 | < 0.0001 | < 0.0001 |
| Two rate model (Hypotrachyna clade 1 = Hypotrachyna clade 2 # Parmotrema clade) vs. three-rate model | 3-gene data set | Five clades | 1 | -19905.51 | -19892.579 | 25.862 | < 0.0001 | < 0.0001 |
| Two rate model (Hypotrachyna clade 1 = Parmotrema clade # Hypotrachyna clade 2) vs. three-rate model | 3-gene data set | Five clades | 1 | -19900.21 | -19892.579 | 15.262 | < 0.0001 | 0.0028 |
| Two rate model (Hypotrachyna clade 2 = Parmotrema clade # Hypotrachyna clade 1) vs. three-rate model | 3-gene data set | Five clades | 1 | -19930.96 | -19892.579 | 76.766 | < 0.0001 | < 0.0001 |
| Two rate model (Hypotrachyna clade 1 = Hypotrachyna clade 2 # Xanthoparmelia clade) v.s three rate model | 3-gene data set | Five clades | 1 | -19689.13 | -19685.592 | 7.068 | 0.0078 | 0.2354 |
| Two rate model (Hypotrachyna clade 1 = Xanthoparmelia clade # Hypotrachyna clade 2) v.s three rate model | 3-gene data set | Five clades | 1 | -19701.21 | -19685.592 | 31.244 | < 0.0001 | < 0.0001 |
| Two rate model (Hypotrachyna clade 2 = Xanthoparmelia clade # Hypotrachyna clade 1) v.s three rate model | 3-gene data set | Five clades | 1 | -19753.37 | -19685.592 | 135.554 | < 0.0001 | < 0.0001 |
| Two rate model (Hypotrachyna clade 1 = Melanohalea clade # Parmotrema clade) vs. three-rate model | 3-gene data set | Five clades | 1 | -19814.22 | -19777.106 | 74.23 | < 0.0001 | < 0.0001 |
| Two rate model (Hypotrachyna clade 1 = Parmotrema clade # Melanohalea clade) vs. three-rate model | 3-gene data set | Five clades | 1 | -19805.13 | -19777.106 | 56.042 | < 0.0001 | < 0.0001 |
| Two rate model (Melanohalea clade = Parmotrema clade # Hypotrachyna clade 1) vs. three-rate model | 3-gene data set | Five clades | 1 | -19778.61 | -19777.106 | 3 | 0.0833 | 1.0000 |
| Two rate model (Hypotrachyna clade 1 = Melanohalea clade # Xanthoparmelia clade) vs. three-rate model | 3-gene data set | Five clades | 1 | -19772.19 | -19762.709 | 18.962 | < 0.0001 | 0.0004 |
| Two rate model (Hypotrachyna clade 1 = Xanthoparmelia clade # Melanohalea clade) vs. three-rate model | 3-gene data set | Five clades | 1 | -19772.83 | -19762.709 | 20.234 | < 0.0001 | 0.0002 |
| Two rate model (Melanohalea clade = Xanthoparmelia clade # Hypotrachyna clade 1) vs. three-rate model | 3-gene data set | Five clades | 1 | -19764.09 | -19762.709 | 2.762 | 0.0965 | 1.0000 |
| Two rate model (Hypotrachyna clade = Parmotrema clade # Xanthopamelia clade) vs. three-rate model | 3-gene data set | Five clades | 1 | -19729.13 | -19695.895 | 66.462 | < 0.0001 | < 0.0001 |
| Two rate model (Hypotrachyna clade = Xanthopamelia clade # Parmotrema clade) vs. three-rate model | 3-gene data set | Five clades | 1 | -19738.48 | -19695.895 | 85.16 | < 0.0001 | < 0.0001 |
| Two rate model (Parmotrema clade = Xanthopamelia clade # Hypotrachyna clade) vs. three-rate model | 3-gene data set | Five clades | 1 | -19699.98 | -19695.895 | 8.166 | 0.0043 | 0.1280 |
| Two rate model (Hypotrachyna clade 2 = Melanohalea clade # Parmotrema clade) vs. three-rate model | 3-gene data set | Five clades | 1 | -19818.65 | -19771.904 | 93.494 | < 0.0001 | < 0.0001 |
| Two rate model (Hypotrachyna clade 2 = Parmotrema clade # Melanohalea clade) vs. three-rate model | 3-gene data set | Five clades | 1 | -19805.13 | -19771.904 | 66.446 | < 0.0001 | < 0.0001 |
| Two rate model (Melanohalea clade = Parmotrema clade # Hypotrachyna clade 2) vs. three-rate model | 3-gene data set | Five clades | 1 | -19780.06 | -19771.904 | 16.302 | < 0.0001 | 0.0016 |
| Two rate model (Hypotrachyna clade 2 = Melanohalea clade # Xanthoparmelia clade) vs. three-rate model | 3-gene data set | Five clades | 1 | -19764.09 | -19736.225 | 55.728 | < 0.0001 | < 0.0001 |
| Two rate model (Hypotrachyna clade 2 = Xanthoparmelia clade # Melanohalea clade) vs. three-rate model | 3-gene data set | Five clades | 1 | -19758.77 | -19736.225 | 45.096 | < 0.0001 | < 0.0001 |
| Two rate model (Melanohalea clade = Xanthoparmelia clade # Hypotrachyna clade 2 ) vs. three-rate model | 3-gene data set | Five clades | 1 | -19739.48 | -19736.225 | 6.504 | 0.0108 | 0.3229 |
| Two rate model (Hypotrachyna clade 2 = Parmotrema clade # Xanthoparmelia clade) vs. three-rate model | 3-gene data set | Five clades | 1 | -19724.32 | -19690.533 | 67.568 | < 0.0001 | < 0.0001 |
| Two rate model (Hypotrachyna clade 2 = Xanthoparmelia clade # Parmotrema clade) vs. three-rate model | 3-gene data set | Five clades | 1 | -19742.14 | -19690.533 | 103.222 | < 0.0001 | < 0.0001 |
| Two rate model (Parmotrema clade = Xanthoparmelia clade # Hypotrachyna clade 2) vs. three-rate model | 3-gene data set | Five clades | 1 | -19701.21 | -19690.533 | 21.362 | < 0.0001 | 0.0001 |
| Two rate model (Melanohalea clade = Parmotrema clade # Xanthoparmelia clade) vs. three-rate rates | 3-gene data set | Five clades | 1 | -19707.78 | -19707.716 | 0.122 | 0.7269 | 1.0000 |
| Two rate model (Melanohalea clade = Xanthoparmelia clade # Parmotrema clade) vs. three-rate rates | 3-gene data set | Five clades | 1 | -19708.09 | -19707.716 | 0.746 | 0.3877 | 1.0000 |
| Two rate model (Parmotrema clade = Xanthoparmelia clade # Melanohalea clade) vs. three-rate rates | 3-gene data set | Five clades | 1 | -19707.85 | -19707.716 | 0.276 | 0.5993 | 1.0000 |
