# Supplementary material for: Accelerated evolutionary rates in tropical and oceanic parmelioid lichens (Ascomycota)
Source: BMC Evol Biol. 2008 Sep 22;8:257. doi: 10.1186/1471-2148-8-257 (PMC2564941; doi:10.1186/1471-2148-8-257)
Supplement: Additional file 2 — Table S2: Models evaluated in the global test of Emberger's Index, not incorporating measurement error. [file 1471-2148-8-257-S2.doc]

| **Model** | **Clade 1** | **Clade 2** | **Clade 3** | **Clade 4** | **Clade 5** | **log-likelihood** | ***K*** | **BIC** | **BIC weight** | **cumulative BIC weight** | **α** | **σ** | **θ-ancestral** | **θ-root** | **θ-clade 1** | **θ-clade 2** | **θ-clade 3** | **θ-clade 4** | **θ-clade 5** |
| --- | --- | --- | --- | --- | --- | --- | --- | --- | --- | --- | --- | --- | --- | --- | --- | --- | --- | --- | --- |
| 11 | + | + |  | + |  | -60.76 | 7 | 153.5 | 0.4898 | 0.490 | 19.618 | 2.898 | 5.99E-08 | 4.252 | 5.151 | 5.526 | -- | 4.890 | -- |
| 22 |  | + | + |  | + | -62.01 | 7 | 156.0 | 0.1408 | 0.631 | 18.043 | 2.824 | 2.79E-07 | 5.061 | -- | 5.526 | 4.216 | -- | 4.335 |
| 30 |  | + | + | + | + | -60.58 | 8 | 157.8 | 0.0597 | 0.690 | 19.566 | 2.889 | 7.68E-08 | 5.151 | -- | 5.526 | 4.219 | 4.889 | 4.337 |
| 15 | + | + | + | + |  | -60.58 | 8 | 157.8 | 0.0597 | 0.750 | 19.566 | 2.889 | 7.68E-08 | 4.337 | 5.151 | 5.526 | 4.220 | 4.890 | -- |
| 23 | + | + | + |  | + | -60.58 | 8 | 157.8 | 0.0597 | 0.810 | 19.566 | 2.889 | 7.68E-08 | 4.889 | 5.151 | 5.526 | 4.219 | -- | 4.337 |
| 27 | + | + |  | + | + | -60.58 | 8 | 157.8 | 0.0597 | 0.869 | 19.566 | 2.889 | 7.68E-08 | 4.220 | 5.151 | 5.526 | -- | 4.890 | 4.337 |
| 29 | + |  | + | + | + | -60.58 | 8 | 157.8 | 0.0597 | 0.929 | 19.566 | 2.889 | 7.67E-08 | 5.526 | 5.151 | -- | 4.218 | 4.889 | 4.336 |
| 28 |  |  | + | + | + | -63.62 | 7 | 159.3 | 0.0282 | 0.957 | 14.907 | 2.631 | 6.28E-06 | 5.293 | -- | -- | 4.205 | 4.887 | 4.330 |
| 7 | + | + | + |  |  | -63.97 | 7 | 160.0 | 0.0198 | 0.977 | 15.675 | 2.702 | 3.05E-06 | 4.688 | 5.153 | 5.527 | 4.211 | -- | -- |
| 20 |  |  | + |  | + | -66.93 | 6 | 161.3 | 0.0101 | 0.987 | 11.366 | 2.414 | 1.59E-04 | 5.197 | -- | -- | 4.180 | -- | 4.314 |
| 31 | + | + | + | + | + | -60.58 | 9 | 162.3 | 6.06E-03 | 0.993 | 19.566 | 2.889 | 7.68E-08 | 0.013 | 5.151 | 5.526 | 4.226 | 4.894 | 4.339 |
| 3 | + | + |  |  |  | -68.01 | 6 | 163.5 | 3.44E-03 | 0.997 | 7.986 | 2.101 | 5.16E-03 | 4.471 | 5.172 | 5.523 | -- | -- | -- |
| 6 |  | + | + |  |  | -68.87 | 6 | 165.2 | 1.46E-03 | 0.998 | 11.021 | 2.429 | 2.40E-04 | 4.943 | -- | 5.526 | 4.183 | -- | -- |
| 21 | + |  | + |  | + | -66.79 | 7 | 165.6 | 1.18E-03 | 0.999 | 11.534 | 2.426 | 1.85E-04 | 5.232 | 5.156 | -- | 4.181 | -- | 4.315 |
| 19 | + | + |  |  | + | -67.64 | 7 | 167.3 | 5.06E-04 | 1.000 | 7.955 | 2.090 | 6.85E-03 | 4.515 | 5.172 | 5.523 | -- | -- | 4.300 |
| 14 |  | + | + | + |  | -68.77 | 7 | 169.6 | 1.64E-04 | 1.000 | 11.104 | 2.435 | 2.95E-04 | 4.966 | -- | 5.526 | 4.183 | 4.887 | -- |
| 4 |  |  | + |  |  | -74.33 | 5 | 171.5 | 6.08E-05 | 1.000 | 5.733 | 1.969 | 2.98E-02 | 5.118 | -- | -- | 4.048 | -- | -- |
| 2 |  | + |  |  |  | -74.80 | 5 | 172.5 | 3.81E-05 | 1.000 | 4.120 | 1.763 | 0.173 | 4.847 | -- | 5.602 | -- | -- | -- |
| 18 |  | + |  |  | + | -73.14 | 6 | 173.7 | 2.02E-05 | 1.000 | 4.568 | 1.793 | 0.154 | 4.904 | -- | 5.567 | -- | -- | 4.220 |
| 12 |  |  | + | + |  | -73.56 | 6 | 174.6 | 1.34E-05 | 1.000 | 6.380 | 2.034 | 0.024 | 5.162 | -- | -- | 4.068 | 4.856 | -- |
| 5 | + |  | + |  |  | -74.14 | 6 | 175.7 | 7.46E-06 | 1.000 | 5.907 | 1.987 | 0.039 | 5.070 | 5.187 | -- | 4.062 | -- | -- |
| 0 |  |  |  |  |  | -78.77 | 4 | 175.8 | 7.07E-06 | 1.000 | 2.889 | 1.652 | 0.311 | 5.277 | -- | -- | -- | -- | -- |
| 16 |  |  |  |  | + | -76.72 | 5 | 176.3 | 5.59E-06 | 1.000 | 3.433 | 1.698 | 0.315 | 5.233 | -- | -- | -- | -- | 4.189 |
| 10 |  | + |  | + |  | -74.72 | 6 | 176.9 | 4.19E-06 | 1.000 | 4.169 | 1.768 | 0.242 | 4.969 | -- | 5.597 | -- | 4.817 |  |
| 26 |  | + |  | + | + | -73.14 | 7 | 178.3 | 2.06E-06 | 1.000 | 4.574 | 1.794 | 0.204 | 4.920 | -- | 5.567 | -- | 4.899 | 4.220 |
| 13 | + |  | + | + |  | -73.54 | 7 | 179.1 | 1.38E-06 | 1.000 | 6.374 | 2.033 | 0.033 | 5.177 | 5.148 | -- | 4.070 | 4.858 | -- |
| 1 | + |  |  |  |  | -78.22 | 5 | 179.3 | 1.24E-06 | 1.000 | 3.055 | 1.668 | 0.525 | 5.492 | 5.127 | -- | -- | -- | -- |
| 8 |  |  |  | + |  | -78.68 | 5 | 180.2 | 7.85E-07 | 1.000 | 2.899 | 1.652 | 0.601 | 5.282 | -- | -- | -- | 5.032 | -- |
| 24 |  |  |  | + | + | -76.43 | 6 | 180.3 | 7.59E-07 | 1.000 | 3.469 | 1.699 | 0.461 | 5.264 | -- | -- | -- | 4.910 | 4.172 |
| 17 | + |  |  |  | + | -76.47 | 6 | 180.4 | 7.26E-07 | 1.000 | 3.540 | 1.710 | 0.440 | 5.357 | 5.147 | -- | -- | -- | 4.200 |
| 9 | + |  |  | + |  | -78.21 | 6 | 183.9 | 1.28E-07 | 1.000 | 3.049 | 1.667 | 0.779 | 5.479 | 5.129 | -- | -- | 5.041 | -- |
| 25 | + |  |  | + | + | -76.31 | 7 | 184.6 | 8.68E-08 | 1.000 | 3.533 | 1.706 | 0.591 | 5.347 | 5.194 | -- | -- | 4.921 | 4.183 |
| brown |  |  |  |  |  | -88.29 | 2 | 185.7 | 5.06E-08 | 1.000 | -- | 1.315 | 5.049 | -- | -- | -- | -- | -- | -- |
|  | 0.7595 | 0.9008 | 0.4465 | 0.7630 | 0.4255 |  |  |  |  |  | 19.015 | 2.864 | 4.35E-05 | 4.562 | 5.152 | 5.525 | 4.215 | 4.890 | 4.336 |
|  |  |  |  |  |  |  |  |  |  |  |  |  | [1.00] | [95.77] | [172.77] | [250.95] | [67.72] | [132.96] | [76.43] |
