# Supplementary material for: Accelerated evolutionary rates in tropical and oceanic parmelioid lichens (Ascomycota)
Source: BMC Evol Biol. 2008 Sep 22;8:257. doi: 10.1186/1471-2148-8-257 (PMC2564941; doi:10.1186/1471-2148-8-257)
Supplement: Additional file 3 — Table S3 – Models evaluated in the global test of Emberger's Index, incorporating the estimated squared standard error of the mean as measurement error. [file 1471-2148-8-257-S3.doc]

| **Model** | **Clade 1** | **Clade 2** | **Clade 3** | **Clade 4** | **Clade 5** | **log-likelihood** | ***K*** | **BIC** | **BIC weight** | **cumulative BIC weight** | **α** | **σ** | **θ-ancestral** | **θ-root** | **θ-clade 1** | **θ-clade 2** | **θ-clade 3** | **θ-clade 4** | **θ-clade 5** |
| --- | --- | --- | --- | --- | --- | --- | --- | --- | --- | --- | --- | --- | --- | --- | --- | --- | --- | --- | --- |
| 11 | + | + |  | + |  | -62.126 | 7 | 156.275 | 0.520 | 0.520 | 17.544 | 1.400 | 4.77E-07 | 4.257 | 5.179 | 5.540 | -- | 4.887 | -- |
| 22 |  | + | + |  | + | -63.829 | 7 | 159.682 | 0.095 | 0.614 | 12.869 | 1.364 | 4.94E-05 | 5.072 | -- | 5.541 | 4.214 | -- | 4.330 |
| 30 |  | + | + | + | + | -61.955 | 8 | 160.507 | 0.063 | 0.677 | 17.200 | 1.394 | 8.19E-07 | 5.179 | -- | 5.540 | 4.228 | 4.885 | 4.337 |
| 27 | + | + |  | + | + | -61.955 | 8 | 160.507 | 0.063 | 0.739 | 17.200 | 1.394 | 8.19E-07 | 4.231 | 5.179 | 5.540 | -- | 4.887 | 4.338 |
| 15 | + | + | + | + |  | -61.955 | 8 | 160.507 | 0.063 | 0.802 | 17.200 | 1.394 | 8.19E-07 | 4.338 | 5.179 | 5.540 | 4.230 | 4.887 | -- |
| 23 | + | + | + |  | + | -61.955 | 8 | 160.507 | 0.063 | 0.865 | 17.200 | 1.394 | 8.19E-07 | 4.886 | 5.179 | 5.540 | 4.229 | -- | 4.337 |
| 29 | + |  | + | + | + | -61.955 | 8 | 160.507 | 0.063 | 0.927 | 17.200 | 1.394 | 8.19E-07 | 5.540 | 5.179 | -- | 4.226 | 4.884 | 4.336 |
| 28 |  |  | + | + | + | -65.004 | 7 | 162.030 | 0.029 | 0.956 | 11.767 | 1.361 | 1.45E-04 | 5.319 | -- | -- | 4.201 | 4.879 | 4.324 |
| 7 | + | + | + |  |  | -65.574 | 7 | 163.170 | 0.017 | 0.973 | 11.359 | 1.361 | 2.29E-04 | 4.687 | 5.178 | 5.540 | 4.212 | -- | -- |
| 3 | + | + |  |  |  | -68.611 | 6 | 164.669 | 0.008 | 0.981 | 4.199 | 1.154 | 0.236 | 4.529 | 5.325 | 5.623 | -- | -- | -- |
| 20 |  |  | + |  | + | -68.614 | 6 | 164.676 | 0.008 | 0.989 | 5.034 | 1.199 | 0.088 | 5.257 | -- | -- | 4.001 | -- | 4.189 |
| 31 | + | + | + | + | + | -61.955 | 9 | 165.082 | 0.006 | 0.995 | 17.200 | 1.394 | 8.21E-07 | 0.030 | 5.179 | 5.540 | 4.244 | 4.896 | 4.343 |
| 19 | + | + |  |  | + | -68.271 | 7 | 168.566 | 1.11E-03 | 0.996 | 4.291 | 1.156 | 0.275 | 4.575 | 5.310 | 5.613 | -- | -- | 4.316 |
| 6 |  | + | + |  |  | -70.747 | 6 | 168.942 | 9.23E-04 | 0.997 | 4.489 | 1.197 | 0.166 | 4.987 | -- | 5.591 | 4.049 | -- | -- |
| 21 | + |  | + |  | + | -68.506 | 7 | 169.034 | 8.81E-04 | 0.998 | 5.008 | 1.197 | 0.126 | 5.292 | 5.211 | -- | 3.992 | -- | 4.182 |
| brown |  |  |  |  |  | -80.050 | 2 | 169.250 | 7.91E-04 | 0.999 | -- | 0.931 | 5.054 | -- | -- | -- | -- | -- | -- |
| 2 |  | + |  |  |  | -73.915 | 5 | 170.703 | 3.82E-04 | 0.999 | 2.216 | 1.078 | 1.377 | 5.178 | -- | 6.072 | -- | -- | -- |
| 4 |  |  | + |  |  | -74.463 | 5 | 171.799 | 2.21E-04 | 0.999 | 2.305 | 1.091 | 1.060 | 5.570 | -- | -- | 3.993 | -- | -- |
| 0 |  |  |  |  |  | -76.779 | 4 | 171.857 | 2.15E-04 | 0.999 | 1.406 | 1.038 | 1.959 | 6.031 | -- | -- | -- | -- | -- |
| 18 |  | + |  |  | + | -72.696 | 6 | 172.840 | 1.31E-04 | 1.000 | 2.562 | 1.092 | 1.285 | 5.151 | -- | 5.902 | -- | -- | 4.311 |
| 14 |  | + | + | + |  | -70.665 | 7 | 173.353 | 1.02E-04 | 1.000 | 4.676 | 1.206 | 0.184 | 5.003 | -- | 5.581 | 4.049 | 4.897 | -- |
| 16 |  |  |  |  | + | -75.459 | 5 | 173.791 | 8.17E-05 | 1.000 | 1.765 | 1.056 | 2.080 | 5.722 | -- | -- | -- | -- | 4.351 |
| 10 |  | + |  | + |  | -73.862 | 6 | 175.172 | 4.10E-05 | 1.000 | 2.233 | 1.079 | 1.962 | 5.071 | -- | 5.994 | -- | 5.276 | -- |
| 1 | + |  |  |  |  | -76.214 | 5 | 175.301 | 3.84E-05 | 1.000 | 1.467 | 1.038 | 3.432 | 5.393 | 6.051 | -- | -- | -- | -- |
| 12 |  |  | + | + |  | -73.996 | 6 | 175.441 | 3.58E-05 | 1.000 | 2.520 | 1.103 | 1.263 | 5.514 | -- | -- | 3.918 | 4.996 | -- |
| 5 | + |  | + |  |  | -74.287 | 6 | 176.022 | 2.68E-05 | 1.000 | 2.303 | 1.089 | 1.675 | 5.448 | 5.663 | -- | 3.967 | -- | -- |
| 8 |  |  |  | + |  | -76.699 | 5 | 176.272 | 2.36E-05 | 1.000 | 1.423 | 1.039 | 3.407 | 5.584 | -- | -- | -- | 5.140 | -- |
| 26 |  | + |  | + | + | -72.696 | 7 | 177.415 | 1.33E-05 | 1.000 | 2.562 | 1.092 | 1.704 | 5.115 | -- | 5.867 | -- | 5.119 | 4.277 |
| 17 | + |  |  |  | + | -75.188 | 6 | 177.825 | 1.09E-05 | 1.000 | 1.787 | 1.055 | 3.098 | 5.421 | 5.784 | -- | -- | -- | 4.200 |
| 24 |  |  |  | + | + | -75.206 | 6 | 177.861 | 1.07E-05 | 1.000 | 1.822 | 1.059 | 2.848 | 5.587 | -- | -- | -- | 5.013 | 4.161 |
| 9 | + |  |  | + |  | -76.203 | 6 | 179.855 | 3.94E-06 | 1.000 | 1.471 | 1.038 | 4.656 | 5.043 | 5.676 | -- | -- | 4.886 | -- |
| 13 | + |  | + | + |  | -73.954 | 7 | 179.930 | 3.79E-06 | 1.000 | 2.492 | 1.101 | 1.791 | 5.444 | 5.546 | -- | 3.894 | 4.975 | -- |
| 25 | + |  |  | + | + | -75.055 | 7 | 182.133 | 1.26E-06 | 1.000 | 1.825 | 1.057 | 3.812 | 5.325 | 5.601 | -- | -- | 4.890 | 4.027 |
|  | 0.8028 | 0.8980 | 0.4071 | 0.8059 | 0.3906 |  |  |  |  |  | 16.460 | 1.388 | 9.15E-03 | 4.540 | 5.180 | 5.541 | 4.217 | 4.886 | 4.331 |
|  |  |  |  |  |  |  |  |  |  |  |  |  | [1.01] | [93.67] | [177.75] | [255.02] | [67.83] | [132.49] | [76.03] |
