# Supplementary material for: Accelerated evolutionary rates in tropical and oceanic parmelioid lichens (Ascomycota)
Source: BMC Evol Biol. 2008 Sep 22;8:257. doi: 10.1186/1471-2148-8-257 (PMC2564941; doi:10.1186/1471-2148-8-257)
Supplement: Additional file 4 — Table S4 – Ecological parameters of specimens. [file 1471-2148-8-257-S4.doc]

| Species | Clade | HERBARIUM | Locality | Altitude (m) | Latitude | TempMax C(Avr) | TempMin. C(Avr.) | Precipitation (mm) | Index of Emberger (Q=100/(Tmax-Tmin) |  |  |  |  |  |  |  |  |  |
| --- | --- | --- | --- | --- | --- | --- | --- | --- | --- | --- | --- | --- | --- | --- | --- | --- | --- | --- |
| Bulbothrix apophysata | 2 | Lucking 16650b (L) | San Pedro, San José, COSTA RICA | 1100 | 9.57N, 84.02W | 21.4 | 19.0 | 1944 | 384.55 |  |  |  |  |  |  |  |  |  |
| Bulbothrix apophysata | 2 | US 1603 | La Vega, DOMINICAN REPUBLIC | 100 | 19.13N, 70.31W | 27 | 23.9 | 1418 | 232.38 |  |  |  |  |  |  |  |  |  |
| Bulbothrix apophysata | 2 | Hale, 18032 | La Vega, DOMINICAN REPUBLIC | 100 | 19.13N, 70.31W | 27 | 23.9 | 1418 | 232.38 |  |  |  |  |  |  |  |  |  |
| Bulbothrix coronata | 2 | MAF-Lich 13987 | Coegmansk loof near Ashton, Cape Prov., SOUTH AFRICA | 230 | 33.48 S, 20.06 E | 24.8 | 11.7 | 784 | 92.12 |  |  |  |  |  |  |  |  |  |
| Bulbothrix coronata | 2 | P 4192 | Balansa, PARAGUAY | 231 | 22.07S, 61.55W | 28.6 | 20 | 1090 | 111.69 |  |  |  |  |  |  |  |  |  |
| Bulbothrix coronata | 2 | LD sn | Mato Grosso, BRAZIL | 384 | 12.39S, 55.57W | 26.7 | 21.9 | 1577 | 173.17 |  |  |  |  |  |  |  |  |  |
| Bulbothrix decurtata | 2 | MAF-Lich 13988 | Hottentots Holland Nature Reserve,Cape Prov., SOUTH AFRICA | 1000 | 34.03 S, 19.01E | 20.2 | 4.0 | 1112 | 130.82 |  |  |  |  |  |  |  |  |  |
| Bulbothrix decurtata | 2 | 19Q | Paarl Mountain, Gordon's Way, Cape Prov., SOUTH AFRICA | 530 | 33.44S, 18.56E | 20.5 | 12.3 | 1008 | 161.5 |  |  |  |  |  |  |  |  |  |
| Bulbothrix decurtata | 2 | 1F | Harverd Poter National Garden, E. of Betty's Bay , SOUTH AFRICA | 61 | 34.20S, 18.55E | 20.5 | 12.3 | 1008 | 161.5 |  |  |  |  |  |  |  |  |  |
| Bulbothrix goebelii | 2 | MAF-Lich 13985 | Kristenbosch Botanic Garden, Cape Prov., SOUTH AFRICA | 140 | 33.50S, 18.25E | 21.0 | 12.0 | 936 | 125 |  |  |  |  |  |  |  |  |  |
| Bulbothrix goebelii | 2 | 36959Elix | Edmund Kennedy National Park, Queensland, AUSTRALIA | 2 | 18.12S, 146.00E | 27.5 | 19.4 | 1217 | 171 |  |  |  |  |  |  |  |  |  |
| Bulbothrix goebelii | 2 | ASSAM 2834 | Phek, Nagaland, INDIA | 1300 | 25.39N, 94.29E | 28.6 | 18.3 | 3163 | 346.15 |  |  |  |  |  |  |  |  |  |
| Bulbothrix meizospora | 2 | GPGC 02-000786 | Goriganga Catchment, Uttaranchal, INDIA | 1700 | 29.60N, 80.20E | 21 | 6 | 1301 | 210.95 |  |  |  |  |  |  |  |  |  |
| Bulbothrix meizospora | 2 | LWU 67288 | Kalimpong, Darjeeling, W. Bengal, INDIA | 1500 | 27.04N, 88.28E | 16.7 | 5.0 | 3030 | 857.13 |  |  |  |  |  |  |  |  |  |
| Bulbothrix meizospora | 2 | LWG sn | Shimla, Himachal Pradesh, INDIA | 2100 | 31.06N, 77.10E | 19.4 | 5.3 | 1577 | 306.21 |  |  |  |  |  |  |  |  |  |
| Bulbothrix setschwanensis | 2 | MAF-Lich 10212 | Chu Xiong, Chu Xiong County, Yunnan, CHINA | 2200 | 24.59N, 101.26E | 22.2 | 10.0 | 821 | 97.32 |  |  |  |  |  |  |  |  |  |
| Bulbothrix setschwanensis | 2 | LWG sn | Dalhousie, Himachal Pradesh, INDIA | 1827 | 32.3N, 75.58E | 19.4 | 5.3 | 1577 | 306.21 |  |  |  |  |  |  |  |  |  |
| Bulbothrix setschwanensis | 2 | LWG 97559 | Moreh, Manipur, INDIA | 450 | 24.49N, 93.53E | 28.6 | 18.3 | 3163 | 346.15 |  |  |  |  |  |  |  |  |  |
| Everniastrum cirrhatum | 1 | Trest 149 | Cerro de la Muerte, Pérez Zeledón, San José, COSTA RICA | 3300 | 9.56N, 83.75W | 20 | 8 | 2061 | 261.54 |  |  |  |  |  |  |  |  |  |
| Everniastrum cirrhatum | 1 | MAF 10374 | Jian Chuan County, Yunnan, CHINA | 3980 | 26.31N, 99.43E | 19.2 | 7.6 | 963 | 140.45 |  |  |  |  |  |  |  |  |  |
| Everniastrum cirrhatum | 1 | MAF-Lich 13976 | Parque Nacional de Huascarán, Quebrada Paron PERU | 3860 | 9.50S, 77.50W | 13 | 9.5 | 724 | 181 |  |  |  |  |  |  |  |  |  |
| Everniastrum lipidiferum | 1 | MAF-Lich13966 | Parque Nacional de Huascarán, Quebrada Cojup, PERU | 3900 | 9.50S, 77.50W | 13 | 9.5 | 724 | 181 |  |  |  |  |  |  |  |  |  |
| Everniastrum lipidiferum | 1 | DUKE 20064 | El Sumidero, Chiapas, MEXICO | 1439 | 16.15N, 92.18W | 16.2 | 12.1 | 1138 | 307.73 |  |  |  |  |  |  |  |  |  |
| Everniastrum lipidiferum | 1 | US 44514 | End of Laguna negra, Merida, VENEZUELA | 3930 | 8.34N, 71.00W | 22.8 | 21.4 | 1796 | 294.29 |  |  |  |  |  |  |  |  |  |
| Everniastrum nepalense | 1 | GPGC 02-000924 | Lamaghar, Uttaranchal, INDIA | 1600 | 29.60N, 80.20E | 21 | 6 | 1301 | 210.95 |  |  |  |  |  |  |  |  |  |
| Everniastrum nepalense | 1 | AWAS 1419 | Jakhu top, Shimla, Himachal Pradesh, INDIA | 2400 | 31.16N, 77.10E | 19.4 | 5.3 | 1577 | 306.21 |  |  |  |  |  |  |  |  |  |
| Everniastrum nepalense | 1 | 1675 Kurokawa | Chiang Mai Prov., THAILAND | 500 | 18.42N, 98.47E | 30 | 22.8 | 1288 | 108.16 |  |  |  |  |  |  |  |  |  |
| Everniastrum rhizodendroideum | 1 | ABL 55665 | Jian Chuan County., Yunnan, CHINA | 3900 | 26.37N, 99.43E | 19.2 | 7.6 | 963 | 140.45 |  |  |  |  |  |  |  |  |  |
| Everniastrum rhizodendroideum | 1 | HAMH-L 762 | Xizang, TIBET | 4100 | 31.55N, 88.21E | 11.7 | 5.3 | 1012 | 143.99 |  |  |  |  |  |  |  |  |  |
| Everniastrum rhizodendroideum | 1 | B 131 | Namche Bazar, Khumbhu Himal, NEPAL | 3440 | 27.48N, 86.42E | 24.7 | 10.3 | 1427 | 166.07 |  |  |  |  |  |  |  |  |  |
| Everniastrum sorocheilum | 1 | MAF-Lich 10375 | Jian Chuan County, Yunnan, CHINA | 3980 | 26.37N, 99.43E | 19.2 | 7.6 | 963 | 140.45 |  |  |  |  |  |  |  |  |  |
| Everniastrum sorocheilum | 1 | US 44555 | End of Laguna negra, Sierra Nevada, Mérida, VENEZUELA | 3930 | 8.34N, 71.00W | 22.8 | 21.4 | 1796 | 294.29 |  |  |  |  |  |  |  |  |  |
| Everniastrum sorocheilum | 1 | LWG 85879A | Chamoli district, Uttaranchal, INDIA | 3150 | 30.25N, 79.12E | 21 | 6 | 1301 | 210.95 |  |  |  |  |  |  |  |  |  |
| Everniastrum vexans | 1 | ABL56597 | Yunlong Co., Yunnan, CHINA | 2500 | 25.45N, 99.06E | 20.9 | 10.0 | 1056 | 148.23 |  |  |  |  |  |  |  |  |  |
| Everniastrum vexans | 1 | LWU 8469 | Idukki district, Kerala, INDIA | 1200 | 9.5N, 76.59E | 28.3 | 25.6 | 1696 | 357.53 |  |  |  |  |  |  |  |  |  |
| Everniastrum vexans | 1 | LWU 67281 | Kalimpong, Darjeeling district, W. Bengal, INDIA | 1600 | 27.04N, 88.28E | 16.7 | 5.0 | 3030 | 857.13 |  |  |  |  |  |  |  |  |  |
| Hypotrachyna adducta | 2 | MAF-Lich 10206 | San Jian Bin Liv area, Jian Chuan County, Yunnan, CHINA | 2448 | 26.39N, 99.47E | 19.2 | 7.6 | 963 | 140.45 |  |  |  |  |  |  |  |  |  |
| Hypotrachyna adducta | 2 | MAF-Lich 10378 | Yunlong County, Yunnan, CHINA | 2500 | 25.45N, 99.06E | 20.9 | 10.0 | 1056 | 148.23 |  |  |  |  |  |  |  |  |  |
| Hypotrachyna adducta | 2 | MAF-Lich 15400 | Namachi, Sikkim, INDIA | 1800 | 27.10N, 88.20E | 16.7 | 5.0 | 3030 | 857.13 |  |  |  |  |  |  |  |  |  |
| Hypotrachyna aff brevirhiza | 2 | MAF-Lich 10376 | Jian Chuan County, Yunnan, CHINA | 2490 | 26.21N, 99.50E | 19.2 | 7.6 | 963 | 140.45 |  |  |  |  |  |  |  |  |  |
| Hypotrachyna aff immaculata | 1 | MAF-Lich 10413 | Chu Xiong County, Yunnan, CHINA | 2200 | 24.59N, 101.26E | 22.2 | 10.0 | 821 | 97.32 |  |  |  |  |  |  |  |  |  |
| Hypotrachyna aff. taylorensis | 1 | MAF-Lich 10409 | Las Mercedes,Tenerife, Canary Island, SPAIN | 1200 | 28.32N, 16.12W | 19.1 | 13 | 518 | 102.45 |  |  |  |  |  |  |  |  |  |
| Hypotrachyna booralensis | 1 | MAF-Lich 13969 | Capricoru coast, Queesland, AUSTRALIA | 10 | 23.12S, 150.45E | 25.7 | 17.8 | 1258 | 140.24 |  |  |  |  |  |  |  |  |  |
| Hypotrachyna booralensis | 1 | MEL 16764 | New South Wales, west of Booral, AUSTRALIA | 67 | 32.28S, 152.00E | 25 | 9.2 | 672 | 62.89 |  |  |  |  |  |  |  |  |  |
| Hypotrachyna ciliata | 2 | MAF-Lich 10185 | Shibado Shao park, Jian Chuan County, Yunnan, CHINA | 2448 | 26.21N, 99.50E | 19.2 | 7.6 | 963 | 140.45 |  |  |  |  |  |  |  |  |  |
| Hypotrachyna ciliata | 2 | Wang 4975 | Mt. Yulongshan, Yunnan, CHINA | 2700 | 26.46N, 100E | 19.2 | 7.6 | 963 | 140.45 |  |  |  |  |  |  |  |  |  |
| Hypotrachyna ciliata | 2 | Wang 5523 | Zhongdian, Yunnan, CHINA | 3279 | 27.48N, 99.42E | 26.1 | 4.2 | 1281 | 136.83 |  |  |  |  |  |  |  |  |  |
| Hypotrachyna costaricensis | 2 | MAF-Lich 10211 | Volcán Arenal, COSTA RICA | 500 | 10.48N, 84.85W | 25 | 17 | 2669 | 269.9 |  |  |  |  |  |  |  |  |  |
| Hypotrachyna costaricensis | 2 | 57 sergio | Sierra Maestra, Gramma, CUBA | 1700 | 20N, 77.49W | 27.5 | 22.2 | 1420 | 182.03 |  |  |  |  |  |  |  |  |  |
| Hypotrachyna costaricensis | 2 | MAF-Lich 2552 | Sierra Portuguessa, Lara Prov., VENEZUELA | 1900 | 10.08N, 69.52W | 22.8 | 21.4 | 1796 | 294.29 |  |  |  |  |  |  |  |  |  |
| Hypotrachyna crenata | 2 | MAF-Lich 10377 | Heqing County,Yunnan, CHINA | 2400 | 26.13N, 100.09E | 19.2 | 7.6 | 963 | 140.45 |  |  |  |  |  |  |  |  |  |
| Hypotrachyna crenata | 2 | LWG sn | Shillong, Meghalaya, INDIA | 900 | 25.34N, 91.52E | 28.6 | 18.3 | 3163 | 346.15 |  |  |  |  |  |  |  |  |  |
| Hypotrachyna crenata | 2 | LWU 6754 | Tiger hill, Darjeeling, W. Bengal, INDIA | 2550 | 26.59N, 88.17E | 16.7 | 5.0 | 3030 | 857.13 |  |  |  |  |  |  |  |  |  |
| Hypotrachyna endochlora | 1 | MAF-Lich 10178 | Crinanwood, Kintyre, Scotland, GREAT BRITAIN | 10 | 56.05N, 5.33W | 16.4 | 7 | 869 | 247.3 |  |  |  |  |  |  |  |  |  |
| Hypotrachyna endochlora | 1 | MAF-Lich 10379 | Las Mercedes, Tenerife, CANARY ISLAND | 900 | 28.30N, 16.11W | 19.1 | 13 | 518 | 102.45 |  |  |  |  |  |  |  |  |  |
| Hypotrachyna endochlora | 1 | MSC 3509 | La Vega, DOMINICAN REPUBLIC | 100 | 19.13N, 70.31W | 27 | 23.9 | 1418 | 232.38 |  |  |  |  |  |  |  |  |  |
| Hypotrchyna exsecta | 1 | MAF 10380 | Lunan County, Yunnan CHINA | 1909 | 24.48N, 103.17E | 20.8 | 8.1 | 1039 | 153.14 |  |  |  |  |  |  |  |  |  |
| Hypotrachyna exsecta | 1 | LWG 18400 | Berinag, Pithoragarh district, Uttaranchal, INDIA | 1900 | 29.35N, 80.11E | 21 | 6 | 1301 | 210.95 |  |  |  |  |  |  |  |  |  |
| Hypotrachyna exsecta | 1 | LWU 71363 | Avlanche, Nilgiri hills, Tamil Nadu, INDIA | 2100 | 11.24N, 76.41E | 28.5 | 21.7 | 1398 | 159.59 |  |  |  |  |  |  |  |  |  |
| Hypotrachyna flexilis | 2 | MAF-Lich 13975 | Above Lachung towards Yumthang, Sikkim, INDIA | 3000 | 27.42N, 88.45E | 16.7 | 5.0 | 3030 | 857.13 |  |  |  |  |  |  |  |  |  |
| Hypotrachyna flexilis | 2 | LWU 67232 | Kalimpong, Darjeeling district, W. Bengal, INDIA | 1500 | 27.04N, 88.28E | 16.7 | 5.0 | 3037 | 857.13 |  |  |  |  |  |  |  |  |  |
| Hypotrachyna flexilis | 2 | LWU 7697 | Dailekh, Bheri zone, NEPAL | 2100 | 28.46N, 81.51E | 25 | 12.5 | 3388 | 397.63 |  |  |  |  |  |  |  |  |  |
| Hypotrachyna imbricatula | 1 | MAF-Lich 13990 | Hottentots Holland Nature Reserve, Cape Prov., SOUTH AFRICA | 1000 | 34.03 S, 19.01E | 20.2 | 4.0 | 1112 | 130.82 |  |  |  |  |  |  |  |  |  |
| Hypotrachyna imbricatula | 1 | MICH sn | Itaperica, Sao Paulo, BRAZIL | 10 | 12.53S, 38.40W | 26.9 | 22.5 | 1069 | 119.12 |  |  |  |  |  |  |  |  |  |
| Hypotrachyna imbricatula | 1 | BRI 141 | Lamington National Park, moon light carg, Queensland, AUSTRALIA | 519 | 28.14S, 153.07E | 22.0 | 10.6 | 927 | 123.94 |  |  |  |  |  |  |  |  |  |
| Hypotrachyna immaculata | 1 | MAF-Lich 7462 | Morton National Park, Pidgeon House Mountain, AUSTRALIA | 750 | 34.59S 150.25E | 17 | 9.1 | 1511 | 286.54 |  |  |  |  |  |  |  |  |  |
| Hypotrachyna immaculata | 1 | MAF-Lich 10383 | Jian Chuan County, Yunnan, CHINA | 2450 | 26.21N, 99.5E | 19.2 | 7.6 | 963 | 140.45 |  |  |  |  |  |  |  |  |  |
| Hypotrachyna immaculata | 1 | 36486Elix | New South Wales, Oxley wild rivers National Park, AUSTRALIA | 945 | 30.40S, 151.43E | 22.0 | 10.6 | 927 | 123.94 |  |  |  |  |  |  |  |  |  |
| Hypotrachyna incognita | 2 | MAF-Lich 10385 | Dali County, Yunnan, CHINA | 3500 | 25.41N, 100.06E | 20.9 | 10.0 | 1056 | 148.23 |  |  |  |  |  |  |  |  |  |
| Hypotrachyna incognita | 2 | MAF-Lich 10384 | Jian Chuan County, Yunnan, CHINA | 2450 | 26.21N, 99.5E | 19.2 | 7.6 | 963 | 140.45 |  |  |  |  |  |  |  |  |  |
| Hypotrachyna incognita | 2 | AWAS 7896 | Elephant fall, Shillong, Meghalaya, INDIA | 1600 | 25.30N, 91.62E | 28.6 | 18.3 | 3163 | 346.15 |  |  |  |  |  |  |  |  |  |
| Hypotrachyna infirma | 2 | MAF-Lich 10210 | Yu Long Shan, Lijian County, Jade Dragon Snow Mountain, Yunnan, CHINA | 2700 | 26.46N, 100E | 19.2 | 7.6 | 963 | 140.45 |  |  |  |  |  |  |  |  |  |
| Hypotrachyna infirma | 2 | MAF-Lich 10386 | Yunlong County, Yunnan, CHINA | 2410 | 25.45N, 99.06E | 20.9 | 10.0 | 1056 | 148.23 |  |  |  |  |  |  |  |  |  |
| Hypotrachyna infirma | 2 | LWU 67312 | Kalimpong, Darjeeling district, W. Bengal, INDIA | 1500 | 27N, 88.28E | 16.7 | 5.0 | 3037 | 857.13 |  |  |  |  |  |  |  |  |  |
| Hypotrachyna koyaensis | 2 | MAF-Lich 10388 | Yunlong County, Yunnan, CHINA | 2500 | 25.45N, 99.06E | 20.9 | 10.0 | 1056 | 148.23 |  |  |  |  |  |  |  |  |  |
| Hypotrachyna koyaensis | 2 | AWAS 3924 | Kurseong, Darjeeling, district, W. Bengal, INDIA | 1650 | 26.52N, 88.16E | 16.7 | 5.0 | 3037 | 857.13 |  |  |  |  |  |  |  |  |  |
| Hypotrachyna koyaensis | 2 | LWU 76254 | Dailekh, Bheri zone, NEPAL | 2100 | 28.46N, 81.51E | 25 | 12.5 | 3388 | 397.63 |  |  |  |  |  |  |  |  |  |
| Hypotrachyna laevigata | 1 | MAF-Lich 10177 | Tayvallich, Argyll and Bute, Scotland, GREAT BRITAIN | 13 | 56.01N, 5.37W | 16.4 | 7 | 869 | 247.3 |  |  |  |  |  |  |  |  |  |
| Hypotrachyna laevigata | 1 | MAF-Lich 6975 | Las Mercedes, Tenerife, CANARY ISLAND | 900 | 28.30N, 16.11W | 19.1 | 13 | 518 | 102.45 |  |  |  |  |  |  |  |  |  |
| Hypotrachyna laevigata | 1 | ANUC 40144 | Arthur-Pieman, protected area, Tasmania, AUSTRALIA | 335 | 41.30S, 145E | 18.2 | 7.4 | 742 | 120.23 |  |  |  |  |  |  |  |  |  |
| Hypotrachyna neodissecta | 1 | MAF-Lich 13986 | Kristenbosch Botanic Garden, Cape Prov., SOUTH AFRICA | 140 | 33.50S, 18.25E | 21.0 | 12.0 | 936 | 125 |  |  |  |  |  |  |  |  |  |
| Hypotrachyna neodissecta | 1 | MAF-Lich 15416 | Mount Elgan Forest, Bukusu distt., Western Prov., KENYA | 2537 | 0.56N, 34.38E | 20 | 17.2 | 1140 | 175.53 |  |  |  |  |  |  |  |  |  |
| Hypotrachyna neodissecta | 1 | MAF-Lich 15385 | Kodaikanal, Palni hills, INDIA | 2200 | 11.24N, 76.43E | 28.5 | 21.7 | 1398 | 159.59 |  |  |  |  |  |  |  |  |  |
| Hypotrachyna osseoalba | 1 | MAF-Lich 10390 | Chu Xiong County, Yunnan, CHINA | 2200 | 24.59N, 101.26E | 22.2 | 10.0 | 821 | 97.32 |  |  |  |  |  |  |  |  |  |
| Hypotrachyna osseoalba | 1 | MAF-Lich 10389 | Lunan County, Yunnan, CHINA | 1909 | 24.43N, 103.20E | 20.8 | 8.1 | 1039 | 153.14 |  |  |  |  |  |  |  |  |  |
| Hypotrachyna osseoalba | 1 | MAF-Lich 9663 | Morton National Park, Pidgeon House Mountain, AUSTRALIA | 750 | 34.59S 150.25E | 19.2 | 6.9 | 1511 | 228.18 |  |  |  |  |  |  |  |  |  |
| Hypotrachyna physcioides | 1 | MAF-Lich 10391 | Dali County,Yunnan, CHINA | 3150 | 25.41N, 100.06E | 20.9 | 10.0 | 1056 | 148.23 |  |  |  |  |  |  |  |  |  |
| Hypotrachyna physcioides | 1 | MAF-Lich 10412 | Jian Chuan County, Yunnan, CHINA | 3200 | 26.31N, 99.43E | 19.2 | 7.6 | 963 | 140.45 |  |  |  |  |  |  |  |  |  |
| Hypotrachyna physcioides | 1 | MAF-Lich 15404 | Pithoragarh district, Uttaranchal, INDIA | 1800 | 29.35N, 80.11E | 21 | 6 | 1301 | 210.95 |  |  |  |  |  |  |  |  |  |
| Hypotrachyna pseudosinuosa | 1 | MAF-Lich 10392 | Lunan County, Yunnan, CHINA | 1909 | 24.48N, 103.17E | 20.8 | 8.1 | 1039 | 153.14 |  |  |  |  |  |  |  |  |  |
| Hypotrachyna pseudosinuosa | 1 | MAF-Lich 10393 | Lunan County, Yunnan, CHINA | 1909 | 24.48N, 103.17E | 20.8 | 8.1 | 1039 | 153.14 |  |  |  |  |  |  |  |  |  |
| Hypotrachyna pseudosinuosa | 1 | MAF-Lich 15386 | Tumin area, East Sikkim, INDIA | 2000 | 27.20N, 88.15E | 16.7 | 5.0 | 3030 | 857.13 |  |  |  |  |  |  |  |  |  |
| Hypotrachyna revoluta | 1 | MAF-Lich 6047 | Puerto Urkiola, Vizcaya, SPAIN | 800 | 40.06N, 2.38W | 16.5 | 2.4 | 1549 | 277.56 |  |  |  |  |  |  |  |  |  |
| Hypotrachyna revoluta | 1 | MAF-Lich 10394 | Chu Xiong County, Yunnan, CHINA | 2200 | 24.59N, 101.26E | 22.2 | 10.0 | 821 | 97.32 |  |  |  |  |  |  |  |  |  |
| Hypotrachyna revoluta | 1 | MAF-Lich 10406 | Las Mercedes,Tenerife, CANARY ISLAND | 1200 | 28.32N, 16.12W | 19.1 | 13 | 518 | 102.45 |  |  |  |  |  |  |  |  |  |
| Hypotrachyna rockii | 1 | MAF-Lich 13965 | Parque Nacional de Huascaran, Quebrada Paron, PERU | 3860 | 9.50S, 77.50W | 13 | 9.5 | 724 | 181 |  |  |  |  |  |  |  |  |  |
| Hypotrachyna rockii | 1 | MAF-Lich 2919 | Sierra Portugues, Lara Prov., VENEZUELA | 1900 | 10.08N, 69.52W | 22.8 | 21.4 | 1796 | 294.29 |  |  |  |  |  |  |  |  |  |
| Hypotrachyna rockii | 1 | LWU 70264 | Kodaikanal, Palni hills, INDIA | 2250 | 11.24N, 76.43E | 28.5 | 21.7 | 1398 | 159.59 |  |  |  |  |  |  |  |  |  |
| Hypotrachyna scytophylla | 2 | MAF-Lich 10410 | Jian Chuan County,Yunnan, CHINA | 2500 | 26.21N, 99.50E | 19.2 | 7.6 | 963 | 140.45 |  |  |  |  |  |  |  |  |  |
| Hypotrachyna scytophylla | 2 | MAF-Lich 13973 | Kullu district, Himachal Pradesh, INDIA | 3000 | 32.01N, 77.02E | 19.4 | 5.3 | 1577 | 306.21 |  |  |  |  |  |  |  |  |  |
| Hypotrachyna scytophylla | 2 | LWU 67419 | Sandakhpoo, Darjeeling, district, W. Bengal, INDIA | 3500 | 26.59N, 88.17E | 16.7 | 5.0 | 3037 | 857.13 |  |  |  |  |  |  |  |  |  |
| Hypotrachyna sinuosa | 1 | MAF-Lich 10179 | Apple cross, Wester Ross, Scotland, GREAT BRITAIN | 63 | 57.26N, 5.49W | 12.5 | 3.6 | 761 | 342.8 |  |  |  |  |  |  |  |  |  |
| Hypotrachyna sinuosa | 1 | MAF-Lich 10396 | Jian Chuan County, Yunnan, CHINA | 3200 | 26.31N, 99.43E | 19.2 | 7.6 | 963 | 140.45 |  |  |  |  |  |  |  |  |  |
| Hypotrachyna sinuosa | 1 | MAF-Lich 15393 | Mt. Albert Edward sumit area, PAPUA NEW GUINEA | 3700 | 8.23S, 147.23E | 26.9 | 17.1 | 1011 | 185.85 |  |  |  |  |  |  |  |  |  |
| Hypotrachyna taylorensis | 1 | MAF-Lich 9912 | Tayvallich, Argyll and Bute, Scotland, GREAT BRITAIN | 13 | 56.01N, 5.37W | 16.4 | 7 | 869 | 247.3 |  |  |  |  |  |  |  |  |  |
| Hypotrachyna taylorensis | 1 | MAF-Lich 10405 | Las Mercedes, Tenerife, CANARY ISLAND | 900 | 28.30N, 16.11W | 19.1 | 13 | 518 | 102.45 |  |  |  |  |  |  |  |  |  |
| Hypotrachyna taylorensis | 1 | MAF-Lich 10407 | Gondomar, Pontavedra, SPAIN | 29 | 42.06N, 8.45W | 20.5 | 9.2 | 1595 | 250.79 |  |  |  |  |  |  |  |  |  |
| Karoowia saxeti | 3 | Aproot 53350 | Kenting National Park, Pingtung County, TAIWAN | 300 | 22.40N, 120.28E | 28.3 | 16.7 | 1039 | 142.57 |  |  |  |  |  |  |  |  |  |
| Karoowia saxeti | 3 | Degelius s.n. | Bimbe, Huila, ANGOLA | 1300 | 15.04S, 16.39E | 23.9 | 17.8 | 1027 | 106.98 |  |  |  |  |  |  |  |  |  |
| Karoowia saxeti | 3 | AA 3310 | Umtamvuna, Nature Reserve, Natal, SOUTH AFRICA | 899 | 28.56S, 30.48E | 20.4 | 11.4 | 897 | 139.01 |  |  |  |  |  |  |  |  |  |
| Melanohalea aff. elegantula | 5 | 16550 Esslinger | Wheeler County, Umatilla National forest, Oregón, USA | 1270 | 44.56N, 119.42W | 20.3 | -3.6 | 289 | 35.23 |  |  |  |  |  |  |  |  |  |
| Melanelia aff. elegantula | 5 | 16362 Esslinger | Idaho, Kooteniv County, Oregón, USA | 665 | 47.40N, 116.46W | 24.7 | -2.5 | 236 | 22.24 |  |  |  |  |  |  |  |  |  |
| Melanelia aff. elegantula | 5 | H 2208 | Wild basin, Boulder co., Colorado, USA | 1665 | 40.00N, 105.08W | 23.1 | -1.1 | 387 | 43.07 |  |  |  |  |  |  |  |  |  |
| Melanohalea aff. exasperata | 5 | MAF-Lich 10227 | Lago del Valle, Parque Natural de Somiedo, Asturias, SPAIN | 1500 | 43.04N, 6.11W | 18.8 | 1.7 | 936 | 136.05 |  |  |  |  |  |  |  |  |  |
| Melanelia aff. exasperata | 5 | MAF 10230 | Parque Natural Somiedo, Lago del Valle, Asturias, SPAIN | 1500 | 43.04N, 6.11W | 18.8 | 1.7 | 936 | 136.05 |  |  |  |  |  |  |  |  |  |
| Melanelia aff. exasperata | 5 | MAF 10225 | Las Médulas, Leon, SPAIN | 663 | 42.28N, 6.46W | 21 | 4.8 | 901 | 109.49 |  |  |  |  |  |  |  |  |  |
| Melanohalea elegantula | 5 | MAF-Lich 10218 | Mataelpino, Madrid, SPAIN | 1040 | 40.44N, 3.56W | 19.9 | 3.4 | 725 | 96.7 |  |  |  |  |  |  |  |  |  |
| Melanohalea elegantula | 5 | MAF-Lich 10226 | Cercedilla, Madrid, SPAIN | 1300 | 40.44N, 4.03W | 19.9 | 3.4 | 725 | 96.7 |  |  |  |  |  |  |  |  |  |
| Melanohalea elegantula | 5 | MAF-Lich 10218 | Corachar, Castellón, SPAIN | 1160 | 40.40N, 0.05E | 17.3 | 2 | 784 | 137.72 |  |  |  |  |  |  |  |  |  |
| Melanohalea exasperata | 5 | MAF-Lich 7636 | Sierra de Grazalema, Cádiz, SPAIN | 1100 | 36.45N, 5.22W | 25.2 | 7.5 | 1962 | 208.99 |  |  |  |  |  |  |  |  |  |
| Melanohalea exasperata | 5 | MAF-Lich 11386 | Torremocha, Guadalajara, SPAIN | 450 | 40.58N, 2.37W | 21.7 | 2.8 | 601 | 60.84 |  |  |  |  |  |  |  |  |  |
| Melanohalea exasperata | 5 | MAF-Lich 13059 | Herbes, Castellón, SPAIN | 850 | 40.37N, 0.04E | 20.3 | 3.6 | 599 | 86.6 |  |  |  |  |  |  |  |  |  |
| Melanohalea exasperatula | 5 | MAF-Lich 10213 | Cercedilla, Madrid, SPAIN | 1200 | 40.44N, 4.03W | 19.9 | 3.4 | 725 | 96.7 |  |  |  |  |  |  |  |  |  |
| Melanohalea exasperatula | 5 | MAF-Lich 13093 | Corachar, Castellón, SPAIN | 1160 | 40.40N, 0.05E | 17.3 | 2 | 784 | 137.72 |  |  |  |  |  |  |  |  |  |
| Melanohalea exasperatula | 5 | MAF-Lich 5157 | Niedere, Tauern, Steiermark, AUSTRIA | 1340 | 47.19N, 13.46E | 21.1 | -1 | 452 | 62.01 |  |  |  |  |  |  |  |  |  |
| Melanohalea olivacea | 5 | Vitikanen 16196 | Kn. Puolanca, NW end of Ristijärvi, FINLAND | 150 | 64.52N, 27.40E | 11.4 | -8.2 | 544 | 232.12 |  |  |  |  |  |  |  |  |  |
| Melanohalea olivacea | 5 | Trelease 1899 (US) | Glacier Bay, Eastern pacific Coast Distr., Alaska, USA | 2800 | 61.08N, 141.08W | 11.1 | -16.7 | 525 | -335.23 |  |  |  |  |  |  |  |  |  |
| Melanohalea olivacea | 5 | Lynge 1914 (US) | Nordbynesset, Troms, NORWAY | 164 | 69.17N, 18.59E | 12.8 | -16.4 | 335 | -212.22 |  |  |  |  |  |  |  |  |  |
| Melanohalea septentrionalis | 5 | Athi 60893 | Keski-Pohjanmaa, FINLAND | 103 | 63.40N, 23.56E | 17.2 | -10 | 640 | 208.44 |  |  |  |  |  |  |  |  |  |
| Melanohalea septentrionalis | 5 | MAF-Lich 5325 | Larimer County, Rocky Mont National Park, Colorado, USA | 900 | 40.25N, 105.31W | 23.1 | -1.1 | 387 | 43.07 |  |  |  |  |  |  |  |  |  |
| Melanohalea septentrionalis | 5 | Llano 462a (US) | Anaktuvuk Pass, Erctic Coast District, Alaska, USA | 1250 | 68.05N, 151.50W | 14.7 | -24.5 | 334 | -85.72 |  |  |  |  |  |  |  |  |  |
| Melanohalea subelegantula | 5 | Esslinger 16132 | Prairie city, Grant County, Oregón, USA | 1585 | 44.27N, 118.42W | 20.3 | -3.6 | 289 | 35.23 |  |  |  |  |  |  |  |  |  |
| Melanohalea subelegantula | 5 | Esslinger 14037 | Gifford Pinchot National forest, Wallowa County, Oregon, USA | 1700 | 45.47N, 121.37W | 18.9 | -0.3 | 230 | 27.61 |  |  |  |  |  |  |  |  |  |
| Melanohalea subelegantula | 5 | Esslinger 1715 | Southeast of Enterprise along the Lostine river, Wallowa County, Oregón, USA | 1700 | 45.27N, 117.28W | 24.7 | -2.5 | 236 | 20.09 |  |  |  |  |  |  |  |  |  |
| Melanohalea subolivacea | 5 | 16555 Esslinger | Wheeler County, Umatilla National forest, Oregón, USA | 1270 | 44.56N, 119.42E | 20.3 | -3.6 | 289 | 35.23 |  |  |  |  |  |  |  |  |  |
| Melanohalea subolivacea | 5 | 15576 Esslinger | Cochise County, Coronado National forest, Arizona, USA | 2550 | 31.55N, 109.16W | 24.2 | 2.5 | 310 | 30.66 |  |  |  |  |  |  |  |  |  |
| Melanohalea subolivacea | 5 | 16555 Esslinger | Apache County, Mountain Baldy, Wilderness, Arizona, USA | 2900 | 33.55N, 109.30W | 20 | -0.6 | 557 | 72.2 |  |  |  |  |  |  |  |  |  |
| Parmelaria subthomsonii | 4 | LWG 20-77151 (MAF-Lich 7654) | Tsomgo lake,Sikkim, INDIA | 3700 | 27.23N, 88.45E | 21 | 6 | 1301 | 210.95 |  |  |  |  |  |  |  |  |  |
| Parmelaria subthomsonii | 4 | LWG 3979 | Pithoragarh distt., Uttaranchal, INDIA | 1650 | 29.35N, 80.11E | 21 | 6 | 1301 | 210.95 |  |  |  |  |  |  |  |  |  |
| Parmelinella wallichiana | 2 | LWG 20-77171 (MAF-Lich 7653) | Gangtok, Sikkim, INDIA | 1750 | 27.20N, 88.36E | 16.7 | 5.0 | 3030 | 857.13 |  |  |  |  |  |  |  |  |  |
| Parmelinella wallichiana | 2 | MAF 10411 | Jian Chuan County, Yunnan, CHINA | 2500 | 26.22N, 99.49E | 19.2 | 7.6 | 963 | 140.45 |  |  |  |  |  |  |  |  |  |
| Parmelinella wallichiana | 2 | LWU 76212 | Ratangla, Bherizone, NEPAL | 2400 | 28.46N, 81.51E | 25 | 12.5 | 3388 | 397.63 |  |  |  |  |  |  |  |  |  |
| Parmelinopsis cryptochlora | 1 | MAF-Lich 10398 | Chu Xiong County, Yunnan, CHINA | 2200 | 24.59N, 101.26E | 22.2 | 10.0 | 821 | 97.32 |  |  |  |  |  |  |  |  |  |
| Parmelinopsis cryptochlora | 1 | 40203 Hale | Nilgiri hills, Tamil Nadu, INDIA | 2100 | 11.24N, 76.41E | 28.5 | 21.7 | 1398 | 159.59 |  |  |  |  |  |  |  |  |  |
| Parmelinopsis cryptochlora | 1 | BM 912 | Laudat, DOMINICA | 548 | 15.19N, 61.19W | 27 | 23.6 | 1769 | 290.02 |  |  |  |  |  |  |  |  |  |
| Parmelinopsis horrescens | 1 | MAF-Lich 9913 | Viduido, Bentin, La Coruña, SPAIN | 350 | 42.50N, 8.36W | 18.4 | 7.6 | 1545 | 278.68 |  |  |  |  |  |  |  |  |  |
| Parmelinopsis horrescens | 1 | MAF-Lich 10399 | Las Mercedes, Tenerife, CANARY ISLAND | 900 | 28.30N, 16.11W | 19.1 | 13 | 518 | 102.45 |  |  |  |  |  |  |  |  |  |
| Parmelinopsis horrescens | 1 | MAF-Lich 10400 | Gondomar, Pontevedra, SPAIN | 20 | 42.06N, 8.45W | 20.5 | 9.2 | 1595 | 250.79 |  |  |  |  |  |  |  |  |  |
| Parmelinopsis minarum | 1 | MAF-Lich 7639 | Parque Natural de los Alcornocales, Facinas, Cádiz, SPAIN | 220 | 36.08N, 5.41W | 21.8 | 11.6 | 1065 | 130.9 |  |  |  |  |  |  |  |  |  |
| Parmelinopsis minarum | 1 | MAF-Lich 10401 | Las Mercedes, Tenerife, CANARY ISLAND | 900 | 28.30N, 16.11W | 19.1 | 13 | 518 | 102.45 |  |  |  |  |  |  |  |  |  |
| Parmelinopsis minarum | 1 | 16753 Elix | Tully Falls Rd, Queensland, AUSTRALIA | 864 | 17.43S, 145.32E | 27.8 | 22.5 | 1773 | 273.73 |  |  |  |  |  |  |  |  |  |
| Parmelinopsis neodamaziana | 1 | MAF-Lich 10182 | Morton National Park, Pidgeon House Mountain, AUSTRALIA | 750 | 34.59S 150.25E | 19.2 | 6.9 | 1511 | 228.18 |  |  |  |  |  |  |  |  |  |
| Parmelinopsis neodamaziana | 1 | 37258 Elix | New South Wales, Wash pool national park, Gibraltar range, AUSTRALIA | 895 | 29.28S, 152.21E | 22.2 | 7.8 | 761 | 84.5 |  |  |  |  |  |  |  |  |  |
| Parmelinopsis neodamaziana | 1 | 2343 Elix | New South Wales, 15 km W of Dorrigo, AUSTRALIA | 751 | 30.20S, 152.40E | 22.8 | 12.5 | 1612 | 241.29 |  |  |  |  |  |  |  |  |  |
| Parmelinopsis subfatiscens | 1 | MAF-Lich 6878 | Morton National Park, AUSTRALIA | 750 | 34.59S 150.25E | 19.2 | 6.9 | 1511 | 228.18 |  |  |  |  |  |  |  |  |  |
| Parmelinopsis subfatiscens | 1 | MAF-Lich 15412 | Whangarei, North-land region, NEW ZEALAND | 100 | 35.43S, 174.17E | 18.3 | 8.9 | 2017 | 382.22 |  |  |  |  |  |  |  |  |  |
| Parmelinopsis subfatiscens | 1 | ANUC 4038 | Australian capital territory 11 Km SW of Tharwa, AUSTRALIA | 1135 | 35.38S, 148.58E | 20.8 | 12.1 | 1258 | 252.59 |  |  |  |  |  |  |  |  |  |
| Parmotrema cetratum | 4 | Osorio 9424 | Sierra de la Coronilla, cerca del cerro Catedral, Maldonado, URUGUAY | 250 | 34.21S, 54.38W | 23.3 | 11.7 | 1125 | 132.6 |  |  |  |  |  |  |  |  |  |
| Parmotrema cetratum | 4 | Osorio 9425 | Sierra de la Coronilla, cerca del cerro Catedral, Maldonado, URUGUAY | 250 | 34.21S, 54.38W | 23.3 | 11.7 | 1125 | 132.6 |  |  |  |  |  |  |  |  |  |
| Parmotrema cetratum | 4 | H-ACH sn | Pennsylvania, USA | 441 | 41N, 77.30W | 18.1 | -6.7 | 1009 | 213.62 |  |  |  |  |  |  |  |  |  |
| Parmotrema crinitum | 4 | MAF-Lich 6061 | Castello do Mouros, Sintra, PORTUGAL | 400 | 38.47N, 9.23W | 18.2 | 11.7 | 803 | 188.49 |  |  |  |  |  |  |  |  |  |
| Parmotrema crinitum | 4 | MAF-Lich 9891 | La Gomera, Tenerife, CANARY ISLAND | 1070 | 28.06N, 17.12W | 22.6 | 16.8 | 438 | 94.13 |  |  |  |  |  |  |  |  |  |
| Parmotrema crinitum | 4 | MAF-Lich 7605 | 13 km SSE batemats bay, Melville Point, AUSTRALIA | 1 | 35.72S, 150.18E | 19.7 | 13.6 | 1038 | 214.62 |  |  |  |  |  |  |  |  |  |
| Parmotrema fistulatum | 4 | Osorio 9423 (MAF-Lich 7655) | Pozo del Buey, cerro Catedral, Maldonado, URUGUAY | 100 | 34.25S, 54.33W | 23.3 | 11.7 | 1125 | 132.6 |  |  |  |  |  |  |  |  |  |
| Parmotrema fistulatum | 4 | Osorio 5671 | Laguna negra, Rocha, URUGAY | 143 | 34.00S, 53.58W | 22.8 | 11.1 | 1032 | 130.53 |  |  |  |  |  |  |  |  |  |
| Parmotrema fistulatum | 4 | US 20954 | Tandil, Buenos Aires, ARGENTINA | 176 | 37.14S, 59.14W | 21.4 | 7.5 | 791 | 88.36 |  |  |  |  |  |  |  |  |  |
| Parmotrema haitiense | 4 | MAF-Lich 7657 | Australian Capital Territory, Paddy River near Murrays Corner, AUSTRALIA | 550 | 35.22S, 148.58E | 19 | 5.1 | 976 | 118.74 |  |  |  |  |  |  |  |  |  |
| Parmotrema haitiense | 4 | CBG 7784 | New South Wales, Goulbourn, Wombeyancaves, AUSTRALIA | 818 | 35.19S, 150.01E | 20.8 | 12.1 | 1258 | 252.59 |  |  |  |  |  |  |  |  |  |
| Parmotrema haitiense | 4 | G 71 | Caracas, VENEZUELA | 897 | 10.29N, 66.53W | 28.1 | 25.8 | 882 | 172.14 |  |  |  |  |  |  |  |  |  |
| Parmotrema hypoleucinum | 4 | MAF-Lich 7637 | Parque Natural de los Alcornocales, Cádiz, SPAIN | 220 | 36.08N, 5.41W | 21.8 | 11.6 | 1065 | 130.9 |  |  |  |  |  |  |  |  |  |
| Parmotrema hypoleucinum | 4 | MAF-Lich 13143 | Villabona a Rosell, Castellón, SPAIN | 500 | 40.37N, 0.12E | 23.1 | 8.4 | 611 | 71.43 |  |  |  |  |  |  |  |  |  |
| Parmotrema hypoleucinum | 4 | 3p | Maamora, Rabat, MOROCCO | 68 | 34.01N, 6.42W | 23.1 | 12.5 | 506 | 68.31 |  |  |  |  |  |  |  |  |  |
| Parmotrema perforatum | 4 | Cole 7983 | Greene County, North Carolina, USA | 98 | 35.33N, 77.48W | 25.2 | 4.3 | 1072 | 112.47 |  |  |  |  |  |  |  |  |  |
| Parmotrema perforatum | 4 | MAF-Lich 1165 | Hardin County, 2km SE of obsaratoga, Texas, USA | 26 | 30.17N, 94.31W | 28.5 | 12.7 | 1063 | 124.11 |  |  |  |  |  |  |  |  |  |
| Parmotrema perforatum | 4 | MAF-Lich 1153 | Brazos County, 9km Nw of Bryan, Texas, USA | 100 | 30.49N, 96.31W | 29.5 | 11.1 | 899 | 70.66 |  |  |  |  |  |  |  |  |  |
| Parmotrema perlatum | 4 | MAF-Lich 6965 | Castello do Mouros, Sintra, PORTUGAL | 400 | 38.47N, 9.23W | 18.2 | 11.7 | 803 | 188.49 |  |  |  |  |  |  |  |  |  |
| Parmotrema perlatum | 4 | MAF-Lich 15331 | Braganza, Tras-os-Montes, PORTUGAL | 680 | 41.49N, 6.46W | 18.9 | 3.4 | 1195 | 204.4 |  |  |  |  |  |  |  |  |  |
| Parmotrema perlatum | 4 | MAF-Lich 7498 | Australian Capital territory, Molonglo Gorge Reserve, AUSTRALIA | 550 | 35.20S, 149.15E | 20.6 | 6.3 | 610 | 72.44 |  |  |  |  |  |  |  |  |  |
| Parmotrema pilosum | 4 | MAF-Lich 7656 | Sierra de la Coronilla, Maldonado, URUGUAY | 250 | 34.21S, 54.38W | 23.3 | 11.7 | 1125 | 132.6 |  |  |  |  |  |  |  |  |  |
| Parmotrema pilosum | 4 | Elix 3553 (ANUC) | W of Connabarabran on road to Siding Springs, AUSTRALIA | 600 | 31.16S, 149.10E | 25 | 9.2 | 672 | 62.89 |  |  |  |  |  |  |  |  |  |
| Parmotrema pilosum | 4 | TRH sn | Natal, Cape prov., SOUTH AFRICA | 899 | 28.56S, 30.48E | 20.4 | 11.4 | 897 | 139.01 |  |  |  |  |  |  |  |  |  |
| Parmotrema pseudoreticulatum | 4 | MAF-Lich 7650 | Parque natural de los Alcornocales, entre Facinas y Barrios, Cádiz, SPAIN | 350 | 36.08N, 5.41W | 21.8 | 11.6 | 1065 | 130.9 |  |  |  |  |  |  |  |  |  |
| Parmotrema pseudoreticulatum | 4 | MAF-Lich 10278 | Santo antao do Tojal, Estremadura, PORTUGAL | 50 | 38.51N, 9.08W | 22.8 | 14.5 | 1024 | 192.29 |  |  |  |  |  |  |  |  |  |
| Parmotrema pseudoreticulatum | 4 | MAF-Lich 10288 | Addo Elephant National Park, Eastern Cape, SOUTH AFRICA | 31 | 33.33S, 25.40E | 22.5 | 13 | 610 | 78.72 |  |  |  |  |  |  |  |  |  |
| Parmotrema reticulatum | 4 | MAF-Lich 6067 | Castello do Mouros, Sintra, PORTUGAL | 400 | 38.47N, 9.23W | 18.2 | 11.7 | 803 | 188.49 |  |  |  |  |  |  |  |  |  |
| Parmotrema reticulatum | 4 | MAF-Lich 10267 | Las Mercedes, Tenerife, CANARY ISLAND | 900 | 28.30N, 16.11W | 19.1 | 13 | 518 | 102.45 |  |  |  |  |  |  |  |  |  |
| Parmotrema reticulatum | 4 | CANB (MAF 10286) | New South Wales, Brigdon Farm, AUSTRALIA | 30 | 36.33S, 150.03E | 22.3 | 5.8 | 584 | 70.63 |  |  |  |  |  |  |  |  |  |
| Parmotrema robustum | 4 | MAF-Lich 10166 | Nazaré, PORTUGAL | 70 | 39.35N, 9.03W | 17.8 | 10.7 | 1241 | 245.41 |  |  |  |  |  |  |  |  |  |
| Parmotrema robustum | 4 | MAF-Lich 7066 | Hervás, Caceres, SPAIN | 688 | 40.16N, 5.51W | 23.8 | 6.2 | 1138 | 105.28 |  |  |  |  |  |  |  |  |  |
| Parmotrema robustum | 4 | MAF-Lich 10166 | Castello do Mouro, Sintra, PORTUGAL | 427 | 38.47N, 9.23W | 18.2 | 11.7 | 803 | 188.49 |  |  |  |  |  |  |  |  |  |
| Parmotrema subcaperatum | 4 | HO 324283 | Grasstree hill, Tasmania, AUSTRALIA | 400 | 42.47S, 147.21E | 16.3 | 7.8 | 654 | 144.48 |  |  |  |  |  |  |  |  |  |
| Parmotrema subcaperatum | 4 | PERTH sn | Along south face at base of Devils slide, Porongurup National Park, AUSTRALIA | 583 | 34.40S, 117.50E | 23.9 | 13.1 | 889 | 113.23 |  |  |  |  |  |  |  |  |  |
| Parmotrema subcaperatum | 4 | MEL 5466 | Mt. Glorious, Queensland, AUSTRALIA | 672 | 27.19S, 152.45E | 22 | 10.6 | 927 | 123.94 |  |  |  |  |  |  |  |  |  |
| Parmotrema subtinctorium | 4 | GPGC 02-000696 | Goriganga Catchment, Uttaranchal, INDIA | 1400 | 29.60N, 80.20E | 21 | 6 | 1301 | 210.95 |  |  |  |  |  |  |  |  |  |
| Parmotrema subtinctorium | 4 | LWU 669 | Kodaikanal, Palni hills, INDIA | 2100 | 11.24N, 76.43E | 28.5 | 21.7 | 1398 | 159.59 |  |  |  |  |  |  |  |  |  |
| Parmotrema subtinctorium | 4 | ANUC 2633 | Atherton, Queensland, AUSTRALIA | 758 | 17.15S, 145.28E | 27 | 15 | 950 | 81.97 |  |  |  |  |  |  |  |  |  |
| Parmotrema tinctorum | 4 | MAF-Lich 10163 | 13 km SSE batemats bay, Melville Point, AUSTRALIA | 1 | 35.72S, 150.18E | 19.7 | 13.6 | 1038 | 214.62 |  |  |  |  |  |  |  |  |  |
| Parmotrema tinctorum | 4 | MAF-Lich 10503 | New South Wales, Brigadoon form, AUSTRALIA | 30 | 36.33S, 150.03E | 22.3 | 5.8 | 584 | 70.63 |  |  |  |  |  |  |  |  |  |
| Parmotrema tinctorum | 4 | AWAS 2707 | Pithoragarh district, Uttaranchal, INDIA | 1350 | 29.35N, 80.11E | 21 | 6 | 1301 | 210.95 |  |  |  |  |  |  |  |  |  |
| Xanthoparmelia conspersa | 3 | MAF-Lich 6793 | Puebla de Sanabria, Zamora, SPAIN | 960 | 42.03N, 6.38W | 18.3 | 3 | 946 | 131.09 |  |  |  |  |  |  |  |  |  |
| Xanthoparmelia conspersa | 3 | MAF-Lich 14092 | Mataelpino, Madrid, SPAIN | 1080 | 40.44N, 3.56W | 19.9 | 3.4 | 725 | 96.7 |  |  |  |  |  |  |  |  |  |
| Xanthoparmelia conspersa | 3 | MAF-Lich 10539 | Anguiano, La Rioja, SPAIN | 654 | 42.22N, 2.30W | 18.2 | 2.9 | 861 | 142.28 |  |  |  |  |  |  |  |  |  |
| Xanthoparmelia crespoae | 3 | MAF-Lich 7524 | New South Wales, Braidwood, AUSTRALIA | 600 | 35.26S, 149.48E | 19 | 5.4 | 760 | 84.35 |  |  |  |  |  |  |  |  |  |
| Xanthoparmelia crespoae | 3 | MAF-Lich 7440 | New South Wales, Braidwood, AUSTRALIA | 600 | 35.26S, 149.48E | 19 | 5.4 | 760 | 84.35 |  |  |  |  |  |  |  |  |  |
| Xanthoparmelia crespoae | 3 | MAF-Lich 7456 | New South Wales, Braidwood, AUSTRALIA | 600 | 35.26S, 149.48E | 19 | 5.4 | 760 | 84.35 |  |  |  |  |  |  |  |  |  |
| Xanthoparmelia delisei | 3 | MAF-Lich 7659 | Arroyo del Zape, Zamora, SPAIN | 690 | 41.28N, 5.47W | 23.6 | 4.2 | 443 | 39.48 |  |  |  |  |  |  |  |  |  |
| Xanthoparmelia delisei | 3 | MAF-Lich 9886 | Puerto de la Morcuera, Madrid, SPAIN | 1700 | 40.49N, 3.49W | 22.7 | 3.9 | 692 | 79.52 |  |  |  |  |  |  |  |  |  |
| Xanthoparmelia delisei | 3 | MAF-Lich 7659 | New South Wales, Braidwood, AUSTRALIA | 600 | 35.21S, 149.44E | 19 | 5.4 | 760 | 84.35 |  |  |  |  |  |  |  |  |  |
| Xanthoparmelia digitiformis | 3 | MAF-Lich 7525 | Australian Capital Territory, Brindabella Range, AUSTRALIA | 1644 | 35.20S, 148.43E | 17.3 | 2.9 | 777 | 118.52 |  |  |  |  |  |  |  |  |  |
| Xanthoparmelia digitiformis | 3 | MAF-Lich 7546 | Australian Capital Territory, Black Mountain Reserve, Canberra, AUSTRALIA | 750 | 35.16S, 149.05E | 20.3 | 5.8 | 584 | 75.63 |  |  |  |  |  |  |  |  |  |
| Xanthoparmelia digitiformis | 3 | MAF-Lich 7560 | Australian Capital Territory, Black Mountain Reserve, Canberra, AUSTRALIA | 750 | 35.16S, 149.05E | 20.3 | 5.8 | 584 | 75.63 |  |  |  |  |  |  |  |  |  |
| Xanthoparmelia glabrans | 3 | MAF-Lich 7665 | Australian capital territory, Brindabella Range, AUSTRALIA | 1644 | 35.20S, 148.43E | 17.3 | 2.9 | 777 | 118.52 |  |  |  |  |  |  |  |  |  |
| Xanthoparmelia glabrans | 3 | BRI 2877 | Girraween National Park, Queensland, AUSTRALIA | 1000 | 28.52S, 151.58E | 22.2 | 7.8 | 761 | 84.5 |  |  |  |  |  |  |  |  |  |
| Xanthoparmelia glabrans | 3 | MAF-Lich 9912 | Embid de la Rivera, Zaragoza, SPAIN | 480 | 41.25N, 1.35W | 23.3 | 5 | 434 | 44.6 |  |  |  |  |  |  |  |  |  |
| Xanthoparmelia huenana | 3 | GZU 46511 | Trail E of Swakopmund, NAMIBIA | 1 | 22.40S, 14.32E | 19.2 | 13.4 | 16 | 4.75 |  |  |  |  |  |  |  |  |  |
| Xanthoparmelia huenana | 3 | Jensen 6 (US) | Near Walvis bay, NAMIBIA | 20 | 22.59S, 14.32E | 18 | 13.5 | 18 | 4.69 |  |  |  |  |  |  |  |  |  |
| Xanthoparmelia huenana | 3 | Moisel 5170 (M; US) | 10 km E of Swakopmund, NAMIBIA | 20 | 22.39S, 14.32E | 19.2 | 13.4 | 16 | 4.75 |  |  |  |  |  |  |  |  |  |
| Xanthoparmelia isidiovagans | 3 | MAF-Lich 9956 | Torremocha, Guadalajara, SPAIN | 450 | 40.58N, 2.37W | 21.7 | 2.8 | 601 | 60.84 |  |  |  |  |  |  |  |  |  |
| Xanthoparmelia isidiovagans | 3 | 19612a | Tandir village, Eskisehir prov., Turkey | 1350 | 39.55N, 30.40E | 23.3 | 1.7 | 671 | 71.84 |  |  |  |  |  |  |  |  |  |
| Xanthoparmelia lithophila | 3 | MAF-Lich 6900 | New South Wales, Braidwood, AUSTRALIA | 600 | 35.26S, 149.48E | 19 | 5.4 | 760 | 84.35 |  |  |  |  |  |  |  |  |  |
| Xanthoparmelia lithophila | 3 | MAF-Lich 9661 | Morton National Park, AUSTRALIA | 750 | 35.21S 150.15E | 20.3 | 5.8 | 584 | 75.63 |  |  |  |  |  |  |  |  |  |
| Xanthoparmelia lithophila | 3 | MAF-Lich 10505 | New South Wales, Brigadoon farm, AUSTRALIA | 30 | 36.34S, 150.03E | 22.3 | 5.8 | 584 | 70.63 |  |  |  |  |  |  |  |  |  |
| Xanthoparmelia loxodes | 3 | MAF-Lich 7072 | Béjar, Salamanca, SPAIN | 1200 | 40.22N, 5.46W | 19.6 | 3 | 884 | 116.27 |  |  |  |  |  |  |  |  |  |
| Xanthoparmelia loxodes | 3 | MAF-Lich 6206 | Laguna de los Peces, Zamora, SPAIN | 1650 | 42.09N, 6,43W | 18.3 | 3 | 946 | 131.09 |  |  |  |  |  |  |  |  |  |
| Xanthoparmelia loxodes | 3 | MAF 6206 | Mataelpino, Madrid, SPAIN | 1080 | 40.44N, 3.56W | 19.9 | 3.4 | 725 | 96.7 |  |  |  |  |  |  |  |  |  |
| Xanthoparmelia mougeotii | 3 | MAF-Lich 9916 | Montenegro, La Rioja, SPAIN | 1250 | 42.05N, 2.45W | 17.5 | 1 | 548 | 88.96 |  |  |  |  |  |  |  |  |  |
| Xanthoparmelia mougeotii | 3 | G s.n. | Near Concepcion, ARGENTINA | 663 | 28.40S, 65.24W | 26.8 | 11.5 | 338 | 27.39 |  |  |  |  |  |  |  |  |  |
| Xanthoparmelia mougeotii | 3 | MAF-Lich 7472 | Australian Capital territory, Molonglo Gorge Reserve, AUSTRALIA | 550 | 35.20S, 149.15E | 20.6 | 6.3 | 610 | 72.44 |  |  |  |  |  |  |  |  |  |
| Xanthoparmelia murina | 3 | MAF-Lich 9915 | Australian Capital Territory, Molonglo Gorge Reserve, AUSTRALIA | 550 | 35.22S, 149.21E | 20.6 | 6.3 | 610 | 72.44 |  |  |  |  |  |  |  |  |  |
| Xanthoparmelia murina | 3 | MAF-Lich 7580 | Currowan state forest, 15km from Batemans bay, AUSTRALIA | 60 | 35.28S, 150.05E | 20.3 | 5.8 | 584 | 75.63 |  |  |  |  |  |  |  |  |  |
| Xanthoparmelia murina | 3 | MAF-Lich 7586 | Currowan state forest, 15km from Batemans bay, AUSTRALIA | 60 | 35.28S, 150.05E | 20.3 | 5.8 | 584 | 75.63 |  |  |  |  |  |  |  |  |  |
| Xanthoparmelia norcapnhodes | 3 | MAF-Lich 7532 | Australian Capital Territory, Molonglo Gorge Reserve, AUSTRALIA | 550 | 35.22S, 149.21E | 20.6 | 6.3 | 610 | 72.44 |  |  |  |  |  |  |  |  |  |
| Xanthoparmelia norcapnhodes | 3 | MAF-Lich 7401 | Australian Capital territory, 4km S of Tharwa, AUSTRALIA | 650 | 35.34S, 149.05E | 20.6 | 6.3 | 610 | 72.44 |  |  |  |  |  |  |  |  |  |
| Xanthoparmelia norcapnhodes | 3 | Elix 1030 (ANUC) | W slopes of Black Mnt, Canberra, AUSTRALIA | 500 | 30.32S, 152.18E | 25 | 9.2 | 672 | 62.89 |  |  |  |  |  |  |  |  |  |
| Xanthoparmelia notata | 3 | Elix 42648 (MAF-Lich 7521) | Australian Capital Territory, Aranda Bushland, AUSTRALIA | 680 | 35.15S, 149.04E | 20.3 | 5.8 | 584 | 75.63 |  |  |  |  |  |  |  |  |  |
| Xanthoparmelia notata | 3 | MAF-Lich 7429 | New South Wales, Braidwood, AUSTRALIA | 600 | 35.21S, 149.44E | 19 | 5.4 | 760 | 84.35 |  |  |  |  |  |  |  |  |  |
| Xanthoparmelia notata | 3 | MAF-Lich 7488 | Australian Capital Territory, Molonglo Gorge Reserve, AUSTRALIA | 550 | 35.20S, 149.15E | 20.6 | 6.3 | 610 | 72.44 |  |  |  |  |  |  |  |  |  |
| Xanthoparmelia pokornyii | 3 | MAF-Lich 9908 | Embid de la Ribera, Zaragoza, SPAIN | 480 | 41.25N, 1.35W | 23.3 | 5 | 434 | 44.6 |  |  |  |  |  |  |  |  |  |
| Xanthoparmelia pokornyi | 3 | MAF-Lich 6052 | Zaorejas, Guadalajara, SPAIN | 1250 | 40.44N, 2.11W | 22 | 3.5 | 668 | 73.76 |  |  |  |  |  |  |  |  |  |
| Xanthoparmelia pokornyi | 3 | MAF-Lich 11395 | Zaorejas, Guadalajara, SPAIN | 1250 | 40.44N, 2.11W | 22 | 3.5 | 668 | 73.76 |  |  |  |  |  |  |  |  |  |
| Xanthoparmelia protomatrae | 3 | MAF-Lich 6216 | Puebla de Sanabria, Zamora, SPAIN | 960 | 42.03N, 6.38W | 18.3 | 3 | 946 | 131.09 |  |  |  |  |  |  |  |  |  |
| Xanthoparmelia protomatrae | 3 | MAF-Lich 9847 | Port de la selva, Gerona, SPAIN | 30 | 42.19N, 3.08E | 23.3 | 7.2 | 582 | 72.08 |  |  |  |  |  |  |  |  |  |
| Xanthoparmelia protomatrae | 3 | MAF-Lich 3777 | Between Berrueco and Berzosa del Lozoya, Madrid, SPAIN | 900 | 40.56N, 3,33W | 21.1 | 3.2 | 650 | 75.84 |  |  |  |  |  |  |  |  |  |
| Xanthoparmelia pulla | 3 | MAF-Lich 6794 | Mataelpino, Madrid, SPAIN | 1080 | 40.44N, 3.56W | 19.9 | 3.4 | 725 | 96.7 |  |  |  |  |  |  |  |  |  |
| Xanthoparmelia pulla | 3 | MAF-Lich 12517 | Ezcaray, La Rioja, SPAIN | 1780 | 42.03N, 3.03W | 17.5 | 1 | 548 | 88.96 |  |  |  |  |  |  |  |  |  |
| Xanthoparmelia pulla | 3 | MAF-Lich 7058 | La Alberca, Salamanca, SPAIN | 1200 | 40.29N, 6.07W | 21.7 | 3.8 | 551 | 61.63 |  |  |  |  |  |  |  |  |  |
| Xanthoparmelia pulloides | 3 | MAF-Lich 6784 | Carretera Zamora-Almaráz, Zamora, SPAIN | 700 | 41.28N, 5.47W | 21.9 | 4.3 | 415 | 46.43 |  |  |  |  |  |  |  |  |  |
| Xanthoparmelia pulloides | 3 | Galun 128 | Upper Jordan valley, Korazim, Oregón, USA | 1300 | 43.01N, 116.54W | 20.3 | -3.6 | 289 | 35.23 |  |  |  |  |  |  |  |  |  |
| Xanthoparmelia pulloides | 3 | Culberson & Culberson 16099A | Between Tejina and Valle-Guerra, ca. 2 km from the sea, Tenerife, CANARY ISLAND | 200 | 28.32N, 16.21W | 22 | 15.4 | 450 | 84.01 |  |  |  |  |  |  |  |  |  |
| Xanthoparmelia scotophylla | 3 | Elix 30650 | Mount Remarkable National Park, Mambray Creek Section, AUSTRALIA | 50 | 32.50S, 137.58E | 25.3 | 11.1 | 229 | 20.62 |  |  |  |  |  |  |  |  |  |
| Xanthoparmelia scotophylla | 3 | Elix 17795 | Warren Gorge, 18.5 km N of Quorn, AUSTRALIA | 100 | 32.01S, 138.01E | 27.2 | 10.3 | 247 | 20.38 |  |  |  |  |  |  |  |  |  |
| Xanthoparmelia scotophylla | 3 | Elix 3907 (MEL) | Trail to Pigeon House Mtn, 15 km W of Ulladulla, AUSTRALIA | 90 | 35.21S, 150.23E | 22.9 | 8.6 | 433 | 49.96 |  |  |  |  |  |  |  |  |  |
| Xanthoparmelia semiviridis | 3 | MAF-Lich 6876 | New South Wales, Bredbo, AUSTRALIA | 760 | 35.57S, 149.08E | 16.5 | 8.3 | 648 | 102.2 |  |  |  |  |  |  |  |  |  |
| Xanthoparmelia semiviridis | 3 | Elix 10883 (CANB) | New South Wales, 2 Km N of Bredbo, AUSTRALIA | 720 | 35.57S, 149.08E | 20.3 | 5.8 | 584 | 75.63 |  |  |  |  |  |  |  |  |  |
| Xanthoparmelia semiviridis | 3 | Elix 9364 (ANUC) | 8 km S of Black Hill Township, AUSTRALIA | 75 | 34.41S, 139.27E | 21.7 | 8.6 | 410 | 46.08 |  |  |  |  |  |  |  |  |  |
| Xanthoparmelia stenophylla | 3 | MAF-Lich 9917 | Torremocha, Guadalajara, SPAIN | 450 | 40.58N, 2.37W | 21.7 | 2.8 | 601 | 60.84 |  |  |  |  |  |  |  |  |  |
| Xanthoparmelia stenophylla | 3 | MAF-Lich 9846 | Mataelpino, Madrid, SPAIN | 1080 | 40.44N, 3.56W | 19.9 | 3.4 | 725 | 96.7 |  |  |  |  |  |  |  |  |  |
| Xanthoparmelia stenophylla | 3 | MAF-Lich 10549 | Between Anguiano and Mansilla, La Rioja, SPAIN | 910 | 42.10N, 2.52W | 18.2 | 2.9 | 861 | 142.28 |  |  |  |  |  |  |  |  |  |
| Xanthoparmelia subincerta | 3 | MAF-Lich 7494 | Australian capital Territory, Molonglo Gorge Reserve, AUSTRALIA | 550 | 35.22S, 149.21E | 20.6 | 6.3 | 610 | 72.44 |  |  |  |  |  |  |  |  |  |
| Xanthoparmelia subincerta | 3 | Elix 21806 (ANUC) | Kowen forest, 16 km E of Canberra, AUSTRALIA | 640 | 35.19S, 149.14E | 20.3 | 5.8 | 584 | 75.63 |  |  |  |  |  |  |  |  |  |
| Xanthoparmelia subincerta | 3 | COLO L49062 | E of Cooma on road to Numeralla, AUSTRALIA | 880 | 36.13S, 149.07E | 19 | 5.4 | 760 | 84.35 |  |  |  |  |  |  |  |  |  |
| Xanthoparmelia subspodochroa | 3 | MAF-Lich 7463 | New South Wales, Braidwood, AUSTRALIA | 600 | 35.26S, 149.48E | 19 | 5.4 | 760 | 84.35 |  |  |  |  |  |  |  |  |  |
| Xanthoparmelia subspodochroa | 3 | MAF-Lich 7459 | New South Wales, Braidwood, AUSTRALIA | 600 | 35.26S, 149.48E | 19 | 5.4 | 760 | 84.35 |  |  |  |  |  |  |  |  |  |
| Xanthoparmelia subspodochroa | 3 | Verdon 1699 (CBG) | Naas Ck, 57 km of Canberra, AUSTRALIA | 660 | 35.07S, 149.17E | 20.3 | 5.8 | 584 | 75.63 |  |  |  |  |  |  |  |  |  |
| Xanthoparmelia subprolixa | 3 | MAF-Lich 7667 | Australian Capital territory, Brindabella Range, AUSTRALIA | 1644 | 35.20S, 148.43E | 17.3 | 2.9 | 777 | 118.52 |  |  |  |  |  |  |  |  |  |
| Xanthoparmelia subprolixa | 3 | MAF-Lich 7484 | Australian Capital Territory, Molonglo Gorge reserve, AUSTRALIA | 550 | 35.22S, 149.21E | 20.6 | 6.3 | 610 | 72.44 |  |  |  |  |  |  |  |  |  |
| Xanthoparmelia subprolixa | 3 | MAF-Lich 7424 | New South Wales, Braidwood, AUSTRALIA | 600 | 35.21S, 149.44E | 20.6 | 6.3 | 610 | 72.44 |  |  |  |  |  |  |  |  |  |
| Xanthoparmelia tegeta | 3 | MAF-Lich 7523 | Australian Capital Territory, Brindabella Range, AUSTRALIA | 1644 | 35.20S, 148.43E | 17.3 | 2.9 | 777 | 118.52 |  |  |  |  |  |  |  |  |  |
| Xanthoparmelia tegeta | 3 | Elix 1718 | Diggers Creek, Kosciusko National Park, AUSTRALIA | 1100 | 36.03S, 148.30E | 19.7 | 9.5 | 653 | 104.6 |  |  |  |  |  |  |  |  |  |
| Xanthoparmelia tegeta | 3 | Streimann 9727 (CBG) | Bimberi Peak, Bimberi, AUSTRALIA | 1330 | 35.38S, 148.46E | 19 | 5.4 | 760 | 84.35 |  |  |  |  |  |  |  |  |  |
| Xanthoparmelia tinctina | 3 | BCN-13862 | Alt Empordá, Tarragona, SPAIN | 20 | 41.07N, 1.20W | 24.3 | 8.2 | 556 | 74.99 |  |  |  |  |  |  |  |  |  |
| Xanthoparmelia tinctina | 3 | MAF-Lich 6070 | Montejo de la Sierra, Madrid, SPAIN | 1100 | 41.03N, 3.31W | 19.9 | 3.4 | 725 | 96.7 |  |  |  |  |  |  |  |  |  |
| Xanthoparmelia tinctina | 3 | MAF-Lich 9883 | Mataelpino, Madrid, SPAIN | 1080 | 40.44N, 3.56W | 19.9 | 3.4 | 725 | 96.7 |  |  |  |  |  |  |  |  |  |
| Xanthoparmelia transvaalensis | 3 | MAF-Lich 9841 | Embid de la Ribera, Zaragoza, SPAIN | 480 | 41.25N, 1.35W | 23.3 | 5 | 434 | 44.6 |  |  |  |  |  |  |  |  |  |
| Xanthoparmelia transvaalensis | 3 | Hale 72038 | Long Tom Pass, Farm De Kuiten, Lydenburg, Transvaal, SOUTH AFRICA | 1740 | 25.10S, 30.37E | 20.4 | 9.9 | 730 | 106.4 |  |  |  |  |  |  |  |  |  |
| Xanthoparmelia verrucigera | 3 | MAF-Lich 9920 | Vilajuiga, Gerona, SPAIN | 25 | 42.19N, 3.05W | 23.3 | 7.2 | 582 | 72.08 |  |  |  |  |  |  |  |  |  |
| Xanthoparmelia verrucigera | 3 | MAF-Lich 10180 | Embid de la Ribera, Zaragoza, SPAIN | 480 | 41.25N, 1.35W | 23.3 | 5 | 434 | 44.6 |  |  |  |  |  |  |  |  |  |
| Xanthoparmelia verrucigera | 3 | MAF-Lich 15301 | Braganza, PORTUGAL | 1040 | 41.53N, 6.56W | 21 | 4.8 | 901 | 109.49 |  |  |  |  |  |  |  |  |  |
| Xanthoparmelia vicentei | 3 | MAF-Lich 7248 | Béjar, Salamanca, SPAIN | 1200 | 40.22N, 5.46W | 19.6 | 3 | 884 | 116.27 |  |  |  |  |  |  |  |  |  |
| Xanthoparmelia vicentei | 3 | MAF-Lich 9936 | Arroyo del zape, Zamora, SPAIN | 669 | 41.28N, 5.47W | 23.6 | 4.2 | 443 | 39.48 |  |  |  |  |  |  |  |  |  |
| Xanthoparmelia vicentei | 3 | MAF-Lich 9954 | El Castanar, Salamanca, SPAIN | 644 | 40.29N, 5.59W | 26.7 | 6.1 | 728 | 61.46 Species |  | HERBARIUM | Locality | Altitude (m) | Latitude | TempMax C(Avr) | TempMin. C(Avr.) | Precipitation (mm) | Index of Emberger (Q=100/(Tmax-Tmin) |
| Bulbothrix apophysata | 2 | Lucking 16650b (L) | San Pedro, San José, COSTA RICA | 1100 | 9.57N, 84.02W | 21.4 | 19.0 | 1944 | 384.55 |  |  |  |  |  |  |  |  |  |
| Bulbothrix apophysata | 2 | US 1603 | La Vega, DOMINICAN REPUBLIC | 100 | 19.13N, 70.31W | 27 | 23.9 | 1418 | 232.38 |  |  |  |  |  |  |  |  |  |
| Bulbothrix apophysata | 2 | Hale, 18032 | La Vega, DOMINICAN REPUBLIC | 100 | 19.13N, 70.31W | 27 | 23.9 | 1418 | 232.38 |  |  |  |  |  |  |  |  |  |
| Bulbothrix coronata | 2 | MAF-Lich 13987 | Coegmansk loof near Ashton, Cape Prov., SOUTH AFRICA | 230 | 33.48 S, 20.06 E | 24.8 | 11.7 | 784 | 92.12 |  |  |  |  |  |  |  |  |  |
| Bulbothrix coronata | 2 | P 4192 | Balansa, PARAGUAY | 231 | 22.07S, 61.55W | 28.6 | 20 | 1090 | 111.69 |  |  |  |  |  |  |  |  |  |
| Bulbothrix coronata | 2 | LD sn | Mato Grosso, BRAZIL | 384 | 12.39S, 55.57W | 26.7 | 21.9 | 1577 | 173.17 |  |  |  |  |  |  |  |  |  |
| Bulbothrix decurtata | 2 | MAF-Lich 13988 | Hottentots Holland Nature Reserve,Cape Prov., SOUTH AFRICA | 1000 | 34.03 S, 19.01E | 20.2 | 4.0 | 1112 | 130.82 |  |  |  |  |  |  |  |  |  |
| Bulbothrix decurtata | 2 | 19Q | Paarl Mountain, Gordon's Way, Cape Prov., SOUTH AFRICA | 530 | 33.44S, 18.56E | 20.5 | 12.3 | 1008 | 161.5 |  |  |  |  |  |  |  |  |  |
| Bulbothrix decurtata | 2 | 1F | Harverd Poter National Garden, E. of Betty's Bay , SOUTH AFRICA | 61 | 34.20S, 18.55E | 20.5 | 12.3 | 1008 | 161.5 |  |  |  |  |  |  |  |  |  |
| Bulbothrix goebelii | 2 | MAF-Lich 13985 | Kristenbosch Botanic Garden, Cape Prov., SOUTH AFRICA | 140 | 33.50S, 18.25E | 21.0 | 12.0 | 936 | 125 |  |  |  |  |  |  |  |  |  |
| Bulbothrix goebelii | 2 | 36959Elix | Edmund Kennedy National Park, Queensland, AUSTRALIA | 2 | 18.12S, 146.00E | 27.5 | 19.4 | 1217 | 171 |  |  |  |  |  |  |  |  |  |
| Bulbothrix goebelii | 2 | ASSAM 2834 | Phek, Nagaland, INDIA | 1300 | 25.39N, 94.29E | 28.6 | 18.3 | 3163 | 346.15 |  |  |  |  |  |  |  |  |  |
| Bulbothrix meizospora | 2 | GPGC 02-000786 | Goriganga Catchment, Uttaranchal, INDIA | 1700 | 29.60N, 80.20E | 21 | 6 | 1301 | 210.95 |  |  |  |  |  |  |  |  |  |
| Bulbothrix meizospora | 2 | LWU 67288 | Kalimpong, Darjeeling, W. Bengal, INDIA | 1500 | 27.04N, 88.28E | 16.7 | 5.0 | 3030 | 857.13 |  |  |  |  |  |  |  |  |  |
| Bulbothrix meizospora | 2 | LWG sn | Shimla, Himachal Pradesh, INDIA | 2100 | 31.06N, 77.10E | 19.4 | 5.3 | 1577 | 306.21 |  |  |  |  |  |  |  |  |  |
| Bulbothrix setschwanensis | 2 | MAF-Lich 10212 | Chu Xiong, Chu Xiong County, Yunnan, CHINA | 2200 | 24.59N, 101.26E | 22.2 | 10.0 | 821 | 97.32 |  |  |  |  |  |  |  |  |  |
| Bulbothrix setschwanensis | 2 | LWG sn | Dalhousie, Himachal Pradesh, INDIA | 1827 | 32.3N, 75.58E | 19.4 | 5.3 | 1577 | 306.21 |  |  |  |  |  |  |  |  |  |
| Bulbothrix setschwanensis | 2 | LWG 97559 | Moreh, Manipur, INDIA | 450 | 24.49N, 93.53E | 28.6 | 18.3 | 3163 | 346.15 |  |  |  |  |  |  |  |  |  |
| Everniastrum cirrhatum | 1 | Trest 149 | Cerro de la Muerte, Pérez Zeledón, San José, COSTA RICA | 3300 | 9.56N, 83.75W | 20 | 8 | 2061 | 261.54 |  |  |  |  |  |  |  |  |  |
| Everniastrum cirrhatum | 1 | MAF 10374 | Jian Chuan County, Yunnan, CHINA | 3980 | 26.31N, 99.43E | 19.2 | 7.6 | 963 | 140.45 |  |  |  |  |  |  |  |  |  |
| Everniastrum cirrhatum | 1 | MAF-Lich 13976 | Parque Nacional de Huascarán, Quebrada Paron PERU | 3860 | 9.50S, 77.50W | 13 | 9.5 | 724 | 181 |  |  |  |  |  |  |  |  |  |
| Everniastrum lipidiferum | 1 | MAF-Lich13966 | Parque Nacional de Huascarán, Quebrada Cojup, PERU | 3900 | 9.50S, 77.50W | 13 | 9.5 | 724 | 181 |  |  |  |  |  |  |  |  |  |
| Everniastrum lipidiferum | 1 | DUKE 20064 | El Sumidero, Chiapas, MEXICO | 1439 | 16.15N, 92.18W | 16.2 | 12.1 | 1138 | 307.73 |  |  |  |  |  |  |  |  |  |
| Everniastrum lipidiferum | 1 | US 44514 | End of Laguna negra, Merida, VENEZUELA | 3930 | 8.34N, 71.00W | 22.8 | 21.4 | 1796 | 294.29 |  |  |  |  |  |  |  |  |  |
| Everniastrum nepalense | 1 | GPGC 02-000924 | Lamaghar, Uttaranchal, INDIA | 1600 | 29.60N, 80.20E | 21 | 6 | 1301 | 210.95 |  |  |  |  |  |  |  |  |  |
| Everniastrum nepalense | 1 | AWAS 1419 | Jakhu top, Shimla, Himachal Pradesh, INDIA | 2400 | 31.16N, 77.10E | 19.4 | 5.3 | 1577 | 306.21 |  |  |  |  |  |  |  |  |  |
| Everniastrum nepalense | 1 | 1675 Kurokawa | Chiang Mai Prov., THAILAND | 500 | 18.42N, 98.47E | 30 | 22.8 | 1288 | 108.16 |  |  |  |  |  |  |  |  |  |
| Everniastrum rhizodendroideum | 1 | ABL 55665 | Jian Chuan County., Yunnan, CHINA | 3900 | 26.37N, 99.43E | 19.2 | 7.6 | 963 | 140.45 |  |  |  |  |  |  |  |  |  |
| Everniastrum rhizodendroideum | 1 | HAMH-L 762 | Xizang, TIBET | 4100 | 31.55N, 88.21E | 11.7 | 5.3 | 1012 | 143.99 |  |  |  |  |  |  |  |  |  |
| Everniastrum rhizodendroideum | 1 | B 131 | Namche Bazar, Khumbhu Himal, NEPAL | 3440 | 27.48N, 86.42E | 24.7 | 10.3 | 1427 | 166.07 |  |  |  |  |  |  |  |  |  |
| Everniastrum sorocheilum | 1 | MAF-Lich 10375 | Jian Chuan County, Yunnan, CHINA | 3980 | 26.37N, 99.43E | 19.2 | 7.6 | 963 | 140.45 |  |  |  |  |  |  |  |  |  |
| Everniastrum sorocheilum | 1 | US 44555 | End of Laguna negra, Sierra Nevada, Mérida, VENEZUELA | 3930 | 8.34N, 71.00W | 22.8 | 21.4 | 1796 | 294.29 |  |  |  |  |  |  |  |  |  |
| Everniastrum sorocheilum | 1 | LWG 85879A | Chamoli district, Uttaranchal, INDIA | 3150 | 30.25N, 79.12E | 21 | 6 | 1301 | 210.95 |  |  |  |  |  |  |  |  |  |
| Everniastrum vexans | 1 | ABL56597 | Yunlong Co., Yunnan, CHINA | 2500 | 25.45N, 99.06E | 20.9 | 10.0 | 1056 | 148.23 |  |  |  |  |  |  |  |  |  |
| Everniastrum vexans | 1 | LWU 8469 | Idukki district, Kerala, INDIA | 1200 | 9.5N, 76.59E | 28.3 | 25.6 | 1696 | 357.53 |  |  |  |  |  |  |  |  |  |
| Everniastrum vexans | 1 | LWU 67281 | Kalimpong, Darjeeling district, W. Bengal, INDIA | 1600 | 27.04N, 88.28E | 16.7 | 5.0 | 3030 | 857.13 |  |  |  |  |  |  |  |  |  |
| Hypotrachyna adducta | 2 | MAF-Lich 10206 | San Jian Bin Liv area, Jian Chuan County, Yunnan, CHINA | 2448 | 26.39N, 99.47E | 19.2 | 7.6 | 963 | 140.45 |  |  |  |  |  |  |  |  |  |
| Hypotrachyna adducta | 2 | MAF-Lich 10378 | Yunlong County, Yunnan, CHINA | 2500 | 25.45N, 99.06E | 20.9 | 10.0 | 1056 | 148.23 |  |  |  |  |  |  |  |  |  |
| Hypotrachyna adducta | 2 | MAF-Lich 15400 | Namachi, Sikkim, INDIA | 1800 | 27.10N, 88.20E | 16.7 | 5.0 | 3030 | 857.13 |  |  |  |  |  |  |  |  |  |
| Hypotrachyna aff brevirhiza | 2 | MAF-Lich 10376 | Jian Chuan County, Yunnan, CHINA | 2490 | 26.21N, 99.50E | 19.2 | 7.6 | 963 | 140.45 |  |  |  |  |  |  |  |  |  |
| Hypotrachyna aff immaculata | 1 | MAF-Lich 10413 | Chu Xiong County, Yunnan, CHINA | 2200 | 24.59N, 101.26E | 22.2 | 10.0 | 821 | 97.32 |  |  |  |  |  |  |  |  |  |
| Hypotrachyna aff. taylorensis | 1 | MAF-Lich 10409 | Las Mercedes,Tenerife, Canary Island, SPAIN | 1200 | 28.32N, 16.12W | 19.1 | 13 | 518 | 102.45 |  |  |  |  |  |  |  |  |  |
| Hypotrachyna booralensis | 1 | MAF-Lich 13969 | Capricoru coast, Queesland, AUSTRALIA | 10 | 23.12S, 150.45E | 25.7 | 17.8 | 1258 | 140.24 |  |  |  |  |  |  |  |  |  |
| Hypotrachyna booralensis | 1 | MEL 16764 | New South Wales, west of Booral, AUSTRALIA | 67 | 32.28S, 152.00E | 25 | 9.2 | 672 | 62.89 |  |  |  |  |  |  |  |  |  |
| Hypotrachyna ciliata | 2 | MAF-Lich 10185 | Shibado Shao park, Jian Chuan County, Yunnan, CHINA | 2448 | 26.21N, 99.50E | 19.2 | 7.6 | 963 | 140.45 |  |  |  |  |  |  |  |  |  |
| Hypotrachyna ciliata | 2 | Wang 4975 | Mt. Yulongshan, Yunnan, CHINA | 2700 | 26.46N, 100E | 19.2 | 7.6 | 963 | 140.45 |  |  |  |  |  |  |  |  |  |
| Hypotrachyna ciliata | 2 | Wang 5523 | Zhongdian, Yunnan, CHINA | 3279 | 27.48N, 99.42E | 26.1 | 4.2 | 1281 | 136.83 |  |  |  |  |  |  |  |  |  |
| Hypotrachyna costaricensis | 2 | MAF-Lich 10211 | Volcán Arenal, COSTA RICA | 500 | 10.48N, 84.85W | 25 | 17 | 2669 | 269.9 |  |  |  |  |  |  |  |  |  |
| Hypotrachyna costaricensis | 2 | 57 sergio | Sierra Maestra, Gramma, CUBA | 1700 | 20N, 77.49W | 27.5 | 22.2 | 1420 | 182.03 |  |  |  |  |  |  |  |  |  |
| Hypotrachyna costaricensis | 2 | MAF-Lich 2552 | Sierra Portuguessa, Lara Prov., VENEZUELA | 1900 | 10.08N, 69.52W | 22.8 | 21.4 | 1796 | 294.29 |  |  |  |  |  |  |  |  |  |
| Hypotrachyna crenata | 2 | MAF-Lich 10377 | Heqing County,Yunnan, CHINA | 2400 | 26.13N, 100.09E | 19.2 | 7.6 | 963 | 140.45 |  |  |  |  |  |  |  |  |  |
| Hypotrachyna crenata | 2 | LWG sn | Shillong, Meghalaya, INDIA | 900 | 25.34N, 91.52E | 28.6 | 18.3 | 3163 | 346.15 |  |  |  |  |  |  |  |  |  |
| Hypotrachyna crenata | 2 | LWU 6754 | Tiger hill, Darjeeling, W. Bengal, INDIA | 2550 | 26.59N, 88.17E | 16.7 | 5.0 | 3030 | 857.13 |  |  |  |  |  |  |  |  |  |
| Hypotrachyna endochlora | 1 | MAF-Lich 10178 | Crinanwood, Kintyre, Scotland, GREAT BRITAIN | 10 | 56.05N, 5.33W | 16.4 | 7 | 869 | 247.3 |  |  |  |  |  |  |  |  |  |
| Hypotrachyna endochlora | 1 | MAF-Lich 10379 | Las Mercedes, Tenerife, CANARY ISLAND | 900 | 28.30N, 16.11W | 19.1 | 13 | 518 | 102.45 |  |  |  |  |  |  |  |  |  |
| Hypotrachyna endochlora | 1 | MSC 3509 | La Vega, DOMINICAN REPUBLIC | 100 | 19.13N, 70.31W | 27 | 23.9 | 1418 | 232.38 |  |  |  |  |  |  |  |  |  |
| Hypotrchyna exsecta | 1 | MAF 10380 | Lunan County, Yunnan CHINA | 1909 | 24.48N, 103.17E | 20.8 | 8.1 | 1039 | 153.14 |  |  |  |  |  |  |  |  |  |
| Hypotrachyna exsecta | 1 | LWG 18400 | Berinag, Pithoragarh district, Uttaranchal, INDIA | 1900 | 29.35N, 80.11E | 21 | 6 | 1301 | 210.95 |  |  |  |  |  |  |  |  |  |
| Hypotrachyna exsecta | 1 | LWU 71363 | Avlanche, Nilgiri hills, Tamil Nadu, INDIA | 2100 | 11.24N, 76.41E | 28.5 | 21.7 | 1398 | 159.59 |  |  |  |  |  |  |  |  |  |
| Hypotrachyna flexilis | 2 | MAF-Lich 13975 | Above Lachung towards Yumthang, Sikkim, INDIA | 3000 | 27.42N, 88.45E | 16.7 | 5.0 | 3030 | 857.13 |  |  |  |  |  |  |  |  |  |
| Hypotrachyna flexilis | 2 | LWU 67232 | Kalimpong, Darjeeling district, W. Bengal, INDIA | 1500 | 27.04N, 88.28E | 16.7 | 5.0 | 3037 | 857.13 |  |  |  |  |  |  |  |  |  |
| Hypotrachyna flexilis | 2 | LWU 7697 | Dailekh, Bheri zone, NEPAL | 2100 | 28.46N, 81.51E | 25 | 12.5 | 3388 | 397.63 |  |  |  |  |  |  |  |  |  |
| Hypotrachyna imbricatula | 1 | MAF-Lich 13990 | Hottentots Holland Nature Reserve, Cape Prov., SOUTH AFRICA | 1000 | 34.03 S, 19.01E | 20.2 | 4.0 | 1112 | 130.82 |  |  |  |  |  |  |  |  |  |
| Hypotrachyna imbricatula | 1 | MICH sn | Itaperica, Sao Paulo, BRAZIL | 10 | 12.53S, 38.40W | 26.9 | 22.5 | 1069 | 119.12 |  |  |  |  |  |  |  |  |  |
| Hypotrachyna imbricatula | 1 | BRI 141 | Lamington National Park, moon light carg, Queensland, AUSTRALIA | 519 | 28.14S, 153.07E | 22.0 | 10.6 | 927 | 123.94 |  |  |  |  |  |  |  |  |  |
| Hypotrachyna immaculata | 1 | MAF-Lich 7462 | Morton National Park, Pidgeon House Mountain, AUSTRALIA | 750 | 34.59S 150.25E | 17 | 9.1 | 1511 | 286.54 |  |  |  |  |  |  |  |  |  |
| Hypotrachyna immaculata | 1 | MAF-Lich 10383 | Jian Chuan County, Yunnan, CHINA | 2450 | 26.21N, 99.5E | 19.2 | 7.6 | 963 | 140.45 |  |  |  |  |  |  |  |  |  |
| Hypotrachyna immaculata | 1 | 36486Elix | New South Wales, Oxley wild rivers National Park, AUSTRALIA | 945 | 30.40S, 151.43E | 22.0 | 10.6 | 927 | 123.94 |  |  |  |  |  |  |  |  |  |
| Hypotrachyna incognita | 2 | MAF-Lich 10385 | Dali County, Yunnan, CHINA | 3500 | 25.41N, 100.06E | 20.9 | 10.0 | 1056 | 148.23 |  |  |  |  |  |  |  |  |  |
| Hypotrachyna incognita | 2 | MAF-Lich 10384 | Jian Chuan County, Yunnan, CHINA | 2450 | 26.21N, 99.5E | 19.2 | 7.6 | 963 | 140.45 |  |  |  |  |  |  |  |  |  |
| Hypotrachyna incognita | 2 | AWAS 7896 | Elephant fall, Shillong, Meghalaya, INDIA | 1600 | 25.30N, 91.62E | 28.6 | 18.3 | 3163 | 346.15 |  |  |  |  |  |  |  |  |  |
| Hypotrachyna infirma | 2 | MAF-Lich 10210 | Yu Long Shan, Lijian County, Jade Dragon Snow Mountain, Yunnan, CHINA | 2700 | 26.46N, 100E | 19.2 | 7.6 | 963 | 140.45 |  |  |  |  |  |  |  |  |  |
| Hypotrachyna infirma | 2 | MAF-Lich 10386 | Yunlong County, Yunnan, CHINA | 2410 | 25.45N, 99.06E | 20.9 | 10.0 | 1056 | 148.23 |  |  |  |  |  |  |  |  |  |
| Hypotrachyna infirma | 2 | LWU 67312 | Kalimpong, Darjeeling district, W. Bengal, INDIA | 1500 | 27N, 88.28E | 16.7 | 5.0 | 3037 | 857.13 |  |  |  |  |  |  |  |  |  |
| Hypotrachyna koyaensis | 2 | MAF-Lich 10388 | Yunlong County, Yunnan, CHINA | 2500 | 25.45N, 99.06E | 20.9 | 10.0 | 1056 | 148.23 |  |  |  |  |  |  |  |  |  |
| Hypotrachyna koyaensis | 2 | AWAS 3924 | Kurseong, Darjeeling, district, W. Bengal, INDIA | 1650 | 26.52N, 88.16E | 16.7 | 5.0 | 3037 | 857.13 |  |  |  |  |  |  |  |  |  |
| Hypotrachyna koyaensis | 2 | LWU 76254 | Dailekh, Bheri zone, NEPAL | 2100 | 28.46N, 81.51E | 25 | 12.5 | 3388 | 397.63 |  |  |  |  |  |  |  |  |  |
| Hypotrachyna laevigata | 1 | MAF-Lich 10177 | Tayvallich, Argyll and Bute, Scotland, GREAT BRITAIN | 13 | 56.01N, 5.37W | 16.4 | 7 | 869 | 247.3 |  |  |  |  |  |  |  |  |  |
| Hypotrachyna laevigata | 1 | MAF-Lich 6975 | Las Mercedes, Tenerife, CANARY ISLAND | 900 | 28.30N, 16.11W | 19.1 | 13 | 518 | 102.45 |  |  |  |  |  |  |  |  |  |
| Hypotrachyna laevigata | 1 | ANUC 40144 | Arthur-Pieman, protected area, Tasmania, AUSTRALIA | 335 | 41.30S, 145E | 18.2 | 7.4 | 742 | 120.23 |  |  |  |  |  |  |  |  |  |
| Hypotrachyna neodissecta | 1 | MAF-Lich 13986 | Kristenbosch Botanic Garden, Cape Prov., SOUTH AFRICA | 140 | 33.50S, 18.25E | 21.0 | 12.0 | 936 | 125 |  |  |  |  |  |  |  |  |  |
| Hypotrachyna neodissecta | 1 | MAF-Lich 15416 | Mount Elgan Forest, Bukusu distt., Western Prov., KENYA | 2537 | 0.56N, 34.38E | 20 | 17.2 | 1140 | 175.53 |  |  |  |  |  |  |  |  |  |
| Hypotrachyna neodissecta | 1 | MAF-Lich 15385 | Kodaikanal, Palni hills, INDIA | 2200 | 11.24N, 76.43E | 28.5 | 21.7 | 1398 | 159.59 |  |  |  |  |  |  |  |  |  |
| Hypotrachyna osseoalba | 1 | MAF-Lich 10390 | Chu Xiong County, Yunnan, CHINA | 2200 | 24.59N, 101.26E | 22.2 | 10.0 | 821 | 97.32 |  |  |  |  |  |  |  |  |  |
| Hypotrachyna osseoalba | 1 | MAF-Lich 10389 | Lunan County, Yunnan, CHINA | 1909 | 24.43N, 103.20E | 20.8 | 8.1 | 1039 | 153.14 |  |  |  |  |  |  |  |  |  |
| Hypotrachyna osseoalba | 1 | MAF-Lich 9663 | Morton National Park, Pidgeon House Mountain, AUSTRALIA | 750 | 34.59S 150.25E | 19.2 | 6.9 | 1511 | 228.18 |  |  |  |  |  |  |  |  |  |
| Hypotrachyna physcioides | 1 | MAF-Lich 10391 | Dali County,Yunnan, CHINA | 3150 | 25.41N, 100.06E | 20.9 | 10.0 | 1056 | 148.23 |  |  |  |  |  |  |  |  |  |
| Hypotrachyna physcioides | 1 | MAF-Lich 10412 | Jian Chuan County, Yunnan, CHINA | 3200 | 26.31N, 99.43E | 19.2 | 7.6 | 963 | 140.45 |  |  |  |  |  |  |  |  |  |
| Hypotrachyna physcioides | 1 | MAF-Lich 15404 | Pithoragarh district, Uttaranchal, INDIA | 1800 | 29.35N, 80.11E | 21 | 6 | 1301 | 210.95 |  |  |  |  |  |  |  |  |  |
| Hypotrachyna pseudosinuosa | 1 | MAF-Lich 10392 | Lunan County, Yunnan, CHINA | 1909 | 24.48N, 103.17E | 20.8 | 8.1 | 1039 | 153.14 |  |  |  |  |  |  |  |  |  |
| Hypotrachyna pseudosinuosa | 1 | MAF-Lich 10393 | Lunan County, Yunnan, CHINA | 1909 | 24.48N, 103.17E | 20.8 | 8.1 | 1039 | 153.14 |  |  |  |  |  |  |  |  |  |
| Hypotrachyna pseudosinuosa | 1 | MAF-Lich 15386 | Tumin area, East Sikkim, INDIA | 2000 | 27.20N, 88.15E | 16.7 | 5.0 | 3030 | 857.13 |  |  |  |  |  |  |  |  |  |
| Hypotrachyna revoluta | 1 | MAF-Lich 6047 | Puerto Urkiola, Vizcaya, SPAIN | 800 | 40.06N, 2.38W | 16.5 | 2.4 | 1549 | 277.56 |  |  |  |  |  |  |  |  |  |
| Hypotrachyna revoluta | 1 | MAF-Lich 10394 | Chu Xiong County, Yunnan, CHINA | 2200 | 24.59N, 101.26E | 22.2 | 10.0 | 821 | 97.32 |  |  |  |  |  |  |  |  |  |
| Hypotrachyna revoluta | 1 | MAF-Lich 10406 | Las Mercedes,Tenerife, CANARY ISLAND | 1200 | 28.32N, 16.12W | 19.1 | 13 | 518 | 102.45 |  |  |  |  |  |  |  |  |  |
| Hypotrachyna rockii | 1 | MAF-Lich 13965 | Parque Nacional de Huascaran, Quebrada Paron, PERU | 3860 | 9.50S, 77.50W | 13 | 9.5 | 724 | 181 |  |  |  |  |  |  |  |  |  |
| Hypotrachyna rockii | 1 | MAF-Lich 2919 | Sierra Portugues, Lara Prov., VENEZUELA | 1900 | 10.08N, 69.52W | 22.8 | 21.4 | 1796 | 294.29 |  |  |  |  |  |  |  |  |  |
| Hypotrachyna rockii | 1 | LWU 70264 | Kodaikanal, Palni hills, INDIA | 2250 | 11.24N, 76.43E | 28.5 | 21.7 | 1398 | 159.59 |  |  |  |  |  |  |  |  |  |
| Hypotrachyna scytophylla | 2 | MAF-Lich 10410 | Jian Chuan County,Yunnan, CHINA | 2500 | 26.21N, 99.50E | 19.2 | 7.6 | 963 | 140.45 |  |  |  |  |  |  |  |  |  |
| Hypotrachyna scytophylla | 2 | MAF-Lich 13973 | Kullu district, Himachal Pradesh, INDIA | 3000 | 32.01N, 77.02E | 19.4 | 5.3 | 1577 | 306.21 |  |  |  |  |  |  |  |  |  |
| Hypotrachyna scytophylla | 2 | LWU 67419 | Sandakhpoo, Darjeeling, district, W. Bengal, INDIA | 3500 | 26.59N, 88.17E | 16.7 | 5.0 | 3037 | 857.13 |  |  |  |  |  |  |  |  |  |
| Hypotrachyna sinuosa | 1 | MAF-Lich 10179 | Apple cross, Wester Ross, Scotland, GREAT BRITAIN | 63 | 57.26N, 5.49W | 12.5 | 3.6 | 761 | 342.8 |  |  |  |  |  |  |  |  |  |
| Hypotrachyna sinuosa | 1 | MAF-Lich 10396 | Jian Chuan County, Yunnan, CHINA | 3200 | 26.31N, 99.43E | 19.2 | 7.6 | 963 | 140.45 |  |  |  |  |  |  |  |  |  |
| Hypotrachyna sinuosa | 1 | MAF-Lich 15393 | Mt. Albert Edward sumit area, PAPUA NEW GUINEA | 3700 | 8.23S, 147.23E | 26.9 | 17.1 | 1011 | 185.85 |  |  |  |  |  |  |  |  |  |
| Hypotrachyna taylorensis | 1 | MAF-Lich 9912 | Tayvallich, Argyll and Bute, Scotland, GREAT BRITAIN | 13 | 56.01N, 5.37W | 16.4 | 7 | 869 | 247.3 |  |  |  |  |  |  |  |  |  |
| Hypotrachyna taylorensis | 1 | MAF-Lich 10405 | Las Mercedes, Tenerife, CANARY ISLAND | 900 | 28.30N, 16.11W | 19.1 | 13 | 518 | 102.45 |  |  |  |  |  |  |  |  |  |
| Hypotrachyna taylorensis | 1 | MAF-Lich 10407 | Gondomar, Pontavedra, SPAIN | 29 | 42.06N, 8.45W | 20.5 | 9.2 | 1595 | 250.79 |  |  |  |  |  |  |  |  |  |
| Karoowia saxeti | 3 | Aproot 53350 | Kenting National Park, Pingtung County, TAIWAN | 300 | 22.40N, 120.28E | 28.3 | 16.7 | 1039 | 142.57 |  |  |  |  |  |  |  |  |  |
| Karoowia saxeti | 3 | Degelius s.n. | Bimbe, Huila, ANGOLA | 1300 | 15.04S, 16.39E | 23.9 | 17.8 | 1027 | 106.98 |  |  |  |  |  |  |  |  |  |
| Karoowia saxeti | 3 | AA 3310 | Umtamvuna, Nature Reserve, Natal, SOUTH AFRICA | 899 | 28.56S, 30.48E | 20.4 | 11.4 | 897 | 139.01 |  |  |  |  |  |  |  |  |  |
| Melanohalea aff. elegantula | 5 | 16550 Esslinger | Wheeler County, Umatilla National forest, Oregón, USA | 1270 | 44.56N, 119.42W | 20.3 | -3.6 | 289 | 35.23 |  |  |  |  |  |  |  |  |  |
| Melanelia aff. elegantula | 5 | 16362 Esslinger | Idaho, Kooteniv County, Oregón, USA | 665 | 47.40N, 116.46W | 24.7 | -2.5 | 236 | 22.24 |  |  |  |  |  |  |  |  |  |
| Melanelia aff. elegantula | 5 | H 2208 | Wild basin, Boulder co., Colorado, USA | 1665 | 40.00N, 105.08W | 23.1 | -1.1 | 387 | 43.07 |  |  |  |  |  |  |  |  |  |
| Melanohalea aff. exasperata | 5 | MAF-Lich 10227 | Lago del Valle, Parque Natural de Somiedo, Asturias, SPAIN | 1500 | 43.04N, 6.11W | 18.8 | 1.7 | 936 | 136.05 |  |  |  |  |  |  |  |  |  |
| Melanelia aff. exasperata | 5 | MAF 10230 | Parque Natural Somiedo, Lago del Valle, Asturias, SPAIN | 1500 | 43.04N, 6.11W | 18.8 | 1.7 | 936 | 136.05 |  |  |  |  |  |  |  |  |  |
| Melanelia aff. exasperata | 5 | MAF 10225 | Las Médulas, Leon, SPAIN | 663 | 42.28N, 6.46W | 21 | 4.8 | 901 | 109.49 |  |  |  |  |  |  |  |  |  |
| Melanohalea elegantula | 5 | MAF-Lich 10218 | Mataelpino, Madrid, SPAIN | 1040 | 40.44N, 3.56W | 19.9 | 3.4 | 725 | 96.7 |  |  |  |  |  |  |  |  |  |
| Melanohalea elegantula | 5 | MAF-Lich 10226 | Cercedilla, Madrid, SPAIN | 1300 | 40.44N, 4.03W | 19.9 | 3.4 | 725 | 96.7 |  |  |  |  |  |  |  |  |  |
| Melanohalea elegantula | 5 | MAF-Lich 10218 | Corachar, Castellón, SPAIN | 1160 | 40.40N, 0.05E | 17.3 | 2 | 784 | 137.72 |  |  |  |  |  |  |  |  |  |
| Melanohalea exasperata | 5 | MAF-Lich 7636 | Sierra de Grazalema, Cádiz, SPAIN | 1100 | 36.45N, 5.22W | 25.2 | 7.5 | 1962 | 208.99 |  |  |  |  |  |  |  |  |  |
| Melanohalea exasperata | 5 | MAF-Lich 11386 | Torremocha, Guadalajara, SPAIN | 450 | 40.58N, 2.37W | 21.7 | 2.8 | 601 | 60.84 |  |  |  |  |  |  |  |  |  |
| Melanohalea exasperata | 5 | MAF-Lich 13059 | Herbes, Castellón, SPAIN | 850 | 40.37N, 0.04E | 20.3 | 3.6 | 599 | 86.6 |  |  |  |  |  |  |  |  |  |
| Melanohalea exasperatula | 5 | MAF-Lich 10213 | Cercedilla, Madrid, SPAIN | 1200 | 40.44N, 4.03W | 19.9 | 3.4 | 725 | 96.7 |  |  |  |  |  |  |  |  |  |
| Melanohalea exasperatula | 5 | MAF-Lich 13093 | Corachar, Castellón, SPAIN | 1160 | 40.40N, 0.05E | 17.3 | 2 | 784 | 137.72 |  |  |  |  |  |  |  |  |  |
| Melanohalea exasperatula | 5 | MAF-Lich 5157 | Niedere, Tauern, Steiermark, AUSTRIA | 1340 | 47.19N, 13.46E | 21.1 | -1 | 452 | 62.01 |  |  |  |  |  |  |  |  |  |
| Melanohalea olivacea | 5 | Vitikanen 16196 | Kn. Puolanca, NW end of Ristijärvi, FINLAND | 150 | 64.52N, 27.40E | 11.4 | -8.2 | 544 | 232.12 |  |  |  |  |  |  |  |  |  |
| Melanohalea olivacea | 5 | Trelease 1899 (US) | Glacier Bay, Eastern pacific Coast Distr., Alaska, USA | 2800 | 61.08N, 141.08W | 11.1 | -16.7 | 525 | -335.23 |  |  |  |  |  |  |  |  |  |
| Melanohalea olivacea | 5 | Lynge 1914 (US) | Nordbynesset, Troms, NORWAY | 164 | 69.17N, 18.59E | 12.8 | -16.4 | 335 | -212.22 |  |  |  |  |  |  |  |  |  |
| Melanohalea septentrionalis | 5 | Athi 60893 | Keski-Pohjanmaa, FINLAND | 103 | 63.40N, 23.56E | 17.2 | -10 | 640 | 208.44 |  |  |  |  |  |  |  |  |  |
| Melanohalea septentrionalis | 5 | MAF-Lich 5325 | Larimer County, Rocky Mont National Park, Colorado, USA | 900 | 40.25N, 105.31W | 23.1 | -1.1 | 387 | 43.07 |  |  |  |  |  |  |  |  |  |
| Melanohalea septentrionalis | 5 | Llano 462a (US) | Anaktuvuk Pass, Erctic Coast District, Alaska, USA | 1250 | 68.05N, 151.50W | 14.7 | -24.5 | 334 | -85.72 |  |  |  |  |  |  |  |  |  |
| Melanohalea subelegantula | 5 | Esslinger 16132 | Prairie city, Grant County, Oregón, USA | 1585 | 44.27N, 118.42W | 20.3 | -3.6 | 289 | 35.23 |  |  |  |  |  |  |  |  |  |
| Melanohalea subelegantula | 5 | Esslinger 14037 | Gifford Pinchot National forest, Wallowa County, Oregon, USA | 1700 | 45.47N, 121.37W | 18.9 | -0.3 | 230 | 27.61 |  |  |  |  |  |  |  |  |  |
| Melanohalea subelegantula | 5 | Esslinger 1715 | Southeast of Enterprise along the Lostine river, Wallowa County, Oregón, USA | 1700 | 45.27N, 117.28W | 24.7 | -2.5 | 236 | 20.09 |  |  |  |  |  |  |  |  |  |
| Melanohalea subolivacea | 5 | 16555 Esslinger | Wheeler County, Umatilla National forest, Oregón, USA | 1270 | 44.56N, 119.42E | 20.3 | -3.6 | 289 | 35.23 |  |  |  |  |  |  |  |  |  |
| Melanohalea subolivacea | 5 | 15576 Esslinger | Cochise County, Coronado National forest, Arizona, USA | 2550 | 31.55N, 109.16W | 24.2 | 2.5 | 310 | 30.66 |  |  |  |  |  |  |  |  |  |
| Melanohalea subolivacea | 5 | 16555 Esslinger | Apache County, Mountain Baldy, Wilderness, Arizona, USA | 2900 | 33.55N, 109.30W | 20 | -0.6 | 557 | 72.2 |  |  |  |  |  |  |  |  |  |
| Parmelaria subthomsonii | 4 | LWG 20-77151 (MAF-Lich 7654) | Tsomgo lake,Sikkim, INDIA | 3700 | 27.23N, 88.45E | 21 | 6 | 1301 | 210.95 |  |  |  |  |  |  |  |  |  |
| Parmelaria subthomsonii | 4 | LWG 3979 | Pithoragarh distt., Uttaranchal, INDIA | 1650 | 29.35N, 80.11E | 21 | 6 | 1301 | 210.95 |  |  |  |  |  |  |  |  |  |
| Parmelinella wallichiana | 2 | LWG 20-77171 (MAF-Lich 7653) | Gangtok, Sikkim, INDIA | 1750 | 27.20N, 88.36E | 16.7 | 5.0 | 3030 | 857.13 |  |  |  |  |  |  |  |  |  |
| Parmelinella wallichiana | 2 | MAF 10411 | Jian Chuan County, Yunnan, CHINA | 2500 | 26.22N, 99.49E | 19.2 | 7.6 | 963 | 140.45 |  |  |  |  |  |  |  |  |  |
| Parmelinella wallichiana | 2 | LWU 76212 | Ratangla, Bherizone, NEPAL | 2400 | 28.46N, 81.51E | 25 | 12.5 | 3388 | 397.63 |  |  |  |  |  |  |  |  |  |
| Parmelinopsis cryptochlora | 1 | MAF-Lich 10398 | Chu Xiong County, Yunnan, CHINA | 2200 | 24.59N, 101.26E | 22.2 | 10.0 | 821 | 97.32 |  |  |  |  |  |  |  |  |  |
| Parmelinopsis cryptochlora | 1 | 40203 Hale | Nilgiri hills, Tamil Nadu, INDIA | 2100 | 11.24N, 76.41E | 28.5 | 21.7 | 1398 | 159.59 |  |  |  |  |  |  |  |  |  |
| Parmelinopsis cryptochlora | 1 | BM 912 | Laudat, DOMINICA | 548 | 15.19N, 61.19W | 27 | 23.6 | 1769 | 290.02 |  |  |  |  |  |  |  |  |  |
| Parmelinopsis horrescens | 1 | MAF-Lich 9913 | Viduido, Bentin, La Coruña, SPAIN | 350 | 42.50N, 8.36W | 18.4 | 7.6 | 1545 | 278.68 |  |  |  |  |  |  |  |  |  |
| Parmelinopsis horrescens | 1 | MAF-Lich 10399 | Las Mercedes, Tenerife, CANARY ISLAND | 900 | 28.30N, 16.11W | 19.1 | 13 | 518 | 102.45 |  |  |  |  |  |  |  |  |  |
| Parmelinopsis horrescens | 1 | MAF-Lich 10400 | Gondomar, Pontevedra, SPAIN | 20 | 42.06N, 8.45W | 20.5 | 9.2 | 1595 | 250.79 |  |  |  |  |  |  |  |  |  |
| Parmelinopsis minarum | 1 | MAF-Lich 7639 | Parque Natural de los Alcornocales, Facinas, Cádiz, SPAIN | 220 | 36.08N, 5.41W | 21.8 | 11.6 | 1065 | 130.9 |  |  |  |  |  |  |  |  |  |
| Parmelinopsis minarum | 1 | MAF-Lich 10401 | Las Mercedes, Tenerife, CANARY ISLAND | 900 | 28.30N, 16.11W | 19.1 | 13 | 518 | 102.45 |  |  |  |  |  |  |  |  |  |
| Parmelinopsis minarum | 1 | 16753 Elix | Tully Falls Rd, Queensland, AUSTRALIA | 864 | 17.43S, 145.32E | 27.8 | 22.5 | 1773 | 273.73 |  |  |  |  |  |  |  |  |  |
| Parmelinopsis neodamaziana | 1 | MAF-Lich 10182 | Morton National Park, Pidgeon House Mountain, AUSTRALIA | 750 | 34.59S 150.25E | 19.2 | 6.9 | 1511 | 228.18 |  |  |  |  |  |  |  |  |  |
| Parmelinopsis neodamaziana | 1 | 37258 Elix | New South Wales, Wash pool national park, Gibraltar range, AUSTRALIA | 895 | 29.28S, 152.21E | 22.2 | 7.8 | 761 | 84.5 |  |  |  |  |  |  |  |  |  |
| Parmelinopsis neodamaziana | 1 | 2343 Elix | New South Wales, 15 km W of Dorrigo, AUSTRALIA | 751 | 30.20S, 152.40E | 22.8 | 12.5 | 1612 | 241.29 |  |  |  |  |  |  |  |  |  |
| Parmelinopsis subfatiscens | 1 | MAF-Lich 6878 | Morton National Park, AUSTRALIA | 750 | 34.59S 150.25E | 19.2 | 6.9 | 1511 | 228.18 |  |  |  |  |  |  |  |  |  |
| Parmelinopsis subfatiscens | 1 | MAF-Lich 15412 | Whangarei, North-land region, NEW ZEALAND | 100 | 35.43S, 174.17E | 18.3 | 8.9 | 2017 | 382.22 |  |  |  |  |  |  |  |  |  |
| Parmelinopsis subfatiscens | 1 | ANUC 4038 | Australian capital territory 11 Km SW of Tharwa, AUSTRALIA | 1135 | 35.38S, 148.58E | 20.8 | 12.1 | 1258 | 252.59 |  |  |  |  |  |  |  |  |  |
| Parmotrema cetratum | 4 | Osorio 9424 | Sierra de la Coronilla, cerca del cerro Catedral, Maldonado, URUGUAY | 250 | 34.21S, 54.38W | 23.3 | 11.7 | 1125 | 132.6 |  |  |  |  |  |  |  |  |  |
| Parmotrema cetratum | 4 | Osorio 9425 | Sierra de la Coronilla, cerca del cerro Catedral, Maldonado, URUGUAY | 250 | 34.21S, 54.38W | 23.3 | 11.7 | 1125 | 132.6 |  |  |  |  |  |  |  |  |  |
| Parmotrema cetratum | 4 | H-ACH sn | Pennsylvania, USA | 441 | 41N, 77.30W | 18.1 | -6.7 | 1009 | 213.62 |  |  |  |  |  |  |  |  |  |
| Parmotrema crinitum | 4 | MAF-Lich 6061 | Castello do Mouros, Sintra, PORTUGAL | 400 | 38.47N, 9.23W | 18.2 | 11.7 | 803 | 188.49 |  |  |  |  |  |  |  |  |  |
| Parmotrema crinitum | 4 | MAF-Lich 9891 | La Gomera, Tenerife, CANARY ISLAND | 1070 | 28.06N, 17.12W | 22.6 | 16.8 | 438 | 94.13 |  |  |  |  |  |  |  |  |  |
| Parmotrema crinitum | 4 | MAF-Lich 7605 | 13 km SSE batemats bay, Melville Point, AUSTRALIA | 1 | 35.72S, 150.18E | 19.7 | 13.6 | 1038 | 214.62 |  |  |  |  |  |  |  |  |  |
| Parmotrema fistulatum | 4 | Osorio 9423 (MAF-Lich 7655) | Pozo del Buey, cerro Catedral, Maldonado, URUGUAY | 100 | 34.25S, 54.33W | 23.3 | 11.7 | 1125 | 132.6 |  |  |  |  |  |  |  |  |  |
| Parmotrema fistulatum | 4 | Osorio 5671 | Laguna negra, Rocha, URUGAY | 143 | 34.00S, 53.58W | 22.8 | 11.1 | 1032 | 130.53 |  |  |  |  |  |  |  |  |  |
| Parmotrema fistulatum | 4 | US 20954 | Tandil, Buenos Aires, ARGENTINA | 176 | 37.14S, 59.14W | 21.4 | 7.5 | 791 | 88.36 |  |  |  |  |  |  |  |  |  |
| Parmotrema haitiense | 4 | MAF-Lich 7657 | Australian Capital Territory, Paddy River near Murrays Corner, AUSTRALIA | 550 | 35.22S, 148.58E | 19 | 5.1 | 976 | 118.74 |  |  |  |  |  |  |  |  |  |
| Parmotrema haitiense | 4 | CBG 7784 | New South Wales, Goulbourn, Wombeyancaves, AUSTRALIA | 818 | 35.19S, 150.01E | 20.8 | 12.1 | 1258 | 252.59 |  |  |  |  |  |  |  |  |  |
| Parmotrema haitiense | 4 | G 71 | Caracas, VENEZUELA | 897 | 10.29N, 66.53W | 28.1 | 25.8 | 882 | 172.14 |  |  |  |  |  |  |  |  |  |
| Parmotrema hypoleucinum | 4 | MAF-Lich 7637 | Parque Natural de los Alcornocales, Cádiz, SPAIN | 220 | 36.08N, 5.41W | 21.8 | 11.6 | 1065 | 130.9 |  |  |  |  |  |  |  |  |  |
| Parmotrema hypoleucinum | 4 | MAF-Lich 13143 | Villabona a Rosell, Castellón, SPAIN | 500 | 40.37N, 0.12E | 23.1 | 8.4 | 611 | 71.43 |  |  |  |  |  |  |  |  |  |
| Parmotrema hypoleucinum | 4 | 3p | Maamora, Rabat, MOROCCO | 68 | 34.01N, 6.42W | 23.1 | 12.5 | 506 | 68.31 |  |  |  |  |  |  |  |  |  |
| Parmotrema perforatum | 4 | Cole 7983 | Greene County, North Carolina, USA | 98 | 35.33N, 77.48W | 25.2 | 4.3 | 1072 | 112.47 |  |  |  |  |  |  |  |  |  |
| Parmotrema perforatum | 4 | MAF-Lich 1165 | Hardin County, 2km SE of obsaratoga, Texas, USA | 26 | 30.17N, 94.31W | 28.5 | 12.7 | 1063 | 124.11 |  |  |  |  |  |  |  |  |  |
| Parmotrema perforatum | 4 | MAF-Lich 1153 | Brazos County, 9km Nw of Bryan, Texas, USA | 100 | 30.49N, 96.31W | 29.5 | 11.1 | 899 | 70.66 |  |  |  |  |  |  |  |  |  |
| Parmotrema perlatum | 4 | MAF-Lich 6965 | Castello do Mouros, Sintra, PORTUGAL | 400 | 38.47N, 9.23W | 18.2 | 11.7 | 803 | 188.49 |  |  |  |  |  |  |  |  |  |
| Parmotrema perlatum | 4 | MAF-Lich 15331 | Braganza, Tras-os-Montes, PORTUGAL | 680 | 41.49N, 6.46W | 18.9 | 3.4 | 1195 | 204.4 |  |  |  |  |  |  |  |  |  |
| Parmotrema perlatum | 4 | MAF-Lich 7498 | Australian Capital territory, Molonglo Gorge Reserve, AUSTRALIA | 550 | 35.20S, 149.15E | 20.6 | 6.3 | 610 | 72.44 |  |  |  |  |  |  |  |  |  |
| Parmotrema pilosum | 4 | MAF-Lich 7656 | Sierra de la Coronilla, Maldonado, URUGUAY | 250 | 34.21S, 54.38W | 23.3 | 11.7 | 1125 | 132.6 |  |  |  |  |  |  |  |  |  |
| Parmotrema pilosum | 4 | Elix 3553 (ANUC) | W of Connabarabran on road to Siding Springs, AUSTRALIA | 600 | 31.16S, 149.10E | 25 | 9.2 | 672 | 62.89 |  |  |  |  |  |  |  |  |  |
| Parmotrema pilosum | 4 | TRH sn | Natal, Cape prov., SOUTH AFRICA | 899 | 28.56S, 30.48E | 20.4 | 11.4 | 897 | 139.01 |  |  |  |  |  |  |  |  |  |
| Parmotrema pseudoreticulatum | 4 | MAF-Lich 7650 | Parque natural de los Alcornocales, entre Facinas y Barrios, Cádiz, SPAIN | 350 | 36.08N, 5.41W | 21.8 | 11.6 | 1065 | 130.9 |  |  |  |  |  |  |  |  |  |
| Parmotrema pseudoreticulatum | 4 | MAF-Lich 10278 | Santo antao do Tojal, Estremadura, PORTUGAL | 50 | 38.51N, 9.08W | 22.8 | 14.5 | 1024 | 192.29 |  |  |  |  |  |  |  |  |  |
| Parmotrema pseudoreticulatum | 4 | MAF-Lich 10288 | Addo Elephant National Park, Eastern Cape, SOUTH AFRICA | 31 | 33.33S, 25.40E | 22.5 | 13 | 610 | 78.72 |  |  |  |  |  |  |  |  |  |
| Parmotrema reticulatum | 4 | MAF-Lich 6067 | Castello do Mouros, Sintra, PORTUGAL | 400 | 38.47N, 9.23W | 18.2 | 11.7 | 803 | 188.49 |  |  |  |  |  |  |  |  |  |
| Parmotrema reticulatum | 4 | MAF-Lich 10267 | Las Mercedes, Tenerife, CANARY ISLAND | 900 | 28.30N, 16.11W | 19.1 | 13 | 518 | 102.45 |  |  |  |  |  |  |  |  |  |
| Parmotrema reticulatum | 4 | CANB (MAF 10286) | New South Wales, Brigdon Farm, AUSTRALIA | 30 | 36.33S, 150.03E | 22.3 | 5.8 | 584 | 70.63 |  |  |  |  |  |  |  |  |  |
| Parmotrema robustum | 4 | MAF-Lich 10166 | Nazaré, PORTUGAL | 70 | 39.35N, 9.03W | 17.8 | 10.7 | 1241 | 245.41 |  |  |  |  |  |  |  |  |  |
| Parmotrema robustum | 4 | MAF-Lich 7066 | Hervás, Caceres, SPAIN | 688 | 40.16N, 5.51W | 23.8 | 6.2 | 1138 | 105.28 |  |  |  |  |  |  |  |  |  |
| Parmotrema robustum | 4 | MAF-Lich 10166 | Castello do Mouro, Sintra, PORTUGAL | 427 | 38.47N, 9.23W | 18.2 | 11.7 | 803 | 188.49 |  |  |  |  |  |  |  |  |  |
| Parmotrema subcaperatum | 4 | HO 324283 | Grasstree hill, Tasmania, AUSTRALIA | 400 | 42.47S, 147.21E | 16.3 | 7.8 | 654 | 144.48 |  |  |  |  |  |  |  |  |  |
| Parmotrema subcaperatum | 4 | PERTH sn | Along south face at base of Devils slide, Porongurup National Park, AUSTRALIA | 583 | 34.40S, 117.50E | 23.9 | 13.1 | 889 | 113.23 |  |  |  |  |  |  |  |  |  |
| Parmotrema subcaperatum | 4 | MEL 5466 | Mt. Glorious, Queensland, AUSTRALIA | 672 | 27.19S, 152.45E | 22 | 10.6 | 927 | 123.94 |  |  |  |  |  |  |  |  |  |
| Parmotrema subtinctorium | 4 | GPGC 02-000696 | Goriganga Catchment, Uttaranchal, INDIA | 1400 | 29.60N, 80.20E | 21 | 6 | 1301 | 210.95 |  |  |  |  |  |  |  |  |  |
| Parmotrema subtinctorium | 4 | LWU 669 | Kodaikanal, Palni hills, INDIA | 2100 | 11.24N, 76.43E | 28.5 | 21.7 | 1398 | 159.59 |  |  |  |  |  |  |  |  |  |
| Parmotrema subtinctorium | 4 | ANUC 2633 | Atherton, Queensland, AUSTRALIA | 758 | 17.15S, 145.28E | 27 | 15 | 950 | 81.97 |  |  |  |  |  |  |  |  |  |
| Parmotrema tinctorum | 4 | MAF-Lich 10163 | 13 km SSE batemats bay, Melville Point, AUSTRALIA | 1 | 35.72S, 150.18E | 19.7 | 13.6 | 1038 | 214.62 |  |  |  |  |  |  |  |  |  |
| Parmotrema tinctorum | 4 | MAF-Lich 10503 | New South Wales, Brigadoon form, AUSTRALIA | 30 | 36.33S, 150.03E | 22.3 | 5.8 | 584 | 70.63 |  |  |  |  |  |  |  |  |  |
| Parmotrema tinctorum | 4 | AWAS 2707 | Pithoragarh district, Uttaranchal, INDIA | 1350 | 29.35N, 80.11E | 21 | 6 | 1301 | 210.95 |  |  |  |  |  |  |  |  |  |
| Xanthoparmelia conspersa | 3 | MAF-Lich 6793 | Puebla de Sanabria, Zamora, SPAIN | 960 | 42.03N, 6.38W | 18.3 | 3 | 946 | 131.09 |  |  |  |  |  |  |  |  |  |
| Xanthoparmelia conspersa | 3 | MAF-Lich 14092 | Mataelpino, Madrid, SPAIN | 1080 | 40.44N, 3.56W | 19.9 | 3.4 | 725 | 96.7 |  |  |  |  |  |  |  |  |  |
| Xanthoparmelia conspersa | 3 | MAF-Lich 10539 | Anguiano, La Rioja, SPAIN | 654 | 42.22N, 2.30W | 18.2 | 2.9 | 861 | 142.28 |  |  |  |  |  |  |  |  |  |
| Xanthoparmelia crespoae | 3 | MAF-Lich 7524 | New South Wales, Braidwood, AUSTRALIA | 600 | 35.26S, 149.48E | 19 | 5.4 | 760 | 84.35 |  |  |  |  |  |  |  |  |  |
| Xanthoparmelia crespoae | 3 | MAF-Lich 7440 | New South Wales, Braidwood, AUSTRALIA | 600 | 35.26S, 149.48E | 19 | 5.4 | 760 | 84.35 |  |  |  |  |  |  |  |  |  |
| Xanthoparmelia crespoae | 3 | MAF-Lich 7456 | New South Wales, Braidwood, AUSTRALIA | 600 | 35.26S, 149.48E | 19 | 5.4 | 760 | 84.35 |  |  |  |  |  |  |  |  |  |
| Xanthoparmelia delisei | 3 | MAF-Lich 7659 | Arroyo del Zape, Zamora, SPAIN | 690 | 41.28N, 5.47W | 23.6 | 4.2 | 443 | 39.48 |  |  |  |  |  |  |  |  |  |
| Xanthoparmelia delisei | 3 | MAF-Lich 9886 | Puerto de la Morcuera, Madrid, SPAIN | 1700 | 40.49N, 3.49W | 22.7 | 3.9 | 692 | 79.52 |  |  |  |  |  |  |  |  |  |
| Xanthoparmelia delisei | 3 | MAF-Lich 7659 | New South Wales, Braidwood, AUSTRALIA | 600 | 35.21S, 149.44E | 19 | 5.4 | 760 | 84.35 |  |  |  |  |  |  |  |  |  |
| Xanthoparmelia digitiformis | 3 | MAF-Lich 7525 | Australian Capital Territory, Brindabella Range, AUSTRALIA | 1644 | 35.20S, 148.43E | 17.3 | 2.9 | 777 | 118.52 |  |  |  |  |  |  |  |  |  |
| Xanthoparmelia digitiformis | 3 | MAF-Lich 7546 | Australian Capital Territory, Black Mountain Reserve, Canberra, AUSTRALIA | 750 | 35.16S, 149.05E | 20.3 | 5.8 | 584 | 75.63 |  |  |  |  |  |  |  |  |  |
| Xanthoparmelia digitiformis | 3 | MAF-Lich 7560 | Australian Capital Territory, Black Mountain Reserve, Canberra, AUSTRALIA | 750 | 35.16S, 149.05E | 20.3 | 5.8 | 584 | 75.63 |  |  |  |  |  |  |  |  |  |
| Xanthoparmelia glabrans | 3 | MAF-Lich 7665 | Australian capital territory, Brindabella Range, AUSTRALIA | 1644 | 35.20S, 148.43E | 17.3 | 2.9 | 777 | 118.52 |  |  |  |  |  |  |  |  |  |
| Xanthoparmelia glabrans | 3 | BRI 2877 | Girraween National Park, Queensland, AUSTRALIA | 1000 | 28.52S, 151.58E | 22.2 | 7.8 | 761 | 84.5 |  |  |  |  |  |  |  |  |  |
| Xanthoparmelia glabrans | 3 | MAF-Lich 9912 | Embid de la Rivera, Zaragoza, SPAIN | 480 | 41.25N, 1.35W | 23.3 | 5 | 434 | 44.6 |  |  |  |  |  |  |  |  |  |
| Xanthoparmelia huenana | 3 | GZU 46511 | Trail E of Swakopmund, NAMIBIA | 1 | 22.40S, 14.32E | 19.2 | 13.4 | 16 | 4.75 |  |  |  |  |  |  |  |  |  |
| Xanthoparmelia huenana | 3 | Jensen 6 (US) | Near Walvis bay, NAMIBIA | 20 | 22.59S, 14.32E | 18 | 13.5 | 18 | 4.69 |  |  |  |  |  |  |  |  |  |
| Xanthoparmelia huenana | 3 | Moisel 5170 (M; US) | 10 km E of Swakopmund, NAMIBIA | 20 | 22.39S, 14.32E | 19.2 | 13.4 | 16 | 4.75 |  |  |  |  |  |  |  |  |  |
| Xanthoparmelia isidiovagans | 3 | MAF-Lich 9956 | Torremocha, Guadalajara, SPAIN | 450 | 40.58N, 2.37W | 21.7 | 2.8 | 601 | 60.84 |  |  |  |  |  |  |  |  |  |
| Xanthoparmelia isidiovagans | 3 | 19612a | Tandir village, Eskisehir prov., Turkey | 1350 | 39.55N, 30.40E | 23.3 | 1.7 | 671 | 71.84 |  |  |  |  |  |  |  |  |  |
| Xanthoparmelia lithophila | 3 | MAF-Lich 6900 | New South Wales, Braidwood, AUSTRALIA | 600 | 35.26S, 149.48E | 19 | 5.4 | 760 | 84.35 |  |  |  |  |  |  |  |  |  |
| Xanthoparmelia lithophila | 3 | MAF-Lich 9661 | Morton National Park, AUSTRALIA | 750 | 35.21S 150.15E | 20.3 | 5.8 | 584 | 75.63 |  |  |  |  |  |  |  |  |  |
| Xanthoparmelia lithophila | 3 | MAF-Lich 10505 | New South Wales, Brigadoon farm, AUSTRALIA | 30 | 36.34S, 150.03E | 22.3 | 5.8 | 584 | 70.63 |  |  |  |  |  |  |  |  |  |
| Xanthoparmelia loxodes | 3 | MAF-Lich 7072 | Béjar, Salamanca, SPAIN | 1200 | 40.22N, 5.46W | 19.6 | 3 | 884 | 116.27 |  |  |  |  |  |  |  |  |  |
| Xanthoparmelia loxodes | 3 | MAF-Lich 6206 | Laguna de los Peces, Zamora, SPAIN | 1650 | 42.09N, 6,43W | 18.3 | 3 | 946 | 131.09 |  |  |  |  |  |  |  |  |  |
| Xanthoparmelia loxodes | 3 | MAF 6206 | Mataelpino, Madrid, SPAIN | 1080 | 40.44N, 3.56W | 19.9 | 3.4 | 725 | 96.7 |  |  |  |  |  |  |  |  |  |
| Xanthoparmelia mougeotii | 3 | MAF-Lich 9916 | Montenegro, La Rioja, SPAIN | 1250 | 42.05N, 2.45W | 17.5 | 1 | 548 | 88.96 |  |  |  |  |  |  |  |  |  |
| Xanthoparmelia mougeotii | 3 | G s.n. | Near Concepcion, ARGENTINA | 663 | 28.40S, 65.24W | 26.8 | 11.5 | 338 | 27.39 |  |  |  |  |  |  |  |  |  |
| Xanthoparmelia mougeotii | 3 | MAF-Lich 7472 | Australian Capital territory, Molonglo Gorge Reserve, AUSTRALIA | 550 | 35.20S, 149.15E | 20.6 | 6.3 | 610 | 72.44 |  |  |  |  |  |  |  |  |  |
| Xanthoparmelia murina | 3 | MAF-Lich 9915 | Australian Capital Territory, Molonglo Gorge Reserve, AUSTRALIA | 550 | 35.22S, 149.21E | 20.6 | 6.3 | 610 | 72.44 |  |  |  |  |  |  |  |  |  |
| Xanthoparmelia murina | 3 | MAF-Lich 7580 | Currowan state forest, 15km from Batemans bay, AUSTRALIA | 60 | 35.28S, 150.05E | 20.3 | 5.8 | 584 | 75.63 |  |  |  |  |  |  |  |  |  |
| Xanthoparmelia murina | 3 | MAF-Lich 7586 | Currowan state forest, 15km from Batemans bay, AUSTRALIA | 60 | 35.28S, 150.05E | 20.3 | 5.8 | 584 | 75.63 |  |  |  |  |  |  |  |  |  |
| Xanthoparmelia norcapnhodes | 3 | MAF-Lich 7532 | Australian Capital Territory, Molonglo Gorge Reserve, AUSTRALIA | 550 | 35.22S, 149.21E | 20.6 | 6.3 | 610 | 72.44 |  |  |  |  |  |  |  |  |  |
| Xanthoparmelia norcapnhodes | 3 | MAF-Lich 7401 | Australian Capital territory, 4km S of Tharwa, AUSTRALIA | 650 | 35.34S, 149.05E | 20.6 | 6.3 | 610 | 72.44 |  |  |  |  |  |  |  |  |  |
| Xanthoparmelia norcapnhodes | 3 | Elix 1030 (ANUC) | W slopes of Black Mnt, Canberra, AUSTRALIA | 500 | 30.32S, 152.18E | 25 | 9.2 | 672 | 62.89 |  |  |  |  |  |  |  |  |  |
| Xanthoparmelia notata | 3 | Elix 42648 (MAF-Lich 7521) | Australian Capital Territory, Aranda Bushland, AUSTRALIA | 680 | 35.15S, 149.04E | 20.3 | 5.8 | 584 | 75.63 |  |  |  |  |  |  |  |  |  |
| Xanthoparmelia notata | 3 | MAF-Lich 7429 | New South Wales, Braidwood, AUSTRALIA | 600 | 35.21S, 149.44E | 19 | 5.4 | 760 | 84.35 |  |  |  |  |  |  |  |  |  |
| Xanthoparmelia notata | 3 | MAF-Lich 7488 | Australian Capital Territory, Molonglo Gorge Reserve, AUSTRALIA | 550 | 35.20S, 149.15E | 20.6 | 6.3 | 610 | 72.44 |  |  |  |  |  |  |  |  |  |
| Xanthoparmelia pokornyii | 3 | MAF-Lich 9908 | Embid de la Ribera, Zaragoza, SPAIN | 480 | 41.25N, 1.35W | 23.3 | 5 | 434 | 44.6 |  |  |  |  |  |  |  |  |  |
| Xanthoparmelia pokornyi | 3 | MAF-Lich 6052 | Zaorejas, Guadalajara, SPAIN | 1250 | 40.44N, 2.11W | 22 | 3.5 | 668 | 73.76 |  |  |  |  |  |  |  |  |  |
| Xanthoparmelia pokornyi | 3 | MAF-Lich 11395 | Zaorejas, Guadalajara, SPAIN | 1250 | 40.44N, 2.11W | 22 | 3.5 | 668 | 73.76 |  |  |  |  |  |  |  |  |  |
| Xanthoparmelia protomatrae | 3 | MAF-Lich 6216 | Puebla de Sanabria, Zamora, SPAIN | 960 | 42.03N, 6.38W | 18.3 | 3 | 946 | 131.09 |  |  |  |  |  |  |  |  |  |
| Xanthoparmelia protomatrae | 3 | MAF-Lich 9847 | Port de la selva, Gerona, SPAIN | 30 | 42.19N, 3.08E | 23.3 | 7.2 | 582 | 72.08 |  |  |  |  |  |  |  |  |  |
| Xanthoparmelia protomatrae | 3 | MAF-Lich 3777 | Between Berrueco and Berzosa del Lozoya, Madrid, SPAIN | 900 | 40.56N, 3,33W | 21.1 | 3.2 | 650 | 75.84 |  |  |  |  |  |  |  |  |  |
| Xanthoparmelia pulla | 3 | MAF-Lich 6794 | Mataelpino, Madrid, SPAIN | 1080 | 40.44N, 3.56W | 19.9 | 3.4 | 725 | 96.7 |  |  |  |  |  |  |  |  |  |
| Xanthoparmelia pulla | 3 | MAF-Lich 12517 | Ezcaray, La Rioja, SPAIN | 1780 | 42.03N, 3.03W | 17.5 | 1 | 548 | 88.96 |  |  |  |  |  |  |  |  |  |
| Xanthoparmelia pulla | 3 | MAF-Lich 7058 | La Alberca, Salamanca, SPAIN | 1200 | 40.29N, 6.07W | 21.7 | 3.8 | 551 | 61.63 |  |  |  |  |  |  |  |  |  |
| Xanthoparmelia pulloides | 3 | MAF-Lich 6784 | Carretera Zamora-Almaráz, Zamora, SPAIN | 700 | 41.28N, 5.47W | 21.9 | 4.3 | 415 | 46.43 |  |  |  |  |  |  |  |  |  |
| Xanthoparmelia pulloides | 3 | Galun 128 | Upper Jordan valley, Korazim, Oregón, USA | 1300 | 43.01N, 116.54W | 20.3 | -3.6 | 289 | 35.23 |  |  |  |  |  |  |  |  |  |
| Xanthoparmelia pulloides | 3 | Culberson & Culberson 16099A | Between Tejina and Valle-Guerra, ca. 2 km from the sea, Tenerife, CANARY ISLAND | 200 | 28.32N, 16.21W | 22 | 15.4 | 450 | 84.01 |  |  |  |  |  |  |  |  |  |
| Xanthoparmelia scotophylla | 3 | Elix 30650 | Mount Remarkable National Park, Mambray Creek Section, AUSTRALIA | 50 | 32.50S, 137.58E | 25.3 | 11.1 | 229 | 20.62 |  |  |  |  |  |  |  |  |  |
| Xanthoparmelia scotophylla | 3 | Elix 17795 | Warren Gorge, 18.5 km N of Quorn, AUSTRALIA | 100 | 32.01S, 138.01E | 27.2 | 10.3 | 247 | 20.38 |  |  |  |  |  |  |  |  |  |
| Xanthoparmelia scotophylla | 3 | Elix 3907 (MEL) | Trail to Pigeon House Mtn, 15 km W of Ulladulla, AUSTRALIA | 90 | 35.21S, 150.23E | 22.9 | 8.6 | 433 | 49.96 |  |  |  |  |  |  |  |  |  |
| Xanthoparmelia semiviridis | 3 | MAF-Lich 6876 | New South Wales, Bredbo, AUSTRALIA | 760 | 35.57S, 149.08E | 16.5 | 8.3 | 648 | 102.2 |  |  |  |  |  |  |  |  |  |
| Xanthoparmelia semiviridis | 3 | Elix 10883 (CANB) | New South Wales, 2 Km N of Bredbo, AUSTRALIA | 720 | 35.57S, 149.08E | 20.3 | 5.8 | 584 | 75.63 |  |  |  |  |  |  |  |  |  |
| Xanthoparmelia semiviridis | 3 | Elix 9364 (ANUC) | 8 km S of Black Hill Township, AUSTRALIA | 75 | 34.41S, 139.27E | 21.7 | 8.6 | 410 | 46.08 |  |  |  |  |  |  |  |  |  |
| Xanthoparmelia stenophylla | 3 | MAF-Lich 9917 | Torremocha, Guadalajara, SPAIN | 450 | 40.58N, 2.37W | 21.7 | 2.8 | 601 | 60.84 |  |  |  |  |  |  |  |  |  |
| Xanthoparmelia stenophylla | 3 | MAF-Lich 9846 | Mataelpino, Madrid, SPAIN | 1080 | 40.44N, 3.56W | 19.9 | 3.4 | 725 | 96.7 |  |  |  |  |  |  |  |  |  |
| Xanthoparmelia stenophylla | 3 | MAF-Lich 10549 | Between Anguiano and Mansilla, La Rioja, SPAIN | 910 | 42.10N, 2.52W | 18.2 | 2.9 | 861 | 142.28 |  |  |  |  |  |  |  |  |  |
| Xanthoparmelia subincerta | 3 | MAF-Lich 7494 | Australian capital Territory, Molonglo Gorge Reserve, AUSTRALIA | 550 | 35.22S, 149.21E | 20.6 | 6.3 | 610 | 72.44 |  |  |  |  |  |  |  |  |  |
| Xanthoparmelia subincerta | 3 | Elix 21806 (ANUC) | Kowen forest, 16 km E of Canberra, AUSTRALIA | 640 | 35.19S, 149.14E | 20.3 | 5.8 | 584 | 75.63 |  |  |  |  |  |  |  |  |  |
| Xanthoparmelia subincerta | 3 | COLO L49062 | E of Cooma on road to Numeralla, AUSTRALIA | 880 | 36.13S, 149.07E | 19 | 5.4 | 760 | 84.35 |  |  |  |  |  |  |  |  |  |
| Xanthoparmelia subspodochroa | 3 | MAF-Lich 7463 | New South Wales, Braidwood, AUSTRALIA | 600 | 35.26S, 149.48E | 19 | 5.4 | 760 | 84.35 |  |  |  |  |  |  |  |  |  |
| Xanthoparmelia subspodochroa | 3 | MAF-Lich 7459 | New South Wales, Braidwood, AUSTRALIA | 600 | 35.26S, 149.48E | 19 | 5.4 | 760 | 84.35 |  |  |  |  |  |  |  |  |  |
| Xanthoparmelia subspodochroa | 3 | Verdon 1699 (CBG) | Naas Ck, 57 km of Canberra, AUSTRALIA | 660 | 35.07S, 149.17E | 20.3 | 5.8 | 584 | 75.63 |  |  |  |  |  |  |  |  |  |
| Xanthoparmelia subprolixa | 3 | MAF-Lich 7667 | Australian Capital territory, Brindabella Range, AUSTRALIA | 1644 | 35.20S, 148.43E | 17.3 | 2.9 | 777 | 118.52 |  |  |  |  |  |  |  |  |  |
| Xanthoparmelia subprolixa | 3 | MAF-Lich 7484 | Australian Capital Territory, Molonglo Gorge reserve, AUSTRALIA | 550 | 35.22S, 149.21E | 20.6 | 6.3 | 610 | 72.44 |  |  |  |  |  |  |  |  |  |
| Xanthoparmelia subprolixa | 3 | MAF-Lich 7424 | New South Wales, Braidwood, AUSTRALIA | 600 | 35.21S, 149.44E | 20.6 | 6.3 | 610 | 72.44 |  |  |  |  |  |  |  |  |  |
| Xanthoparmelia tegeta | 3 | MAF-Lich 7523 | Australian Capital Territory, Brindabella Range, AUSTRALIA | 1644 | 35.20S, 148.43E | 17.3 | 2.9 | 777 | 118.52 |  |  |  |  |  |  |  |  |  |
| Xanthoparmelia tegeta | 3 | Elix 1718 | Diggers Creek, Kosciusko National Park, AUSTRALIA | 1100 | 36.03S, 148.30E | 19.7 | 9.5 | 653 | 104.6 |  |  |  |  |  |  |  |  |  |
| Xanthoparmelia tegeta | 3 | Streimann 9727 (CBG) | Bimberi Peak, Bimberi, AUSTRALIA | 1330 | 35.38S, 148.46E | 19 | 5.4 | 760 | 84.35 |  |  |  |  |  |  |  |  |  |
| Xanthoparmelia tinctina | 3 | BCN-13862 | Alt Empordá, Tarragona, SPAIN | 20 | 41.07N, 1.20W | 24.3 | 8.2 | 556 | 74.99 |  |  |  |  |  |  |  |  |  |
| Xanthoparmelia tinctina | 3 | MAF-Lich 6070 | Montejo de la Sierra, Madrid, SPAIN | 1100 | 41.03N, 3.31W | 19.9 | 3.4 | 725 | 96.7 |  |  |  |  |  |  |  |  |  |
| Xanthoparmelia tinctina | 3 | MAF-Lich 9883 | Mataelpino, Madrid, SPAIN | 1080 | 40.44N, 3.56W | 19.9 | 3.4 | 725 | 96.7 |  |  |  |  |  |  |  |  |  |
| Xanthoparmelia transvaalensis | 3 | MAF-Lich 9841 | Embid de la Ribera, Zaragoza, SPAIN | 480 | 41.25N, 1.35W | 23.3 | 5 | 434 | 44.6 |  |  |  |  |  |  |  |  |  |
| Xanthoparmelia transvaalensis | 3 | Hale 72038 | Long Tom Pass, Farm De Kuiten, Lydenburg, Transvaal, SOUTH AFRICA | 1740 | 25.10S, 30.37E | 20.4 | 9.9 | 730 | 106.4 |  |  |  |  |  |  |  |  |  |
| Xanthoparmelia verrucigera | 3 | MAF-Lich 9920 | Vilajuiga, Gerona, SPAIN | 25 | 42.19N, 3.05W | 23.3 | 7.2 | 582 | 72.08 |  |  |  |  |  |  |  |  |  |
| Xanthoparmelia verrucigera | 3 | MAF-Lich 10180 | Embid de la Ribera, Zaragoza, SPAIN | 480 | 41.25N, 1.35W | 23.3 | 5 | 434 | 44.6 |  |  |  |  |  |  |  |  |  |
| Xanthoparmelia verrucigera | 3 | MAF-Lich 15301 | Braganza, PORTUGAL | 1040 | 41.53N, 6.56W | 21 | 4.8 | 901 | 109.49 |  |  |  |  |  |  |  |  |  |
| Xanthoparmelia vicentei | 3 | MAF-Lich 7248 | Béjar, Salamanca, SPAIN | 1200 | 40.22N, 5.46W | 19.6 | 3 | 884 | 116.27 |  |  |  |  |  |  |  |  |  |
| Xanthoparmelia vicentei | 3 | MAF-Lich 9936 | Arroyo del zape, Zamora, SPAIN | 669 | 41.28N, 5.47W | 23.6 | 4.2 | 443 | 39.48 |  |  |  |  |  |  |  |  |  |
| Xanthoparmelia vicentei | 3 | MAF-Lich 9954 | El Castanar, Salamanca, SPAIN | 644 | 40.29N, 5.59W | 26.7 | 6.1 | 728 | 61.46 |  |  |  |  |  |  |  |  |  |
