# Supplementary material for: Accelerated evolutionary rates in tropical and oceanic parmelioid lichens (Ascomycota)
Source: BMC Evol Biol. 2008 Sep 22;8:257. doi: 10.1186/1471-2148-8-257 (PMC2564941; doi:10.1186/1471-2148-8-257)
Supplement: Additional file 5 — S5 – Species and specimens used in the current study with Genbank accession numbers. includes data of sequences used in the analyses and their specimens. [file 1471-2148-8-257-S5.doc]

| **Species** | **Voucher specimens** | **Locality** | **Collector (s)** | **mt SSU** | **nu ITS** | **nu LSU** |
| --- | --- | --- | --- | --- | --- | --- |
| *Bulbothrix apophysata* | F | Costa Rica | Lücking 16650b | DQ287788 | DQ279481 | EU562670 |
| *B. coronata* | MAF-Lich 13987 | South Africa | Crespo & al. | DQ287789 | DQ279482 | **EU562671** |
| *B. decurtata* | MAF-Lich 13988 | South Africa | Crespo & al. | DQ287790 | DQ279483 | **EU562672** |
| *B. goebelii* | MAF 13985 | South Africa | Lumbsch | DQ287791 | DQ279484 | **EU562673** |
| *B. meizospora* | GPGC 02-000786 | India | Divakar s.n. | AY611127 | AY611068 | AY607780 |
| *B. setschwanensis* | MAF-Lich 10212 | China | Crespo et al. | - | AY611069 | AY607781 |
| *Canoparmelia* *crozalsiana* | MAF-Lich 7658 | Spain | Crespo et al. | AY586594 | AY586571 | AY584831 |
| *Cetrelia chicitae* | - | Philippines | Bawingan CL 0650 | DQ923629 | DQ980006 | DQ923658 |
| *C. olivetorum* | UPS | United Kingdom | Wedin 6272 | DQ923630 | DQ980007 | DQ923659 |
| *Everniastrum cirrhatum* 1 | - | Costa Rica | Trest 149 | AY611128 | AY611070 | AY607782 |
| *E. cirrhatum 2* | MAF-Lich 13976 | Peru | Lumbsch 19342r | DQ287795 | DQ279487 | EU562674 |
| *E. lipidiferum* | MAF-Lich 13966 | Peru | Lumbsch 19309b | DQ287796 | DQ279488 | **EU562675** |
| *E. nepalense* | GPGC 02-000924 | India | Divakar | AY611129 | AY611071 | AY607783 |
| *E. rhizodendroideum* | ABL | China | Aptroot 55665 | DQ287797 | DQ279489 | EU562676 |
| *E. sorocheilum* | MAF-Lich 10375 | China | Crespo, Blanco & Argüello | DQ287798 | DQ279490 | EU562677 |
| *E. vexans* | ABL | China | Aptroot 56597 | DQ287799 | DQ279491 | **EU562678** |
| *Flavoparmelia* *baltimorensis* | MAF-Lich 7660 | USA | Molina | AY586583 | AY586559 | AY584832 |
| *F. caperata* | MAF-Lich 6045 | Spain | Crespo & al. | AF351163 | AY581059 | AY578922 |
| *F. soredians* | MAF-Lich 10176 | Spain | Crespo & al. | AY586586 | AY586562 | AY584835 |
| *F. springtonensis* | MAF-Lich 14271 | Australia | Elix 31200 | EF025483 | EF042907 | EF042916 |
| *Flavopunctelia flaventior* | MAF-Lich 6046 | Spain | Crespo & al. | AF351164 | AY581060 | AY578923 |
| *Hypotrachyna adducta* | MAF-Lich 10206 | China | Crespo, Blanco & Argüello | AY785277 | AY785270 | AY785263 |
| *H. booralensis* | MAF –Lich 13969 | Australia | Lumbsch | DQ287801 | DQ279493 | **EU562682** |
| *H. ciliata* | MAF-Lich 10185 | China | Crespo, Blanco & Argüello | AY785280 | AY785273 | AY785266 |
| *H. costaricensis* | MAF-Lich 10211 | Costa Rica | Molina | AY785276 | AY785269 | AY785262 |
| *H. crenata* | MAF-Lich 10377 | China | Crespo, Blanco & Argüello | DQ287804 | DQ279495 | **EU562683** |
| *H. endochlora* | MAF-Lich 10178 | United Kingdom | Coppins | AY611130 | AY611072 | AY607784 |
| *H. exsecta* | MAF-Lich 10380 | China | Crespo, Blanco & Argüello | DQ287807 | DQ279498 | **EU562684** |
| *H. flexilis* | MAF-Lich 13975 | India | Divakar | DQ287809 | DQ279500 | **EU562685** |
| *H. imbricatula* | MAF-Lich 13990 | South Africa | Crespo & al. | DQ287812 | DQ279503 | **EU562686** |
| *H. immaculata* | MAF-Lich 7462 | Australia | Louwhoff, Molina & Elix | **EU562696** | AY611073 | AY607785 |
| *H. incognita* | MAF-Lich 10385 | China | Crespo, Blanco & Argüello | DQ287815 | DQ279506 | **EU562687** |
| *H. infirma* | MAF-Lich 10210 | China | Crespo, Blanco & Argüello | AY785278 | AY785271 | AY785264 |
| *H. koyaensis* | MAF-Lich 10388 | China | Crespo, Blanco & Argüello | DQ287819 | DQ279509 | **EU562688** |
| *H. laevigata* | MAF-Lich 10177 | Great Britain | Coppins | AY611132 | AY611074 | AY607786 |
| *H. neodissecta* | MAF-Lich 13986 | South Africa | Crespo & al. | DQ287820 | DQ279510 | **EU562689** |
| *H. osseoalba* | MAF-Lich 10390 | China | Crespo, Blanco & Argüello | DQ287822 | DQ279512 | **EU562690** |
| *H. physcioides* | MAF-Lich 10391 | China | Crespo, Blanco & Argüello | DQ287823 | DQ279513 | **EU562691** |
| *H. pseudosinuosa* | MAF-Lich 10392 | China | Crespo, Blanco & Argüello | DQ287826 | DQ279516 | **EU562692** |
| *H. revoluta* | MAF-Lich 6047 | Spain | Noya & Olea | AF351166 | AY611075 | AY607787 |
| *H. rockii* | MAF-Lich 13965 | Peru | Lumbsch 19342l | DQ287834 | DQ279524 | **EU562693** |
| *H. scytophylla* | MAF-Lich 10410 | China | Crespo, Blanco & Argüello | DQ287835 | DQ279525 | **EU562694** |
| *H. sinuosa* | MAF-Lich 10179 | United Kingdom | Coppins | AY611133 | AY611076 | AY607788 |
| *H. taylorensis* | MAF-Lich 9921 | United Kingdom | Hawkswoth | AY582298 | AY581061 | AY578924 |
| *H. aff. brevirhiza* | MAF-Lich 10376 | China | Crespo, Blanco & Argüello | DQ287803 | DQ279494 | **EU562679** |
| *H. aff. immaculata* | MAF-Lich 10413 | China | Crespo, Blanco & Argüello | DQ287814 | DQ279505 | **EU562680** |
| *H. aff. taylorensis* | MAF-Lich 10409 | Canary Island | Crespo | DQ287839 | DQ279529 | **EU562681** |
| *Karoowia saxeti* | EBL | Taiwan | Aptroot 53350 | AY582299 | AY581063 | AY578926 |
| *Melanelixia fuliginosa* | MAF-Lich 7640 | Spain | Crespo & al. | AY611141 | AY611084 | AY607796 |
| *M. glabra* | MAF-Lich 7634 | Spain | Hawksworth | AY582300 | AY581064 | AY578927 |
| *M. subargentifera* | MAF-Lich 9909 | Spain | Crespo & al. | AY582301 | AY581065 | AY578928 |
| *M. subaurifera* | MAF-Lich 10217 | Spain | Blanco & Divakar | AY611157 | AY611100 | AY607812 |
| *Melanohalea elegantula* | MAF-Lich 10218 | Spain | Crespo & Divakar | AY611135 | AY611078 | AY607790 |
| *M. exasperata* | MAF-Lich 7636 | Spain | Crespo & al. | AY611140 | AY611083 | AY607795 |
| *M. exasperatula* | MAF-Lich 10213 | Spain | Crespo & al. | AY611147 | AY611090 | AY607802 |
| *M. olivacea* | H | Findland | Vitikainen 16196 | AY611148 | AY611091 | AY607803 |
| *M. septentrionalis* | H | Findland | Ahti 60893 | AY611150 | AY611093 | AY607805 |
| *M. subelegantula* | NDA | USA | Esslinger 16132 | AY611171 | AY611115 | AY607829 |
| *M. subolivacea* | NDA | USA | Esslinger 16555 | AY611178 | AY611123 | AY607837 |
| *M.* aff*. elegantula* | NDA | USA | Esslinger 16550 | AY611172 | AY611116 | AY607830 |
| *M.* aff. *exasperata* | MAF-Lich 10227 | Spain | Blanco | AY611139 | AY611082 | AY607794 |
| *Myelochroa irrugans* | MAF-Lich 10207 | China | Crespo & al. | AY611160 | AY611103 | AY607815 |
| *M. metarevoluta* | MAF-Lich 10208 | China | Crespo & al. | AY611159 | AY611102 | AY607814 |
| *Parmelaria subthomsonii* | LWG 20-77151 | India | Chatterjee & Divakar | AY586588 | AY586564 | AY584836 |
| *Parmelia discordans* | MAF-Lich 10232 | United Kingdom | Hawksworth | DQ287841 | AY583212 | EF042918 |
| *P. pinnatifida* | MAF-Lich 7272 | Rusia | Schlensog | AY611161 | AY036988 | - |
| *P. saxatilis* | MAF-Lich 6804 | Sweden | Ott | AF351172 | AF350027 | AY578947 |
| *P. serrana* | MAF-Lich 9756 | Spain | Crespo & Divakar | AY582319 | AY295109 | AY578948 |
| *P. squarrosa* | MAF-Lich 7281 | Japan | -- | AY611162 | AY036975 | AY607816 |
| *P. sulcata* | MAF-Lich 6054 | United kingdom | Lambley | AY582320 | AY581083 | AY578949 |
| *Parmelina carporrhizans* | MAF-Lich 6057 | Spain | Crespo | AY611164 | AY611105 | AY607818 |
| *P. pastillifera* | MAF-Lich 6058 | Spain | Crespo | **EU562697** | AY611104 | AY607817 |
| *P. tiliacea* | MAF-Lich 6056 | Spain | Crespo | AF351173 | AY581084 | AY578950 |
| *Parmelinella wallichiana* | LWG 20-77171 | India | Chatterjee & Divakar | AY611165 | AY611106 | AY607819 |
| *Parmelinopsis cryptochlora* | MAF- 10398 | China | Crespo, Blanco & Argüello | DQ287845 | DQ279535 | **EU562695** |
| *P. horrescens* | MAF-Lich 9913 | Spain | Carballal | AY582321 | AY581085 | AY578951 |
| *P. minarum* | MAF-Lich 7639 | Spain | Crespo & al. | AY582322 | AY581086 | AY578952 |
| *P. neodamaziana* | MAF-Lich 10182 | Australia | Louwhoff & al. | AY611166 | AY611107 | AY607820 |
| *P. subfatiscens* | MAF-Lich 6878 | Australia | Louwhoff & al. | AF351174 | AY611108 | AY607821 |
| *Parmeliopsis ambigua* | GZU 46209 | Austria | - | **EU562698** | - | - |
| *P. ambigua* | - | - | Tehler 8110 | - | AF410829 | - |
| *P. ambigua* | MAF-Lich 10186 | Spain | Divakar | - | - | AY607822 |
| *P. hyperopta* | MAF-Lich 10181 | Spain | Blanco | AY611167 | AY611109 | AY607823 |
| *Parmotrema cetratum* | MVM | Uruguay | Osorio 9424 | AY586598 | AY586576 | AY584847 |
| *P. crinitum* | MAF-Lich 6061 | Portugal | Crespo | **EU562699** | AY586565 | AY584837 |
| *P. fistulatum* | MVM | Uruguay | Osorio 9423 | **EU562700** | AY581057 | AY578920 |
| *P. haitiense* | MAF-Lich 7657 | Australia | Louwhoff, Molina & Elix | AY582295 | AY581055 | AY578918 |
| *P. hypoleucinum* | MAF-Lich 7636 | Spain | Crespo & al. | AY586590 | AY586567 | AY584839 |
| *P. perforatum* | - | USA | Cole 7983 | AY586591 | AY586568 | AY584840 |
| *P. perlatum* | MAF-Lich 6965 | Portugal | Crespo & al. | AY586580 | AY586566 | AY584838 |
| *P. pilosum* | MAF-Lich 7656 | Uruguay | Sacarabino | **EU562701** | AY581056 | AY578919 |
| *P. pseudoreticulatum* | MAF-Lich 7650 | Spain | Crespo | AY586600 | AY586578 | AY584849 |
| *P. reticulatum* | MAF-Lich 6067 | Portugal | Crespo & Jones | AF351184 | AY586579 | AY584850 |
| *P. robustum* | MAF-Lich 10166 | Portugal | Crespo & al. | **EU562702** | AY586569 | AY584841 |
| *P. subcaperatum* | HO 324283 | Australia | Kantvilas | **EU562703** | AY586557 | AY584829 |
| *P. subtinctorum* | GPGC 02-000696 | India | Divakar | AY586582 | AY586558 | AY584830 |
| *P. tinctorum* | MAF-Lich 10163 | Australia | Louwhoff, Molina & Elix | AY586593 | AY586570 | AY584842 |
| *Punctelia borreri* | MAF-Lich 9919 | Portugal | Crespo & al. | AY582324 | AY581088 | AY578954 |
| *P. pseudocoralloidea* | MAF-Lich 6922 | Australia | Louwhoff, Molina & Elix | AY586595 | AY586572 | AY584843 |
| *P. rudecta* | MAF-Lich 7661 | USA | Molina | AY586596 | AY586573 | AY584844 |
| *P. subflava* | Elix 42705 | Australia | Elix | **EU562704** | AY586575 | AY584846 |
| *P. subrudecta* | MAF-Lich 9918 | Portugal | Crespo & al. | AY582325 | AY581089 | AY578955 |
| *Xanthoparmelia conspersa* | MAF-Lich 6793 | Spain | Blanco & Crespo | AF351186 | AY581096 | AY578962 |
| *X. crespoae* | MAF-Lich 7524 | Australia | Louwhoff, Molina & Elix | AY582332 | AY581097 | AY578963 |
| *X. delisei* | MAF-Lich 7659 | Spain | Blanco & Crespo | **EU562705** | AY581068 | AY578931 |
| *X. digitiformis* | MAF-Lich 7525 | Australia | Louwhoff, Molina & Elix | AY582334 | AY581099 | AY578965 |
| *X. glabrans* | MAF-Lich 7665 | Australia | Louwhoff, Molina & Elix | AY582305 | AY581069 | AY578932 |
| *X. hueana* | GZU 46511 | Namibia | Baule | AY582326 | AY581090 | AY578956 |
| *X.* isidio*vagans* | MAF-Lich 9956 | Spain | Crespo & Divakar | AY582330 | AY581094 | AY578960 |
| *X. lithophila* | MAF-Lich 6900 | Australia | Louwhoff, Molina & Elix | **EU562706** | AY581077 | AY578941 |
| *X. loxodes* | MAF-Lich 7072 | Spain | Crespo & al. | AY582313 | AY581076 | AY578940 |
| *X. mougeotii* | MAF-Lich 9916 | Spain | Blanco & Crespo | AY582336 | AY581100 | AY578967 |
| *X. murina* | MAF-Lich 9915 | Australia | Louwhoff, Molina & Elix | AY582315 | AY581079 | AY578943 |
| *X. norcapnodes* | MAF-Lich 7532 | Australia | Louwhoff, Molina & Elix | AY582316 | AY581080 | AY578944 |
| *X. notata* | CANB | Australia | Elix 42648 | AY582337 | AY581101 | AY578968 |
| *X. pokornyi* | MAF-Lich 9908 | Spain | Divakar | **EU562707** | AY581075 | AY578939 |
| *X. protomatrae* | MAF-Lich 6216 | Spain | Blanco & Crespo | AY582339 | AY581104 | AY578972 |
| *X. pulla* | MAF-Lich 6794 | Spain | Crespo | AF351169 | AY581071 | - |
| *X. pulla* | - | Poland | - | - | - | AJ421433 |
| *X. pulloides* | MAF-Lich 6784 | Spain | Blanco & Crespo | AY582309 | AY037004 | AY578936 |
| *X. scotophylla* | CANB | Australia | Elix 30650 | AY582317 | AY581081 | AY578945 |
| *X. semiviridis* | CANB | Australia | Elix 30294 | AF351158 | AY581058 | AY578921 |
| X. stenophylla | MAF-Lich 9917 | Spain | Crespo & al. | AY582329 | AY581093 | AY578959 |
| *X. subincerta* | MAF-Lich 7494 | Australia | Louwhoff, Molina & Elix | AY582310 | AY581073 | AY578937 |
| *X. subprolixa* | MAF-Lich 7667 | Australia | Louwhoff, Molina & Elix | AY582311 | AY581074 | AY578938 |
| *X. subspodochroa* | MAF-Lich 7463 | Australia | Louwhoff, Molina & Elix | AY582318 | AY581082 | AY578946 |
| *X. tegeta* | MAF-Lich 7523 | Australia | Louwhoff, Molina & Elix | **EU562708** | AY581107 | AY578975 |
| *X. tinctina* | BCN-13862 | Spain | Llimona | AY582345 | AY581110 | AY578978 |
| *X. transvaalensis* | MAF-Lich 9841 | Spain | Blanco & Crespo | AY582331 | AY581095 | AY578961 |
| *X. verrucigera* | MAF-Lich 9920 | Spain | Crespo & Molina | AY582346 | AY581111 | AY578979 |
| *X. vicentei* | MAF-Lich 7248 | Spain | Crespo & Molina | AY582347 | AY581112 | AY578980 |
| *Usnea antarctica* | F | Antarctica | Lumbsch 19029c | EF116571 | EF116567 | EF116569 |
| *U. florida* | UPS | Sweden | Mattsson 4001 | AJ457147 | EF113545 | EF113546 |
| *U. trachycarpa* | F | Argentina | Lumbsch 19001a | EF116572 |  | EF116570 |
| *U. trachycarpa* | F | Argentina | Wirth & Messuti | - | EF116568 | - |
